# Green Space and Health Equity: A Systematic Review on the Potential of Green Space to Reduce Health Disparities

**DOI:** 10.3390/ijerph18052563

**Published:** 2021-03-04

**Authors:** Alessandro Rigolon, Matthew H. E. M. Browning, Olivia McAnirlin, Hyunseo (Violet) Yoon

**Affiliations:** 1 Department of City and Metropolitan Planning, The University of Utah, Salt Lake City, UT 84112, USA; 2 Department of Parks, Recreation and Tourism Management, Clemson University, Clemson, SC 29634, USA; mhb2@clemson.edu (M.H.E.M.B.); omcanir@g.clemson.edu (O.M.); 3 Department of Recreation, Sport and Tourism, University of Illinois at Urbana-Champaign, Champaign, IL 61820, USA; hyunseo2@illinois.edu

**Keywords:** atopic disease, birth outcomes, cardiovascular disease, diabetes, green infrastructure, mortality, normalized difference vegetation index (NDVI), obesity

## Abstract

Disadvantaged groups worldwide, such as low-income and racially/ethnically minoritized people, experience worse health outcomes than more privileged groups, including wealthier and white people. Such health disparities are a major public health issue in several countries around the world. In this systematic review, we examine whether green space shows stronger associations with physical health for disadvantaged groups than for privileged groups. We hypothesize that disadvantaged groups have stronger protective effects from green space because of their greater dependency on proximate green space, as they tend to lack access to other health-promoting resources. We use the preferred reporting items for systematic reviews and meta-analyses (PRISMA) method and search five databases (CINAHL, Cochrane, PubMed, Scopus, and Web of Science) to look for articles that examine whether socioeconomic status (SES) or race/ethnicity modify the green space-health associations. Based on this search, we identify 90 articles meeting our inclusion criteria. We find lower-SES people show more beneficial effects than affluent people, particularly when concerning public green spaces/parks rather than green land covers/greenness. Studies in Europe show stronger protective effects for lower-SES people versus higher-SES people than do studies in North America. We find no notable differences in the protective effects of green space between racial/ethnic groups. Collectively, these results suggest green space might be a tool to advance health equity and provide ways forward for urban planners, parks managers, and public health professionals to address health disparities.

## 1. Introduction

Disadvantaged groups around the world, including low-income and racially/ethnically minoritized people, experience a higher prevalence of certain diseases than more privileged groups, including more affluent and white people [1,2,3]. Scholars have defined such disproportionate disease rates as health disparities, inequalities, or inequities [1,2,4,5] and have attributed them to poverty, subpar education, inadequate health care, and harmful environmental exposures for disadvantaged groups [2,6]. Health disparities are a particularly glaring issue in the United States (U.S.) [7], where, on average, individuals in the highest income bracket live six years longer than those in the lowest bracket [4], and black people live four fewer years than non-Hispanic white people [8]. Studies in many other countries with large shares of white populations (e.g., England, Australia, and South Africa) have found similar disparities based on race/ethnicity [9,10,11]. Due to these disparities, scholars and public health practitioners have advocated for efforts to achieve health equity [1,2,12,13]. Health equity describes the elimination of health disparities based on social or economic disadvantage and of their root causes, such as poverty and racial discrimination [1,2].

A growing body of literature shows green space (e.g., trees and parks) can have positive impacts on human health [14,15]. For instance, exposure to green space in residential environments is associated with better general health [16], higher birth weight [17], and lower mortality [18]. The health-promoting effects of green space have been explained by its ability to reduce exposure to air pollution and other harmful environmental exposures (mitigation), encourage healthy behaviors such as physical activity and sleep (instoration), and provide relief for cognitive processes and stress (restoration) [19,20].

Some evidence suggests green space has larger health benefits for some sociodemographic groups than for others [21,22,23,24]. Indeed, socioeconomic and racial/ethnic vulnerabilities are commonly discussed moderating factors in proposed frameworks for studying the health benefits of nature contact [25,26,27,28]. We are not aware of reviews that have attempted to specifically examine these topics despite repeated calls for these reported effect modifiers [20,27]. The research that most closely addresses these calls is a systematic review of papers published through 2017 by Kabisch [28]. In this review, the author qualitatively describes relevant empirical studies and concludes that socioeconomic and sociodemographic factors are relevant modifiers or confounders of the health benefits of green space exposure; no quantitative synthesis was conducted.

Other reviews have also qualitatively described whether effect modification by socioeconomic status (SES) or race/ethnicity exists within certain domains of health. In particular, at least four literature reviews found that green space might have different benefits for birth outcomes depending on socioeconomic status (SES) and geography, although consensus regarding the direction of these effects (e.g., favoring lower SES groups) has not crystallized [17,29,30,31]. Another review described how some studies found low-SES groups showed greater health benefits if they lived in a greener neighborhood, relative to other populations [19].

The possibility of certain populations benefiting most from green space could be linked to another inequity: disadvantaged populations having less access to urban green space than more privileged groups in both Global North and Global South cities [32,33,34,35]. This intersection between health and environmental inequity warrants investigation into who benefits most from green space.

### 1.1. Research Questions and Hypotheses

In this review, we begin to clarify whether green space shows stronger associations with physical health for disadvantaged populations than for privileged groups. Here, disadvantaged individuals include those with low SES or who identify as a racially/ethnically minoritized group (i.e., in most Global North contexts, as not White). We advance the work of previous reviews by systematically analyzing and quantifying whether SES and race/ethnicity modify the associations between green space and several health outcomes. We ask four research questions that shed light on whether, and under which conditions, green space has the potential to help reduce health disparities.

- RQ1. Does green space show more protective effects for disadvantaged populations than for privileged groups?
- RQ2. What type(s) of green space (e.g., trees or parks) shows more protective effects for disadvantaged populations than for privileged groups?
- RQ3. Does green space show more protective effects on specific diseases and illnesses for disadvantaged populations than for privileged groups?
- RQ4. In which continent(s) does green space show more protective effects for disadvantaged populations than for privileged groups?

The first is the main question of this study. The second could provide preliminary information about which greening interventions have stronger protective effects for disadvantaged groups, which might inform cross-sectoral collaborations between public health and urban planning professionals [36,37,38]. The third and fourth could shed further light about under which conditions green space shows stronger protective effects for disadvantaged people. Specifically, RQ4 could provide information about in which sociopolitical contexts green space might be used as a strategy to help reduce health disparities, since scholars have suggested the importance of considering such contexts when planning health equity initiatives [2,12].

We hypothesize that green space does indeed provide stronger protective effects for disadvantaged populations compared to privileged groups (H1). We also hypothesize that measures of green land cover (e.g., NDVI greenness and tree canopy) show stronger effects for disadvantaged groups than park access (H2). Given the empirical and theoretical evidence to-date, we assert there is not enough information to formulate hypotheses regarding RQ3 and RQ4.

Rationale for H1 is provided by research about suppressed baselines, neighborhood dependency, and a lack of other health-promoting spaces. Regarding suppressed baselines, disadvantaged populations experience worse health and are exposed to greater environmental pollution than other groups, on average [4,39,40]. Since disadvantaged populations have a lot to gain regarding improvements in health and reductions in pollutants, neighborhood characteristics that promote health could show particularly strong effects [41,42]. Regarding neighborhood dependency, low-SES populations are generally less mobile due to lower vehicle ownership and spend more time in their residential neighborhoods [43,44,45]. Regarding the lack of other health-promoting settings, disadvantaged populations might not have access to private recreation/exercise opportunities like gyms or a backyard [44,45]. Thus, nearby accessible green space might have more beneficial impacts for disadvantaged people than for more privileged people. Furthermore, we expect that H1 will be supported because a recent review on green space and health found some studies showing greater protective effects from green space for low-SES than for high-SES people [15].

Regarding H2, parks in low-SES and racially/ethnically minoritized neighborhoods tend to be of lower quality (e.g., fewer amenities and lower maintenance) and have higher crime rates than parks in more privileged communities [32,33,46,47]. Specifically, studies around the world found SES and racial/ethnic inequities in park quality related to the number and types of park amenities [32,33]. Additionally, much of the work finding evidence of inequities in park safety has focused on the U.S. (see [47]). When parks are of low quality or are unsafe, studies in the U.S. show that some people choose not to access them and/or engage in less physical activity in them [48,49]. Perhaps as a result of that, research has shown associations between low park quality and low health status in North American contexts [48,50]. When focusing on measures of green land cover, we are not aware of studies showing disparities in the quality of vegetation (e.g., shrubs or trees) between privileged and disadvantaged communities. Therefore, because there is evidence of disparities in park quality but not in the quality of green land cover, we expect parks to have weaker protective effects for disadvantaged groups than measures of green land cover.

To answer our four research questions, we systematically review the literature on the protective effects of green space for the physical health outcomes for which there is evidence of disparities around the world (e.g., [8,51]): atopic diseases, birth outcomes, cancer, cardiovascular health/disease, diabetes, general health, mortality, and obesity (see Section 2.2). We search for studies that report differences in the health benefits of green space for individuals or neighborhoods based on SES or race/ethnicity.

### 1.2. Health Disparities, Health Inequities, and Health Equity

#### 1.2.1. Defining Terms

Before presenting the methods and results of our systematic review, we introduce the definitions of health disparities, health inequities, and health equity, and we review the major health disparities based on SES and race/ethnicity. In a recent report, Braveman and colleagues defined *health disparities* (or inequalities) as “plausibly avoidable, systematic health differences adversely affecting economically or socially disadvantaged groups” ([1], p. 11). Regardless of whether these differences are linked to structural injustices, they negatively impact the lives of people already affected by social or economic disadvantage, and therefore raise ethical concerns [1,52,53].

Health disparities have been linked to several determinants, including disadvantaged populations having less wealth, worse educational opportunities, less access to health care, and more harmful environmental exposures in residential and work settings than privileged groups [2,3,6,41]. Specifically, decades of research have shown that low-SES people and racially/ethnically minoritized people are disproportionately exposed to hazardous environments, such as power plants, oil refineries, high-traffic roadways, and landfills [54,55,56]. Such exposures contribute to worse health by increasing air pollution, noise, and heat [54,55,56].

*Health inequities* involve the recognition that differences in health outcomes are the result of unjust policies and societal structures [1,2,12,52]. Specifically, Braveman and colleagues state: “A health inequity is a particular kind of health disparity […] that is not only of concern for being potentially unfair, but which is believed to reflect injustice” ([1], p. 12). The most complex aspect of differentiating a health disparity from a health inequity is identifying sufficient evidence about unjust or deliberately harmful causes of such disparity [1,52]. Since racially/ethnically minoritized people are disproportionately exposed to environmental hazards (e.g., air pollution) and suffer from health disparities linked to exposure to such hazards (e.g., lung disease), those health disparities can be considered as health inequities [1,52]. In the U.S. and other majority-white countries, the link between harmful exposures and health inequities has been attributed to systemic racism [57,58]. Despite this research, there is little scholarly agreement about how much and which type of evidence warrants calling a health disparity a health inequity [1]. As such, in this paper, we use the phrase health disparities.

*Health equity* describes the reduction and eventual elimination of health disparities affecting disadvantaged groups, and the removal of the social determinants of health disparities, such as racial discrimination, poverty, and the lack of high-quality education [1,41]. In other words, “Health equity is the ethical and human rights principle motivating efforts to eliminate health disparities; health disparities are the metric for assessing progress toward health equity” ([1], p. 12). Since research showed that inequalities in exposure to environmental hazards contribute to health disparities [55,56], some scholars argued that environmental exposures such as green space can help move the needle toward health equity by mitigating hazardous exposures and providing other health-protective effects [38,59].

#### 1.2.2. Identifying Health Disparities

To gather evidence on health disparities around the world, we retrieved sources on this topic from governmental organizations, nonprofits, and scholars. This effort was not intended as a systematic review on health disparities, but to provide guidance on which health outcomes to include in this review. We focused on disparities based on SES and race/ethnicity because—based on the sources we retrieved—low-SES people and racially/ethnically minoritized people were the sociodemographic groups most often impacted by disparities [2,4,8,60]. Additionally, low-SES people and racially/ethnically minoritized people have been most often the populations of interest in environmental justice studies on the provision of green space [32,35]. Thus, we did not focus on health disparities based on sexual orientation, gender identity, veteran status, and disability.

Table 1 below summarizes the main health disparities we identified through our search. We reported those disparities that represent differences between privileged and disadvantaged groups that are either supported by citations in any of the sources we identified or that are reported as statistically significant in these sources (see notes in Table 1). To finalize the table, two authors cross-checked their evaluations of these reports to determine what disparities exist based on SES and race/ethnicity.

## 2. Materials and Methods

### 2.1. Article Search

**Figure 1:**
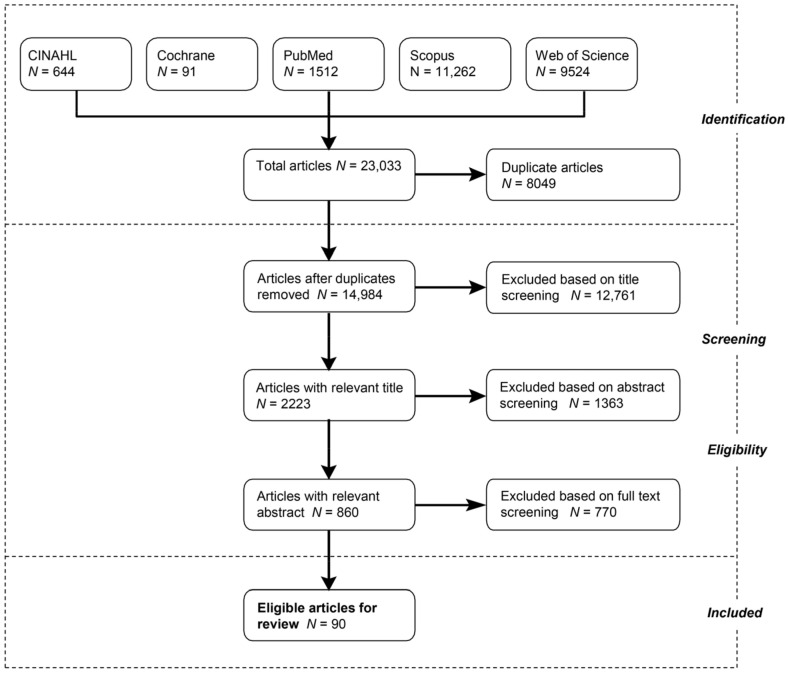
Search, screening, and selection process following the preferred reporting items for systematic reviews and meta-analyses (PRISMA) protocol.

We adopted the standards for systematic reviews described in the preferred reporting items for systematic reviews and meta-analyses (PRISMA) [66] (Figure 1). The PRISMA checklist for this paper is reported in Table S13. To retrieve relevant articles, we reviewed five databases: CINAHL, Cochrane, PubMed, Scopus, and Web of Science. Additionally, we selected keywords from previous systematic reviews on green space and health and access to green space (see Table S2). Inclusion criteria included:

1. Report at least one of the measures of physical health outcomes described in the introduction (atopic diseases, birth outcomes, cancer, cardiovascular health/disease, diabetes, general health, mortality, and obesity);
2. Report at least one measure of objective or perceived green space provision or exposure. Green space measures include access to parks, level of residential vegetation (e.g., greenness), access to a residential garden, and others [67];
3. Perform inferential statistical analyses (e.g., regressions) on primary data;
4. Find that either the entire study population or a subsample (e.g., high-SES people) shows a beneficial relationship between green space and health;
5. Analyze whether the size or direction of the association between green space and physical health differs between disadvantaged and privileged populations (e.g., split-sample analyses or interaction tests);
6. Be published in peer-reviewed journals and written in English.

We conducted all searches on 17 April 2019, and then screened the titles, then the abstracts, and finally the full texts of the papers we identified through the search. All four authors participated in the screening process. At least two authors screened each title, abstract, and full text for inclusion, and they resolved disagreements about whether a paper was to be included or excluded by discussing each paper in relation to the above inclusion criteria. This ensured that at least two researchers agreed on whether an article was to be included. Among the 860 papers identified for full-text screening, 405 articles reported positive associations between green space and health, whereas 95 articles reported null or negative associations (other articles did not study the green space-health association or were literature reviews). The screening process resulted in 90 articles meeting the inclusion criteria: 85 papers examined effect modification by SES and 25 by race/ethnicity (20 papers examined effect modification for both SES and race/ethnicity). For most steps described in the method section, additional details are provided in the Methodological Details (see Supplementary Materials).

### 2.2. Data Extraction

Next, we extracted data from the 90 included journal articles, including the study design, continent and country, sample, health outcome(s), green space type, results of the effect modification tests, and analysis of methodological bias and other ancillary attributes (see Table S4 for a complete list). As for the data screening process, all four authors participated in the data extraction. At least two authors worked independently to extract data from each included article, and they resolved disagreements in how they coded specific elements (e.g., green space type) by referring to the codebook presented in Table S4.

Similar to other recent systematic reviews on green space [68,69], we entered multiple rows in the spreadsheet for the same article if the article included more than one of the following characteristics: (1) research design (i.e., cross-sectional or longitudinal); (2) health outcome type; (3) green space type; (4) distance between the study’s unit of analysis and green space; (5) green space measurement, including objective (e.g., GIS) or subjective evaluations; or (6) cities or countries where data were collected, if results were reported for separate samples. This process resulted in a greater number of rows than the number of articles alone since 28 articles were separated into multiple rows. Specifically, 122 rows of data reported effect modification by SES, and 30 rows of data reported effect modification by race/ethnicity.

We classified green space types into four categories based on previous literature [67,70,71]: green land cover, public green space, gardens, and nature-based programs (see Table S4). Green land cover was a broad category that referenced what many scholars call “green space” and encompassed any setting with live vegetation [70]. Therefore, studies assigned to this category were those that used satellite-derived indices (e.g., normalized difference vegetation index (NDVI)) or land use and land cover-derived (LULC) datasets to measure the coverage of natural areas within a geographic area [71]. Since these datasets were unable to distinguish where the green cover was located and for whom exposure was likely, green space measured within this category likely captured settings with specific purposes and allowed users (e.g., parks, greenways, and gardens) [20,72]. Other studies examined those settings with specific purposes and users exclusively, and we categorized these studies into the next two categories: public green space (e.g., parks) or private green space (e.g., gardens). Finally, in acknowledgment of the growing body of literature that examines the health benefits of green exercise and the potential for effect modification by SES or race/ethnicity in these studies [73], we considered investigations that assessed nature-based programs as a distinct category because such programs paired green space exposure (e.g., in a park) with specific activities (e.g., exercise) [69].

Our main measures of interest were the results of effect modification tests by SES and race/ethnicity. After examining recent systematic reviews, we did not find any robust methodological precedents to code the direction of effect modifications beyond counts of studies and directionality of findings (i.e., number of articles showing increased CVD risk with greater racial/ethnic segregation) [74]. Therefore, we created a novel method that accounts for the presence of multiple analyses within a single article [68] and, corresponding, a spectrum of possible findings in a given article: from harmful in all analyses to harmful in some analyses/null in all analyses/protective in some/protective in all analyses. The development of this method was based on in-depth discussions among the authors. We coded the effect modification as 1.0 if green space showed more protective effects for a disadvantaged group (e.g., low-SES or racially/ethnically minoritized people) than a privileged group; as 0.0 if there were no differences between disadvantaged and privileged groups; and as -1.0 if green space had more protective effects for privileged groups (e.g., high-SES or white people) than disadvantaged groups. As all studies analyzing effect modification by race/ethnicity focused on countries with a white population of European descent that holds political and economic power, we considered all other groups as racially/ethnically minoritized (e.g., Latinx people in the U.S., indigenous people in Australia).

The studies we included either reported effect modification as split-sample analyses (e.g., showing the green space-health association for separate groups of low-SES vs. high-SES people) or as interaction tests (e.g., multiplicative interaction terms between green space and SES measures). For interaction tests, we coded that there was a difference in the effect of green space between disadvantaged and privileged groups (e.g., value of 1.0 or -1.0) if the interaction term was significant (*p* < 0.05); otherwise, we coded the effect modification as 0.0. For split-sample analyses, we coded the effect modification as 1.0 or −1.0 in either of the following cases: one sample showed significant protective effects and the other did not; both samples showed significant protective effects, but one sample’s effects were more protective and their 95% confidence intervals did not overlap those of the other sample. Otherwise, we coded the effect modification as 0.0. When split-sample analyses included more than two groups (e.g., income quartiles), we examined whether there were differences in protective effects below/above the median or the mean (whichever was reported): for example, we coded the effect modification as 1.0 if quartile 2 of income showed more protective effects than quartile 3 of income.

Some articles reported both split-sample and interaction tests of effect modification for the same row of data (e.g., associations between public green space and diabetes). In these cases, we averaged the values of the effect modification codes that applied to each effect modification analysis. We also averaged effect modification (EM) values when articles reported several effect modification tests for the same health outcome type (e.g., both body mass index and waist circumference for obesity-related measures) or multiple effect modification tests for the same green space type (e.g., both park proximity and park acreage for public green space). In these circumstances, we averaged EM values because we did not have definitive information to weigh one condition more than another (e.g., park proximity and park acreage). As a result of averaging, effect modification values can assume any decimal value between −1.00 and 1.00 (e.g., −0.50 and 0.33). The process of assigning values for the effect modification in each row resulted in a good inter-rater agreement between two researchers extracting data for EM SES (82%) and an acceptable agreement for EM race/ethnicity (65%). The latter was due to inconsistent understandings among the research team about what constituted a racially/ethnically minoritized group in countries around the world during the first round of coding. We addressed this issue by reviewing definitions of what constitutes a minoritized group in the countries where the sample studies were set in subsequent rounds of coding, which were then used to compile the final dataset.

### 2.3. Methodological Bias and Quality of Evidence

We also evaluated the potential for methodological bias. Building on Radke et al.’s (2019) work [75], we developed a bias evaluation instrument including four categories that are relevant for the green space-health connection: (1) study design, (2) green space exposure, (3) use and justification of confounders, and (4) statistical analysis (see Table S4 and 2.1.5 in the Supplementary Materials for more details). Using these four categories, we assigned quality scores to each row of data representing an inferential statistical analysis, summed up the scores for each category (see Table S4), and calculated the percentage of total possible quality points for each row. Then, we averaged these percentages in articles with multiple rows of data. As for the rest of the data extraction process, at least two authors vetted each article’s scores for methodological bias. After this process, we classified studies based on five levels of quality: excellent (≥ 81% of the total possible quality points), good (60–80%), fair (40–60%), poor (20–40%), and very poor (<20%).

We also judged the quality of evidence by calculating the average quality score across the samples of papers evaluating EM by SES and EM by race/ethnicity. Following Radke et al.’s (2020) review on phthalate exposure [76], we judged the quality of evidence based on (1) the number of articles included in our review, (2) the quality of such studies, as determined by our methodological bias calculations, and (3) the pooled confidence of effects/effect size, as determined by our innovative way of combining EM results. Two authors worked on evaluating the quality of evidence of the included articles.

For each effect modification analysis (SES and race/ethnicity), we assigned a score of robust evidence for, moderate evidence for, slight evidence for, indeterminate evidence for, or compelling evidence against effect modification. Specifically, robust and moderate strength of evidence described evidence that clearly supported the effect modification in favor of one group more than another; these two categories were differentiated by the quantity and quality of relevant studies. Slight and indeterminate described evidence for which uncertainties prevented drawing a conclusion regarding effect modification, due to limited quantity, quality, or pooled effects; ultimately, these categories strongly indicate a need for additional research. Last, compelling evidence of no effect required several high-quality studies with consistently null results for effect modification tests.

### 2.4. Data Analysis

We analyzed articles reporting effect modification (EM) by SES (122 rows, 85 articles) and articles reporting EM by race/ethnicity (30 rows, 24 articles) separately using R (Version 4.02; R Core Team, Vienna, Austria) [77]. Statistical significance was indicated by *p* < 0.05. For RQ1, we computed descriptive statistics (means and standard deviations) for the EM values for SES and race/ethnicity, examining whether green space has more protective effects for low-SES versus high-SES people and for white versus racially/ethnically minoritized people. For RQ2-RQ4, we ran Kruskal–Wallis tests (stats package v3.6.2) followed by Dunn’s post-hoc pairwise comparisons (FSA package v0.8.30) to determine whether there were statistically significant differences in EM values based on the green space type (RQ2), health outcome type (RQ3), and continent (RQ4). We ran Kruskal–Wallis tests, rather than one-way ANOVA tests, because the EM values for SES and race/ethnicity were not normally distributed. Kruskal–Wallis tests are non-parametric analyses that compare mean ranks between three or more independent groups [78]. We ran post-hoc Dunn’s tests to examine whether there were statistically significant differences in the mean ranks of EM values between each pair of groups (e.g., North America vs. Europe for RQ4). We conducted Dunn’s tests regardless of whether Kruskal–Wallis tests were significant because we were interested in examining comparisons between specific groups [79,80]. To evaluate the extent to which bias was disproportionately present across our sample, and in particular, within those categories of interest for RQ2, RQ3, and RQ4, we compared quality scores between categories of rows for green space type, health outcome type, and continent. We then reported only those quality scores for pairwise comparisons that were significant and remained significant in all sensitivity analyses for parsimony (see below).

We conducted two sets of sensitivity analyses. In the first, we recoded the continuous EM values for SES and race/ethnicity into integers (−1, 0, and 1). We assigned -1 if the EM value was negative (−1 ≤ EM value < 0) and 1 if the EM value was positive (0 > EM value ≥ 1). As a sensitivity analysis for RQ1, we computed descriptive statistics (means and standard deviations) for the recoded EM values for SES and race/ethnicity. For RQ2-RQ4, we built contingency tables and used Fisher’s exact tests (stats package v3.6.2), because some cell counts had fewer than five observations [81], and post-hoc pairwise comparisons (rcompanion package v2.3.25), to determine whether, for example, the EM values were statistically significantly different based on the continent (RQ4). 

In the second sensitivity analysis, we removed the three rows that measured green space as nature-based programs, from the dataset. Through this analysis, we aimed to only focus on measures representing accessible green space and exclude measures representing structured activities in green space such as exercise. Using this dataset, we conducted the same tests described for the main analysis (i.e., descriptive statistics, Kruskal–Wallis tests, and Dunn’s tests).

### 2.5. Articles Finding Null or Negative Associations between Green Space and Health

We also examined the subsample of articles in which (a) the green space-health relationship was null or negative and (b) effect modification by SES and/or race/ethnicity was studied. We conducted this separate search and analysis as a robustness check to our main analysis (RQ1). One could imagine a situation where these studies showed that low-SES and/or racially/ethnically minoritized people experienced more harmful impacts than privileged groups. Such a hypothetical situation would counterbalance the expected findings of our main analysis, regarding low-SES and/or racially/ethnically minoritized people experiencing more beneficial impacts than privileged groups.

For this analysis, we rescreened the full texts of the 860 articles with relevant abstracts to identify articles with null or negative findings for effect modification. Through this process, we retrieved seven relevant articles, all of which found null associations between green space and health. Five of these examined effect modification by SES, and two of these studied effect modification by race/ethnicity. We analyzed the sign of the effect modification tests following the same process described for the main sample to examine whether harmful effects were stronger amongst SES or racial/ethnic groups.

## 3. Results

### 3.1. Descriptive Statistics

**Figure 2:**
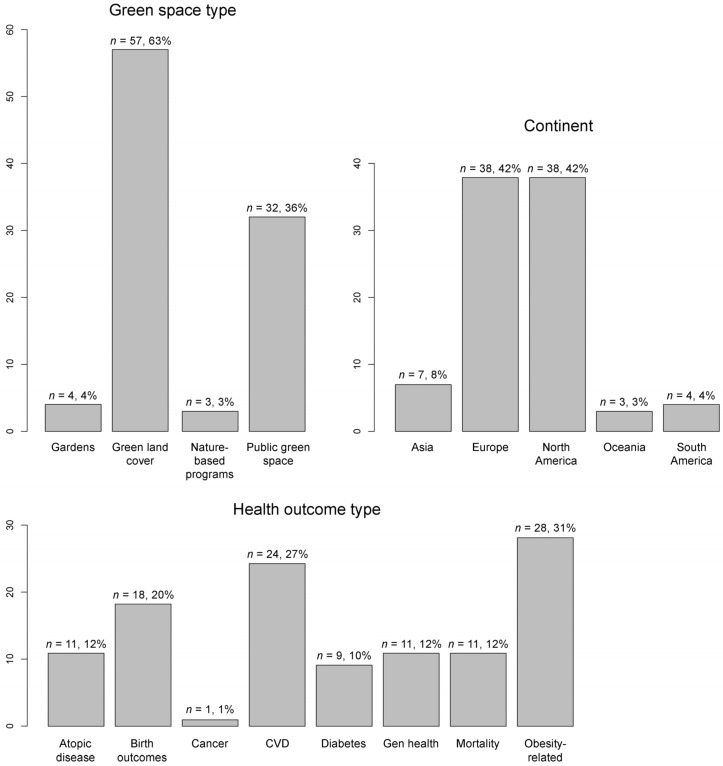
Descriptive statistics for the 90 included papers. CVD = cardiovascular health/disease. Gen health = general health.

Table 2 lists the articles included in this review, and key characteristics related to this study. As noted earlier, more articles reported effect modification (EM) by SES (*n* = 85, 94%) than EM by race/ethnicity (*n* = 25, 28%). Figure 2 displays the descriptive statistics for key characteristics of the 90 included studies. The green space type that was studied most frequently was green land cover followed by public green spaces. The most commonly studied health outcome types were obesity-related measures and cardiovascular health/disease. We found studies focusing on people living in every continent except for Africa, and most articles analyzed settings in North America (principally, the U.S.) and Europe; therefore, the results may best speak to the potential for effect modification within these two regions, and continental comparisons may speak only to differences in effects between U.S. and European populations. The sample size of these studies ranged between 106 (individual-level study) and 97,574,613 (ecological study). None of the included studies were experimental, most were cross-sectional (*n* = 79, 88%), and few were longitudinal (*n* = 11, 12%). The articles were published between 2003 and 2019, and most (*n* = 83, 92%) in the 2012–2019 period (see Figure S1).

In addition to the green space categories, other measures of green space exposure also varied quite considerably in our sample. Among all analyses focusing on EM by SES and/or race/ethnicity, 90 percent operationalized green space through objective measures (e.g., remote sensing), whereas the remaining 10 percent relied on subjective measures (e.g., surveys). Regarding the units of analyses, 85 percent focused on individuals, 9 percent on neighborhoods (e.g., census tracts in the U.S. and Lower Layer Super Output Areas in England/Wales), and 6 percent on cities or counties. Among the analyses using geographic information systems to measure green space exposures, 73 percent used a distance-based approach (e.g., radial buffers around homes, distances to parks) and the remaining 27 percent a container approach (see [71,82,83]). Additionally, of the analyses using remotely sensed raster datasets (e.g., Landsat/MODIS-derived NDVI), 70 percent used resolutions between 2 and 30 m^2^, 23 percent used resolutions larger than 250 m^2^, and 7 percent used resolutions of 1 m^2^ or smaller (no papers used resolutions between 31 and 250 m^2^).

### 3.2. Summary of Findings on Methodological Bias and Quality of Evidence

The analysis of methodological bias highlighted that most studies were either of good quality (*n* = 50, 56%) or fair quality (*n* = 39, 43%), whereas only one study was of poor quality (see Table S5). Frequent possible bias included observational cross-sectional designs, the lack of control for spatial autocorrelation, and the absence of sensitivity analyses. Specifically, among the four categories, we included in our bias evaluation instrument, research design scored the lowest—as demonstrated by studies earning only 52% of the possible quality score in this category, on average—likely due to the over-representation of observational cross-sectional studies. The category describing exposure showed studies receiving the highest scores (78% of the possible quality score earned, on average), as explained by good temporal alignments between measurements of exposures and health outcomes. The two categories describing confounding and analyses faired in between the two above (66% and 61% of the possible quality score was earned, respectively). For example, most studies controlled adequately for potential confounders of the green space-health association (e.g., SES and age), but some did not justify the control variables included in the final models (see Table S5).

Collectively, the quality of evidence for differences in protective effects by SES was moderate, given the relatively modest strength of the effect (see EM mean value in Section 4.3) and that the 85 studies that tested for this relationship earned only 63% of the possible quality points. The quality of evidence for differences in protective effects by race/ethnicity was indeterminate. Relatively few studies (*n* = 25) tested for this relationship, and despite earning moderate scores for the bias evaluation (66% of the possible quality points), the findings displayed too many null results to classify the evidence as slight or moderate.

### 3.3. More Protective Effects for Whom?

**Figure 3:**
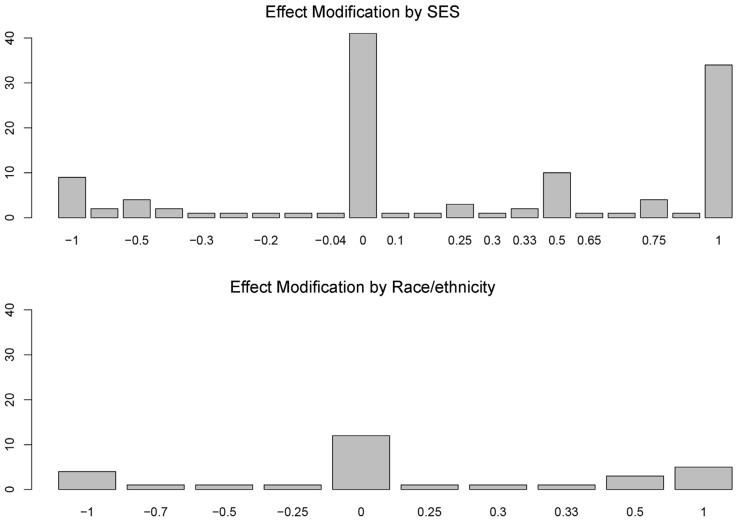
Bar charts showing the distributions of EM by SES and race/ethnicity. Scores of −1 indicate stronger protective effects for privileged populations (high-SES people or white people). Scores of 0 indicate no differences in protective effects between more and less disadvantaged populations. Scores of 1 indicate stronger protective effects for disadvantaged populations (low-SES people or racially/ethnically minoritized people). Comparisons between the two bar charts show that green space appears to exhibit stronger protective effects for low-SES people than for high-SES people, whereas green space does not show clear differences in protective effects by race/ethnicity.

Results showed that green space had more protective effects for low-SES groups than for high-SES groups, as the mean of the EM values for SES was 0.263 (SD = 0.619). The positive sign highlights that, across the reviewed studies, green space had more often stronger protective effects for low-SES groups than for high-SES groups. Figure 3 shows the frequency of EM values for both SES and race/ethnicity. The quality scores of analyses finding stronger protective effects for low-SES people, no differences, or stronger protective effects for high-SES people were comparable (62%, 63%, and 64% of the total possible quality points, respectively; see Table S5). Results for race/ethnicity showed no notable differences between White and racially/ethnically minoritized people, as the mean of the EM values was 0.064 (SD = 0.598; see Figure 3). The two sensitivity analyses (EM recoded as −1, 0, and 1, and nature-based programs removed from the sample) confirmed the results of the main analysis (see Table S7 and Figure S2 for the first sensitivity analysis; and Table S9 for the second). Thus, we found partial support for H1 (for SES but not for race/ethnicity).

The analysis of the seven articles reporting null/negative findings showed no evidence of effect modification (see Table S12). These findings confirm the robustness of our main results regarding green space having more protective effects for low-SES people than for affluent people.

### 3.4. Does Green Space Type Matter?

A Kruskal–Wallis test to compare mean ranks of EM value for SES by green space type showed marginally significant results (χ^2^(3) = 7.492, *p* = 0.057). Dunn’s post-hoc pairwise tests showed that public green space was significantly more likely to have stronger protective effects for low-SES people than green land cover, *p* = 0.012 (see Table S6). For context, the quality scores of analyses focusing on public green space and green land cover were relatively similar (60% and 64%, respectively). A Kruskal–Wallis test did not highlight differences in EM values for race/ethnicity based on green space type (χ^2^(2) = 2.926, *p* = 0.231). Thus, we found no support for H2, as our results for SES showed just the opposite: Public green spaces displayed stronger protective effects for low-SES people than green land cover.

We also tested whether EM values for SES and race/ethnicity varied by distance from green space, for a subsample of studies that used a fixed threshold to measure green space exposure (74 rows). The Kruskal–Wallis test for EM values for SES was significant (χ^2^(3) = 21.051, *p* < 0.001), whereas the same test for the race/ethnicity EM values was not (χ^2^(2) = 4.250, *p* = 0.119). Dunn’s post-hoc pairwise tests for SES showed that green space distance ranges of 501–1000 m, 1001–2000 m, and larger than 2000 m had more protective effects for low-SES versus high-SES people than the 0–500 m range (all *p* < 0.05, Table S6). The quality scores of analyses that considered different distance thresholds were similar for the 0–500 m and 501–1000 m ranges (64% and 63%, respectively), but lower for the two highest thresholds (59% for 1001–2000 m, and 55% for 2000+ m). The two sensitivity analyses generally confirmed the findings for green space type and distance (see Table S8 and Figure S3 for the first sensitivity analysis; and Tables S10 and S11 for the second).

Means for the EM values of different green space types and green space distances are reported in Table 3 for illustrative purposes. Although tests for the EM values by race/ethnicity were not significant, the descriptive statistics in Table 3 showed similar trends as those for SES: public green space had a larger mean than green land cover (i.e., public green space had more protective effects for racially/ethnically minoritized people than green land cover), and larger green space distances (e.g., 1001–2000 m) had higher means than the smallest (i.e., 0–500 m).

### 3.5. Does the Type of Health Outcome Matter?

A Kruskal-–Wallis test to compare mean ranks of EM value for SES by type of health outcomes was not significant (χ^2^(7) = 8.306, *p* = 0.306). Yet Dunn’s post-hoc pairwise tests showed that green space had stronger beneficial effects for low-SES people (as compared to high-SES people) for general health than birth outcomes, *p* = 0.010, and for cardiovascular health/disease than for birth outcomes, *p* = 0.065 (Table S6). The first sensitivity analyses (EM values recoded as −1, 0, or 1) for SES showed diverging results from the main analysis, as neither of the significant pairwise comparisons in the main analysis remained so when recoding (see Table S8 and Figure S7). The second sensitivity analysis (nature-based programs removed) highlighted similar findings to those of the main analysis (see Tables S10 and S11).

A Kruskal–Wallis test did not highlight differences in EM values for race/ethnicity based on the health outcome type (χ^2^(4) = 2.857, *p* = 0.581). Yet the means shown in Table 3 suggest that green space might have stronger protective effects for racially/ethnically minoritized (as compared to white people) for cardiovascular health/disease (mean = 0.214, SD = 0.755, *n* = 7).

### 3.6. Does the Continent Matter?

A Kruskal–Wallis test to compare mean ranks of EM value for SES by continent was significant (χ^2^(4) = 15.836, *p* = 0.003). Dunn’s post-hoc pairwise tests showed that green space had greater health benefits for low-SES people (as compared to high-SES people) in Europe than in North America, *p* < 0.001 (Table S6). The two sets of sensitivity analyses confirmed this result (see Table S8 and Figure S9 for the first sensitivity analysis; and Tables S10 and S11 for the second). The quality scores of analyses focusing on Europe and North America were relatively similar (62% and 65%, respectively).

The Kruskal–Wallis test for race/ethnicity was not significant (χ^2^(2) = 4.467, *p* = 0.107), but two Dunn’s post-hoc pairwise comparisons were marginally significant: green space had stronger beneficial associations with health for racially/ethnically minoritized people in Europe as opposed to North America, *p* = 0.056, and in Oceania as opposed to North America, *p* = 0.074 (Table S6). The first sensitivity analysis for these tests (EM values recoded as −1, 0, or 1) showed non-significant results (see Table S8), but the second (nature-based programs removed) highlighted consistent results to those of the main analysis (see Tables S10 and S11).

## 4. Conclusions

### 4.1. Summary of Findings

In this systematic review, we examined whether green space shows greater protective effects for the physical health of disadvantaged or privileged groups. By doing so, we aimed to gather evidence about whether green space can contribute to limiting health disparities and moving toward health equity. We analyzed 90 peer-reviewed articles that reported whether SES and/or race/ethnicity modified the association between green space and physical health outcomes. Fewer articles studied effect modification by race/ethnicity than by SES. Additionally, most studies that did focus on race/ethnicity were set in the U.S., where, due to systematic racism, race and ethnicity are important determinants of health outcomes [1]. The moderate quality of evidence scores for SES that emerged from our analysis of methodological bias makes us reasonably confident about these particular results, which are presented below. Yet the most significant methodological limitation affecting these studies was the frequent use of observational cross-sectional designs, which highlights the need for more research using longitudinal, experimental, or quasi-experimental designs. Several other recent reviews on green space and health highlighted a similar limitation among their included studies [19,20,83,173]. The indeterminate quality of evidence for race/ethnicity signals that these results should be interpreted with caution and emphasizes the need for future attempts to synthesize and add to the available evidence.

We found promising evidence for efforts to achieve health equity. As expected (H1), green space had greater protective effects for low-SES people and neighborhoods than for more affluent groups (Research Question 1). These findings are reinforced by two recent studies with similar conclusions that showed green space provides more health benefits in countries with lower income than in more affluent countries, likely because the latter have better medical service and more services to improve health than less wealthy countries [174,175]. We did not find noticeable differences in the protective effects of green space between white and racially/ethnically minoritized people, yet the relatively small sample of articles reporting effect modification by race/ethnicity (*n* = 24) warrants more research on this topic.

Our results also showed that public green space (e.g., parks) had stronger protective effects for low-SES groups (as opposed to high-SES groups) than measures of green land cover (e.g., greenness; Research Question 2). This is the opposite of what we expected (H2), since the poor quality of parks in disadvantaged areas, at least in the U.S. and Global South countries [32,33], should limit the protective effects of parks for disadvantaged groups. To our knowledge, fewer studies have found inequities in park quality in other contexts, such as Europe (e.g., [176,177]). Our unexpected findings may be explained by most parks being free and accessible to the public; they might be the only place for exercise for low-SES people who cannot easily afford private recreation options such as gyms [44,45]. Further, parks might serve as spaces for socialization and social well-being [178], which in turn might influence physical health [179]. Latinx and Black people in the U.S.—who are more likely to have lower SES than white people—use parks in groups for social activities more than white people [180,181]. Parks have also been identified by minoritized populations outside of the U.S. as determinants of health more so than other types of urban green spaces such as green streets [182].

Additionally, we found that when larger distances around one’s home were considered, green space showed stronger protective effects for low-SES people than for more affluent groups. This might be in part because the benefits of green space are more consistently detected when measuring green spaces in a broad buffer from one’s home (e.g., up to 2000 m) than in a very small buffer (e.g., less than 400 m) [183]. Broader neighborhood contexts may better estimate the activity spaces in which some forms of exposure that result in health outcomes, like physical activity and socialization, than narrower contexts [71]. Further, low-SES people and racially/ethnically minoritized people might walk longer distances and times than privileged groups, in part due to limited access to private vehicles [184], and therefore show beneficial associations with green space at greater distances than other populations. Thus, researchers might need to consider the broad neighborhood context when measuring green space exposure for low-SES people to accurately capture the protective effects of exposure.

Tests to examine whether the type of health outcome matters in whether disadvantaged groups benefit more from green space showed inconsistent results (Research Question 3). Although pairwise tests highlighted that that green space has stronger protective effects for low-SES people (as compared to high-SES people) for general health and cardiovascular health/disease than for birth outcomes, the first sensitivity analysis (EM values recoded as -1, 0, and 1) did not show the same differences. It should also be noted that because we included eight types of health outcomes, many comparisons were between pairs with few cases, which limited statistical power.

Finally, we found strong differences by continent. Green space had stronger protective effects for low-SES people than high-SES people in studies focusing on Europe as opposed to North America (mostly representing studies in the U.S.). These findings might be because green space has higher quality in Europe’s low-SES neighborhoods than in North America’s disadvantaged neighborhoods. Indeed, among the studies in Rigolon’s review [32], more than a dozen found inequities in park quality in the U.S., whereas only two found such inequities in European contexts (e.g., [176,177]). Additionally, Schüle et al.’s [34] recent review of studies in Europe does not shed light on whether inequities in green space quality exist in that continent. Further, most European countries have a welfare state form of government, which provides more public services for disadvantaged populations (e.g., universal health care) than the U.S. government [185]. Additionally, low-SES people of color in the U.S. face systemic racism and police violence; with evidence of police harassment and discrimination of low-SES people of color in parks [186,187], those individuals might choose to avoid parks. Europe’s public health care systems might also push countries to invest in upstream health interventions such as parks, especially in low-SES areas where people with poor health status reside [188,189].

Similar findings emerged for effect modification by race/ethnicity regarding green space type (Research Question 2) and continent (Research Question 4), although they were not statistically significant likely due to the small sample size. Green space had stronger protective effects for racially/ethnically minoritized people than white people when analyses focused on public green space (as opposed to green land cover) and focused on Europe (as opposed to North America).

### 4.2. Strengths, Limitations, and Future Research

The main strength of this review is its focus on SES and race/ethnicity as modifiers of the green space–human health relationship. To the best of our knowledge, this is the first systematic review that analyzed quantitatively whether green space can help advance health equity goals. Another strength of the review is its inclusion of eight types of physical health outcomes for which health disparities exist. Since we did not include mental health outcomes, future reviews could examine whether disadvantaged or privileged groups show stronger associations between green space and greater mental health [190]. An additional strength of this review is its global focus, as we included studies from five continents and uncover significant differences between findings in Europe and North America. Thus, when also considering our large sample of studies with effect modification by SES (*n* = 85), our findings might apply to low-SES people in a variety of contexts.

Regarding limitations, we decided to create categories of health outcomes and green space to make inferential statistics possible rather than using the exact measures of health and exposure described in each article (for example, we classified tree canopy cover as green land cover). This choice might have resulted in a loss of detail regarding measures of green space and health. Yet, had we entered each individual measure for each green space and each health outcome, we would have had too many categories to draw meaningful conclusions about green space types and health outcome types. We also studied broad disease categories with diverse markers of disease. For example, the effect of green space on atopic diseases may differ across its broad measurements (i.e., allergies, asthma, and respiratory infection/disease/function/mortality) [191,192,193,194]. Our sample of papers within this disease category was too small to examine specific health endpoints. Our aggregated findings should be considered preliminary evidence rather than conclusive findings for these myriad aspects of respiratory health.

Relatedly, we were unable to pool estimates from individual papers and conduct formal meta-analyses due to the variability in reporting—and commonly the underreporting—the details of effect modification tests. Instead, our findings were calculated from our best attempt to systematically code the myriad ways to report the findings from these tests. As indicated in our moderate inter-rater agreement scores, this process was characterized by difficulty interpreting the directionality and statistical significance of many papers’ findings. Our results may have differed if we had weighted articles’ contributions by sample sizes and variance, for instance, as would have been accomplished with a formal meta-analysis had that option been available to us. Similar to the above discussion, we categorized studies based on continents, which albeit having some common sociopolitical characteristics and similarities regarding associations between green space and human health [175], include a range of diverse countries. Thus, our findings regarding continents mostly speak about differences between the United States (over-represented in the North American sample) and European countries.

Further, most of the studies we identified through our search were cross-sectional, and therefore more research is needed to ascertain whether green space causes greater protective effects for low-SES people as opposed to high-SES people. Alternative explanations for our findings include residential self-selection bias, which describes the possibility of people seeking healthier lifestyles choosing to live in areas that facilitate those lifestyles, such as green and walkable neighborhoods [195,196,197], and structural forces, such as racist attitudes in the housing market, which limit where disadvantaged people can live [198]. Finally, our sample of studies focusing on effect modification by race/ethnicity is relatively small, which makes the generalizability of those specific findings limited. In particular, only one study among the 90 included focused on indigenous people [114].

Our analysis also highlights the need for additional research on whether measures of disadvantage modify the green space–health associations. First, more studies are needed to understand whether race or ethnicity act as effect modifiers in the relationship between green space and health. Specifically, more work is needed in Europe, which is becoming more racially and ethnically diverse due to in-migration and refugee resettlement [199]. Second, more research could be conducted in Global South countries, most of which experience significant wealth inequalities [200]. Third, more studies that evaluate the health impacts of similar green space initiatives (e.g., tree planting programs) in disadvantaged and privileged communities are needed. Fourth, more research should examine whether, in socially-mixed neighborhoods such as gentrifying communities, green space-health associations are stronger for low-SES or high-SES people [201].

### 4.3. Policy Implications

Despite the limitations of this review and of the included studies, our results may have implications for public policies and initiatives on public health and green space. First, our main results suggest that green space could be used as a tool to promote health equity. Although several public agencies and nongovernmental organizations (NGOs) around the world have created and activated green space in low-SES communities to improve health outcomes among those populations [38,59,202], the results of this review provide them with further evidence to advocate for more funding supporting green space in such communities.

Second, our results suggest that organizations working to achieve health equity may choose to prioritize public green space (e.g., parks) in low-SES communities over other types of green space (e.g., street trees). In this regard, several environmental justice NGOs in cities around the world have partnered with public agencies to build, maintain, and activate parks in disadvantaged communities [203,204,205]. Collaborations between health care providers and parks organizations are also emerging, as shown by the growth of park prescription (or ParkRx) programs, some of which have focused on disadvantaged populations [59,206]. Our findings also suggest that planners should consider the provision of green space for low-SES residents beyond the immediate surroundings of their homes (0–500 m) and look instead at thresholds up to 2 km.

Third, our finding that green space had stronger protective effects for low-SES people (and to some extent racially/ethnically minoritized people) in Europe than in North America shows that context matters. In other words, health equity organizations might consider broader societal contexts such as access to health care, the geography of cities, and systemic racism when planning green space initiatives to address health disparities [2,12,13]. Our finding that context matters also suggests that green space is only one piece of the puzzle to achieve health equity, one that needs to be integrated with initiatives to provide access to housing and health care to disadvantaged groups and to remove hazardous exposures in their neighborhoods [1,2,12]. Ultimately, we hope that our findings will stimulate more research and policy initiatives on how green space can be integrated with other interventions to move the needle toward health equity.

## Figures and Tables

**Table 1:** Summary of health disparities based on socioeconomic status and race/ethnicity.

	Low SES	Racially/Ethnically Minoritized Group	
*Disparity category*	*Source*	*Source*	*Minoritized group affected*
Life expectancy	[3,4,10,53,60]	[4,8,10,60]	Black people (U.S.), Indigenous people (Australia)
All-cause mortality	[11,51]	[2,11]	Native American people (U.S.), Black and Indian people (South Africa)
Homicide-related deaths	[2]	[2]	Black and Latinx people (U.S.)
Number of chronic conditions	[3,8,10]	[8,10,61]	Black and two-race people (U.S.), Indigenous people (Australia), immigrants (Germany, Spain, Sweden, Switzerland)
Cardiovascular health/disease	[2,4,8,10]	[2,4]	Black people (U.S.)
Cancer	[2,10]		
Obesity or overweight	[2,3,4,8,10]	[4,8,61,62]	Black and Latinx people (U.S.), non-White people (The Netherlands), immigrants (Germany, Sweden)
Diabetes	[2,4,8,10]	[4,8,62]	Black, Asian, Latinx, and Native American people (U.S.), non-White people (The Netherlands)
Atopic diseases (e.g., asthma, eczema)	[8]	[8]	Black and Native American people (U.S.)
Poor self-reported health	[4,7,8,51,53]	[4,8,9,10,61,63]	Black, Native American, and Latinx people (U.S.), Indigenous people (Australia), non-White people (United Kingdom), immigrants (France, Germany, The Netherlands, Sweden, Switzerland), ethnically minoritized people as opposed to Han Chinese (China)
Preterm births	[64]	[2,4,8]	Black people (U.S.)
Infant mortality	[4]	[2,65]	Black people (U.S.), Black people (South Africa)

Notes: Disparities describe a condition in which a disadvantaged group (e.g., low-SES people) experience worse health outcomes than privileged people (e.g., wealthier people).

**Table 2:** Articles included in the review

Authors and Date	EM Focus	Green Space Type (s)	Health Outcome Type (s)	Continent
Abelt and McLafferty (2017) [84]	SES	Green land cover	Birth outcomes	North America
Agay-Shay et al. (2014) [85]	SES	Green land cover	Birth outcomes	Europe
Agay-Shay et al. (2019) [86]	SES	Green land cover	Birth outcomes	Europe
Agyemang et al. (2017) [87]	Race	Public green space	CVD	Europe
Alexander et al. (2013) [88]	Race	Public green space	Obesity-related	North America
Astell-Burt et al. (2014) [89]	SES	Public green space	Diabetes	Oceania
Björk et al. (2008) [90]	SES	Green land cover	General health, Obesity-related	Europe
Brindley et al. (2018) [91]	SES	Gardens	General health	Europe
Brown et al. (2016) [92]	SES	Green land cover	CVD, Diabetes, General health	North America
Browning and Rigolon (2018) [93]	SES, Race	Green land cover	Obesity-related	North America
Casey et al. (2016) [94]	SES	Green land cover	Birth outcomes	North America
Coppel and Wüstemann (2017) [95]	SES	Public green space	General health	Europe
Crouse et al. (2017) [96]	SES	Green land cover	Mortality	North America
Cummins and Fagg (2012) [97]	SES	Public green space	CVD	Europe
Cusack et al. (2017a) [98]	SES, Race	Green land cover	Birth outcomes	North America
Cusack et al. (2017b) [99]	SES, Race	Green land cover	Birth outcomes	North America
Cusack et al. (2018) [100]	SES, Race	Green land cover	Birth outcomes	North America
da Silveira and Junger (2018) [101]	SES	Green land cover	CVD	South America
Dadvand et al. (2012a) [102]	SES	Green land cover, Public green space	Birth outcomes	Europe
Dadvand et al. (2012b) [103]	SES	Green land cover	Birth outcomes	Europe
Dadvand et al. (2014a) [104]	SES	Green land cover, Public green space	Atopic disease, Obesity-related	Europe
Dadvand et al. (2014b) [105]	SES, Race	Green land cover	Birth outcomes	Europe
Dadvand et al. (2018) [106]	SES	Public green space	Diabetes	Asia
D’Agostino et al. (2018a) [107]	SES	Nature-based programs	CVD, Obesity-related	North America
D’Agostino et al. (2018b) [108]	SES	Nature-based programs	CVD, Obesity-related	North America
Dalton et al. (2016) [109]	SES	Green land cover	Diabetes	Europe
de Keijzer et al. (2017) [110]	SES	Green land cover	Mortality	Europe
de Vries et al. (2003) [111]	SES	Green land cover, Gardens	General health	Europe
Demoury et al. (2017) [112]	SES	Green land cover	Cancer	North America
Donovan et al. (2013) [113]	SES	Green land cover	Atopic disease, CVD	North America
Donovan et al. (2018) [114]	SES, Race	Green land cover	Atopic disease	Oceania
Dzhambov et al. (2018) [115]	SES	Green land cover	CVD	Europe
Ebisu et al. (2016) [116]	SES, Race	Green land cover	Birth outcomes	North America
Egorov et al. (2017) [117]	SES, Race	Green land cover	CVD	North America
Eldeirawi et al. (2019) [118]	SES	Green land cover	Atopic disease	North America
Fan and Jin (2014) [119]	SES, Race	Public green space	Obesity-related	North America
Fong et al. (2018) [120]	SES, Race	Green land cover	Birth outcomes	North America
Foster and Weinstein (2019) [121]	SES	Public green space	Obesity-related	North America
Gidlow et al. (2016) [122]	SES	Green land cover	Mortality	Europe
Glazer et al. (2018) [123]	SES, Race	Green land cover	Birth outcomes	North America
Groenewegen et al. (2018) [124]	SES	Green land cover	Atopic disease, CVD, Diabetes, General health	Europe
Hobbs et al. (2017) [125]	SES	Public green space	Obesity-related	Europe
Hobbs et al. (2018) [126]	SES	Public green space	Obesity-related	Europe
Hughey et al. (2017) [127]	SES, Race	Public green space	Obesity-related	North America
Hystad et al. (2014) [128]	SES, Race	Green land cover	Birth outcomes	North America
James et al. (2016) [129]	SES, Race	Green land cover	Mortality	North America
Ji et al. (2019) [130]	SES	Green land cover	Mortality	Asia
Jilcott Pitts et al. (2013) [131]	SES	Public green space	Obesity-related	North America
Kihal-Talantikite et al. (2013) [132]	SES	Public green space	Birth outcomes	Europe
Kling et al. (2018) [133]	Race	Nature-based program	CVD, Obesity-related	North America
Lachowycz and Jones (2014) [134]	SES	Public green space	CVD	Europe
Lovasi et al. (2012) [135]	SES	Green land cover	Obesity-related	North America
Maas et al. (2006) [136]	SES	Green land cover	General health	Europe
Maas et al. (2006) [137]	SES	Public green space	Atopic disease, CVD, Diabetes, General health,	Europe
Markevych et al. (2014) [138]	SES	Green land cover	Birth outcomes	Europe
Mena et al. (2015) [139]	SES	Public green space	Obesity-related	South America
Mitchell and Popham (2018) [140]	SES	Public green space	CVD, Mortality	Europe
Mueller et al. (2018) [141]	SES, Race	Public green space	Mortality	Europe
Nichani et al. (2017) [142]	SES, Race	Green land cover	Birth outcomes	Oceania
Nieuwenhuijsen et al. (2018) [143]	SES	Green land cover	Mortality	Europe
Orioli et al. (2019) [144]	SES	Green land cover	Atopic disease, CVD, Mortality	Europe
Persson et al. (2018) [145]	SES	Green land cover	Obesity-related	Europe
Petraviciene et al. (2018) [146]	SES	Green land cover	Obesity-related	Europe
Pun et al. (2018) [147]	SES, Race	Green land cover	CVD	North America
Reid et al. (2017) [148]	SES	Green land cover	General health	North America
Richardson et al. (2018) [149]	SES	Green land cover	Birth outcomes	Europe
Rossi et al. (2018) [150]	SES	Public green space	Obesity-related	South America
Rossi et al. (2019) [151]	SES	Public green space	Obesity-related	South America
Ruijsbroek et al. (2017) [42]	SES	Public green space	General health	Europe
Sarkar (2017) [152]	SES	Green land cover	Obesity-related	Europe
Schalkwijk et al. (2018) [153]	SES	Gardens, Public green space	Obesity-related	Europe
Schuler and O’Reilly (2017) [154]	SES	Public green space	Obesity-related	North America
Seo et al. (2019) [155]	SES	Public green space	CVD	Asia
Singh et al. (2010) [156]	SES, Race	Public green space	Obesity-related	North America
Sullivan et al. (2014) [157]	Race	Public green space	Obesity-related	North America
Thiering et al. (2016) [158]	SES	Green land cover	Diabetes	Europe
Triguero-Mas et al. (2015) [159]	SES	Green land cover, Public green space	General health	Europe
Van Der Zwaard et al. (2018) [160]	SES	Gardens	Obesity-related	Europe
Vienneau et al. (2017) [161]	SES	Green land cover	Atopic disease, CVD, Mortality,	Europe
Villeneuve et al. (2012) [162]	SES	Green land cover	Atopic disease, Mortality	North America
Villeneuve et al. (2018) [163]	SES	Green land cover	Obesity-related	North America
Wang and Lan (2019) [164]	SES	Public green space	Atopic disease, Obesity-related	Asia
Wen and Kowaleski-Jones (2012) [165]	Race	Public green space	Obesity-related	North America
Wilker et al. (2014) [166]	SES	Green land cover	CVD	North America
Wu et al. (2018) [167]		Green land cover, Public green space	CVD	North America
Xu et al. (2017) [168]	SES	Green land cover	Atopic disease, CVD, Diabetes	Asia
Yang et al. (2019a) [169]	SES	Green land cover	CVD	Asia
Yang et al. (2019b) [170]	SES	Green land cover	Diabetes	Asia
Yeager et al. (2018) [171]	SES, Race	Green land cover	CVD	North America
Yitshak-Sade et al. (2019) [172]	SES, Race	Green land cover	CVD	North America

Notes: EM = effect modification, SES = socioeconomic status, CVD = cardiovascular health/disease.

**Table 3:** Means for EM values classified by green space type, green space distance, health outcome type, and continent.

	EM SES		EM Race/Ethnicity	
	Mean (SD)	*n*	Mean (SD)	*n*
Green space type				
Gardens	0.500 (0.577)	4		
Green land cover	0.154 (0.594)	78	−0.007 (0.644)	20
Nature-based program	0.500 (0.577)	3	0.500 (0.000)	2
Public green space	0.447 (0.585)	36	0.135 (0.570)	8
Green space distance				
0–500 m	−0.009 (0.529)	52	−0.060 (0.584)	15
501–1000m	0.518 (0.464)	14	1.000 (0.000)	1
1001–2000 m	0.750 (0.500)	4	1.000 (0.000)	2
>2000 m	1.000 (0.000)	4		
Health outcome type				
Atopic disease	0.273 (0.606)	11	−0.250 (0.000)	1
Birth outcomes	0.072 (0.466)	22	−0.036 (0.506)	11
Cancer	0.000 (0.000)	2		
Cardiovascular health/disease	0.218 (0.596)	25	0.214 (0.755)	7
Diabetes	0.425 (0.472)	10		
General health	0.559 (0.519)	17		
Mortality	0.091 (0.943)	11	0.500 (0.707)	2
Obesity-related	0.305 (0.613)	24	0.009 (0.653)	9
Continent				
Asia	0.158 (0.609)	10		
Europe	0.448 (0.564)	61	0.625 (0.478)	4
North America	0.022 (0.559)	44	−0.013 (0.607)	24
Oceania	0.416 (0.381)	3	−0.125 (0.176)	2
South America	0.250 (0.957)	4		

Note: SD represents standard deviation. *n* represents the number of rows in which the effect modification was tested. The corresponding descriptive statistics for the first sensitivity analysis are in Figures S3–S10 and those for the second are in Table S9.

## Data Availability

Data available upon request.

## Supplementary Materials

The following are available online at https://www.mdpi.com/1660-4601/18/5/2563/s1, Table S1. Databases used in previous reviews on green space and health and on access to green space. Table S2. Search terms used in reviews on green space and health and on access to green space. Table S3. Search expressions used in this review. Table S4. Codes included in the data extraction sheet. Table S5. Points assigned to each bias category for the articles included in the review. Table S6. Dunn’s post-hoc pairwise tests for the Effect Modification (EM) values. Table S7. First Sensitivity analysis: Summary of statistical tests conducted. EM values were recoded as integers (−1, 0, or 1). Table S8. First Sensitivity analysis: Post-hoc pairwise comparisons for Fisher’s exact tests for the Effect Modification (EM) values. EM values were recoded as integers (−1, 0, or 1). Table S9. Second sensitivity analysis (exclude nature-based programs): Means for EM values classified by green space type, green space distance, health outcome type, and continent. Table S10. Second sensitivity analysis (exclude nature-based programs): Kruskal-Wallis tests for the Effect Modification (EM) values. Table S11. Second sensitivity analysis (exclude nature-based programs): Dunn’s post-hoc pairwise tests for the Effect Modification (EM) values. Table S12. Articles in which (a) the green space-health relationship was null or negative and (b) reported effect modification tests for such relationship. Table S13. PRISMA Checklist. Figure S1. Year of publication of the 90 articles included in this review. Figure S2. First sensitivity analysis: Effect modification (EM) for SES and Race/ethnicity. EM was recoded as −1, 0, or 1. Figure S3. First sensitivity analysis: Effect modification (EM) for SES classified by green space type. EM was recoded as −1 (high-SES people benefit more), 0 (no differences), or 1 (low-SES people benefit more). Figure S4. First sensitivity analysis: Effect modification (EM) for race/ethnicity classified by green space type. EM was recoded as −1 (White people benefit more), 0 (no differences), or 1 (non-White people benefit more). Figure S5. First sensitivity analysis: Effect modification (EM) for SES classified by green space distance. EM was recoded as −1 (high-SES people benefit more), 0 (no differences), or 1 (low-SES people benefit more). Figure S6. First sensitivity analysis: Effect modification (EM) for race/ethnicity classified by green space distance. EM was recoded as −1 (White people benefit more), 0 (no differences), or 1 (non-White people benefit more). Figure S7. First sensitivity analysis: Effect modification (EM) for SES classified by health outcome type. EM was recoded as −1 (high-SES people benefit more), 0 (no differences), or 1 (low-SES people benefit more). CVD: Cardiovascular health/disease. Gen health: General health. Figure S8. First sensitivity analysis: Effect modification (EM) for race/ethnicity classified by health outcome type. EM was recoded as −1 (White people benefit more), 0 (no differences), or 1 (non-White people benefit more). Figure S9. First sensitivity analysis: Effect modification (EM) for SES classified by continent. EM was recoded as −1 (high-SES people benefit more), 0 (no differences), or 1 (low-SES people benefit more). Figure S10. First sensitivity analysis: Effect modification (EM) for race/ethnicity classified by continent. EM was recoded as −1 (White people benefit more), 0 (no differences), or 1 (non-White people benefit more).
