# Supplementary material for: Green Space and Health Equity: A Systematic Review on the Potential of Green Space to Reduce Health Disparities
Source: Int J Environ Res Public Health. 2021 Mar 4;18(5):2563. doi: 10.3390/ijerph18052563 (PMC7967323; doi:10.3390/ijerph18052563)
Supplement: Supplementary file 1 [file ijerph-18-02563-s001.pdf]

Green space and health equity: A systematic review on the potential of green space to reduce health disparities

Supplementary Materials

Methodological Details

2.1. Article Search and Data Extraction

2.1.1. Focus on Green Space

We focused on green space and excluded other natural environments such as 'blue space' (water), 'white space' (salt), 'yellow space' (sand), 'brown space' (desert), despite mounting evidence suggesting these environments protect health (Garrett, Clitherow, White, Wheeler, & Fleming, 2019; Garrett, White, et al., 2019; K. Wang, Cui, & Xu, 2018; Zając, Bojar, Helbin, Kolarzyk, & Owoc, 2014). Unlike green space, which is seeing a surge of interest as a nature-based preventative health intervention through forest bathing (Y. Wen, Yan, Pan, Gu, & Liu, 2019) and park prescriptions (Labib, Shuvo, Browning, & Rigolon, 2020; Razani et al., 2019), non-green natural environments are witnessing less adoption in clinical practice in most areas of the world and are likely to require substantially more engineering/maintenance costs than greening interventions. In particular, increasing the possibility of exposure to blue spaces could involve daylighting (unearthing) buried streams, damming streams and rivers, digging out lakes, or shifting coastlines – all of which would be tremendously costlier as a public health intervention than planting street trees or transforming a vacant lot into a green pocket park. Therefore, we believed the likelihood that non-green natural environments would be used to overcome health disparities in the near future is relatively low. Furthermore, because few empirical research articles analyzed the health benefits of non-green natural environments (Gascon, Zijlema, Vert, White, & Nieuwenhuijsen, 2017), we expected the number of such articles examining differences by SES and race/ethnicity to be too little for sufficiently-powered comparisons. Last, human-made environmental exposures related to natural environments such as herbicides (Schreinemachers, 2003) were also excluded because we focused on whether the beneficial effects of green space differed by level of disadvantage, rather than whether harmful environmental exposures differed by level of disadvantage, which has also been studied extensively (e.g., Gelormino et al., 2015; Robinson et al., 2018).

2.1.2. Databases and Search Expressions

To identify the academic databases to use for our literature search, we compiled a list of databases used in recent systematic reviews on the benefits of green space for human health (e.g., Browning & Lee, 2017; Gascon et al., 2016; Twohig-Bennett & Jones, 2018; van den Bosch & Ode Sang, 2017), as well as reviews on access to green space (e.g., Rigolon, 2016; Rigolon et al., 2018; Schüle et al., 2019) (see Table S1). We chose five databases that were among the most used in the aforementioned reviews, were available through our university libraries, and provided disciplinary diversity (from public health to geography). As such, we selected three health-focused databases – CINAHL, Cochrane, and PubMed (which includes MedLine) – and two databases covering several social science disciplines – Scopus and Web of Science.

To develop our search expressions, we examined the keywords used in previous systematic reviews on green space and health as well as access to green space (see Table S2). For each construct, which included green space and each one of the physical health outcomes, we used a variety of synonyms that were included as search terms in the aforementioned reviews. We did *not* include constructs related to structural inequalities because some papers report effect modifications without mentioning inequalities in the title, keyword, or abstract. Search expressions differed based on the database, as each uses a different syntax (see Table S3). We also trialed the search terms to ensure notable papers on effect modification by SES/race-ethnicity in the green space and health relationship were identified.

2.1.3. Entering New Rows in the Dataset

We entered multiple rows in the spreadsheet for the same article if the article included more than one of the following characteristics: (1) research design (i.e., cross-sectional or longitudinal); (2) health outcome type; (3) green space type; (4) distance between the study's unit of analysis and green space; (5) green space measurement, including objective (e.g., GIS) or subjective evaluations; or (6) cities or countries where data were collected, if results were reported for separate samples. For example, an article in which effect modification by SES or race/ethnicity was studied for two green space types received two rows. This approach

allowed us to capture multiple results from each study and was instrumental to answer research questions 2 (green space type) and 3 (health outcome type).

2.1.4. Coding of Effect Modification Results

Some articles reported both split-sample and interaction tests of effect modification for the same row of data (e.g., associations between public green space and diabetes). In these cases, we averaged the values of the effect modification codes that applied to each effect modification analysis. For example, we entered 0.5 if the split-sample analysis received a value of 1.0 for showing that green space had protective effects for low-SES people and not for high-SES people but the SES*green space interaction term was assigned a value of 0.0 since it was not significant.

We also averaged EM values when articles reported several effect modification tests for the same health outcome type (e.g., both BMI and waist circumference for obesity-related measures) or multiple effect modification tests for the same green space type (e.g., both park proximity and park acreage for public green space). As a result of averaging, effect modification values can assume any decimal value between -1.00 and 1.00 (e.g., -0.50, 0.33). The process of assigning values for the effect modification in each row resulted in a good inter-rater agreement (82% for EM SES and 65% for EM race/ethnicity).

We also averaged EM values when articles reported several effect modification tests for the same health outcome type (e.g., both BMI and waist circumference for obesity-related measures) or multiple effect modification tests for the same green space type (e.g., both park proximity and park acreage for public green space). This approach allows us to represent a summary measure of effect modification for each row. In the above cases, we gave equal weight to each type of effect modification analysis, health outcome, and green space measure since either approach (multiplicative interaction terms and split samples) can yield equally valid results depending on the study (Iacobucci, Posavac, Kardes, Schneider, & Popovich, 2015).

Two researchers carried on the above processes independently, including adding rows to the spreadsheet and entering codes for each row (e.g., values for effect modification). The few divergent rows and codes were discussed and agreed upon.

2.1.5. Analysis of Methodological Bias

Bias Evaluation Instrument. Building on Radke et al.'s (2019) work, we developed a bias evaluation instrument and subsequent quality of evidence scale including four categories that are relevant for the green space-health connection: (1) study design, (2) green space exposure, (3) use and justification of confounders, and (4) statistical analysis (see Table S4 for more details). For each category, we focused on elements that other reviews on the benefits of green space identified as methodological weaknesses (Dzhambov, Browning, Markevych, Hartig, & Lercher, 2020; Labib, Lindley, & Huck, 2020; Markevych et al., 2017). For example, we evaluated a study's statistical analysis by checking whether the authors reported model assumptions, potential spatial autocorrelation issues, and other aspects (see Table S4).

Like other reviews on green space and health (e.g., Twohigg-Bennet & Jones, 2018), we did not evaluate bias for the health outcomes. Specifically, objective versus subjective measures are of concern with bias, but here these differences were represented by the outcome measured; general health was solely subjectively measured (e.g., perceived health), whereas diseases and other health outcomes were largely objectively measured. For example, the latter included medical examinations (e.g., blood pressure, BMI), diagnoses (e.g., asthma, cancer), and birth and mortality records. Without sufficient variability in the type of measurement, we believed including this as a potential measure of bias itself would degrade one of our outcomes (general health) without additional insights.

References

Browning, M. H. E. M., & Lee, K. (2017). Within what distance does "greenness" best predict physical health? A systematic review of articles with gis buffer analyses across the lifespan. *International Journal of Environmental Research and Public Health*, *14*(7), 1–21. https://doi.org/10.3390/ijerph14070675

Dzhambov, A. M., Browning, M. H. E. M., Markevych, I., Hartig, T., & Lercher, P. (2020). Analytical approaches to testing pathways linking greenspace to health: A scoping review of the empirical literature. *Environmental Research*, *186*, 109613. https://doi.org/10.1016/j.envres.2020.109613

Garrett, J. K., Clitherow, T. J., White, M. P., Wheeler, B. W., & Fleming, L. E. (2019). Coastal proximity and mental health among urban adults in England: The moderating effect of household income. *Health and Place*, *59*, 102200. https://doi.org/10.1016/j.healthplace.2019.102200

Garrett, J. K., White, M. P., Huang, J., Ng, S., Hui, Z., Leung, C., … Wong, M. C. S. (2019). Urban blue space and health and wellbeing in Hong Kong: Results from a survey of older adults. *Health and Place, 55*, 100–110. https://doi.org/10.1016/j.healthplace.2018.11.003

Gascon, M., Triguero-Mas, M., Martínez, D., Dadvand, P., Rojas-Rueda, D., Plasència, A., & Nieuwenhuijsen, M. J. (2016). Residential green spaces and mortality: A systematic review. *Environment International*, *86*,

60–67. https://doi.org/10.1016/j.envint.2015.10.013
Gascon, M., Zijlema, W., Vert, C., White, M. P., & Nieuwenhuijsen, M. J. (2017). Outdoor blue spaces, human health and well-being: A systematic review of quantitative studies. *International Journal of Hygiene and Environmental Health*, *220*(8), 1207–1221. https://doi.org/10.1016/j.ijheh.2017.08.004
Gelormino, E., Melis, G., Marietta, C., & Costa, G. (2015). From built environment to health inequalities: An explanatory framework based on evidence. *Preventive Medicine Reports*, *2*, 737–745. https://doi.org/10.1016/j.pmedr.2015.08.019
Iacobucci, D., Posavac, S. S., Kardes, F. R., Schneider, M. J., & Popovich, D. L. (2015). The median split: Robust, refined, and revived. *Journal of Consumer Psychology*, *25*(4), 690–704. https://doi.org/10.1016/j.jcps.2015.06.014
Labib, S. M., Lindley, S., & Huck, J. J. (2020). Spatial dimensions of the influence of urban green-blue spaces on human health: A systematic review. *Environmental Research*, *180*, 108869. https://doi.org/10.1016/j.envres.2019.108869
Labib, S. M., Shuvo, F. K., Browning, M. H. E. M., & Rigolon, A. (2020). Noncommunicable diseases, park prescriptions, and urban green space use patterns in a Global South context: The Case of Dhaka, Bangladesh. *International Journal of Environmental Research and Public Health*, *17*(11), 3900. https://doi.org/10.3390/ijerph17113900
Markevych, I., Schoierer, J., Hartig, T., Chudnovsky, A., Hystad, P., Dzhambov, A. M., … Fuertes, E. (2017). Exploring pathways linking greenspace to health: Theoretical and methodological guidance. *Environmental Research*, *158*, 301–317. https://doi.org/10.1016/j.envres.2017.06.028
Radke, E. G., Braun, J. M., Nachman, R. M., & Cooper, G. S. (2020). Phthalate exposure and neurodevelopment: A systematic review and meta-analysis of human epidemiological evidence. *Environment International*, *137*, 105408. https://doi.org/10.1016/j.envint.2019.105408
Radke, E. G., Glenn, B., Galizia, A., Persad, A., Nachman, R., Bateson, T., … Cooper, G. S. (2019). Development of outcome-specific criteria for study evaluation in systematic reviews of epidemiology studies. *Environment International*, *130*, 104884. https://doi.org/10.1016/j.envint.2019.05.078
Razani, N., Niknam, K., Wells, N. M., Thompson, D., Hills, N. K., Kennedy, G., … Rutherford, G. W. (2019). Clinic and park partnerships for childhood resilience: A prospective study of park prescriptions. *Health and Place*, *57*, 179–185. https://doi.org/10.1016/j.healthplace.2019.04.008
Rigolon, A. (2016). A complex landscape of inequity in access to urban parks: A literature review. *Landscape and Urban Planning*, *153*, 160–169. https://doi.org/10.1016/j.landurbplan.2016.05.017
Rigolon, A., Browning, M. H. E. M., Lee, K., & Shin, S. (2018). Access to urban green space in cities of the Global South: A systematic literature review. *Urban Science*, *2*(3), 67–91. https://doi.org/10.3390/urbansci2030067
Robinson, O., Tamayo, I., de Castro, M., Valentin, A., Giorgis-Allemand, L., Hjertager Krog, N., … Basagaña, X. (2018). The urban exposome during pregnancy and its socioeconomic determinants. *Environmental Health Perspectives*, *126*(7), 077005. https://doi.org/10.1289/ehp2862
Schreinemachers, D. M. (2003). Birth malformations and other adverse perinatal outcomes in four U.S. wheat-producing states. *Environmental Health Perspectives*, *111*(9), 1259–1264.
Schüle, S. A., Hilz, L. K., Dreger, S., & Bolte, G. (2019). Social inequalities in environmental resources of green and blue spaces: A review of evidence in the WHO European region. *International Journal of Environmental Research and Public Health*, *16*, 1216. https://doi.org/10.3390/ijerph16071216
Twohig-Bennett, C., & Jones, A. (2018). The health benefits of the great outdoors: A systematic review and meta-analysis of greenspace exposure and health outcomes. *Environmental Research*, *166*, 628–637. https://doi.org/10.1016/j.envres.2018.06.030
van den Bosch, M., & Ode Sang, Å. (2017). Urban natural environments as nature-based solutions for improved public health – A systematic review of reviews. *Environmental Research*, *158*, 373–384. https://doi.org/10.1016/j.envres.2017.05.040
Wang, K., Cui, Q., & Xu, H. (2018). Desert as therapeutic space: Cultural interpretation of embodied experience in sand therapy in Xinjiang, China. *Health and Place*, *53*, 173–181.

https://doi.org/10.1016/j.healthplace.2018.08.005

Wen, Y., Yan, Q., Pan, Y., Gu, X., & Liu, Y. (2019). Medical empirical research on forest bathing (Shinrin-yoku): A systematic review. *Environmental Health and Preventive Medicine*, *24*(1), 1–21. https://doi.org/10.1186/s12199-019-0822-8

Zając, J., Bojar, I., Helbin, J., Kolarzyk, E., & Owoc, A. (2014). Salt caves as simulation of natural environment and significance of halotherapy. *Annals of Agricultural and Environmental Medicine*, *21*(1), 124–127.

Table S1. *Databases used in previous reviews on green space and health and on access to green space.*

**Source**	**PubMed**	**Medline**	**Cinahl**	**PsycINFO**	**Cochrane**	**Scopus**	**Ebsco or ERIC**	**WoS**	**AMED**	**Informit**	**Google Scholar**	**ProQuest**	**SPORTDIscus**	**Embase**	**CAB**	**KISS/RISS**	**DBpia**	**ScienceDirect**	**Ichushi-Web**	**Social Services Abstracts**	**CSA ILLUMINA**	**IngentaConnect**	**JSTOR**	**Int Index Black Periodicals**	**Wilson Web**	**Social Sciences Full Text**	**Sociological Abstracts**	**Education Full Text**	**Academic Search Complete**	**LILACS**	**GREAT**
Trøstrup et al (2019)	X		X	X	X																										
Browning & Rigolon (2019)						X	X	X																							
Lakhani et al (2019)		X	X					X																							
Lauwers et al (2019)	X							X																							
Vanaken & Danckaerts (2018)	X					X																									
Lambert et al (2018)		X	X			X		X	X	X	X	X																			
Rigolon et al (2018)						X		X																							
Stevenson et al (2018)		X		X		X		X					X	X																	
Zhang et al (2017		X				X		X							X																
Kabisch et al (2017)						X		X																							
Lambert et al (2017)		X	X			X		X	X	X	X	X		X																	
van den Bosch & Sang (2017)	X			X		X		X																							
Hutchinson & Shin (2014)	X	X																													
Cappucino et al (2010)		X			X									X																	
Lee et al (2017)	X		X	X	X									X		X	X														
Xu et al (2016)	X					X		X				X						X													
Ding et al (2013)	X													X																	

Source	PubMed	Medline	Cinahl	PsycINFO	Cochrane	Scopus	Ebsco or ERIC	WoS	AMED	Informit	Google Scholar	ProQuest	SPORTDIscus	Embase	CAB	KISS/RISS	DBpia	ScienceDirect	Ichushi-Web	Social Services Abstracts	CSA ILLUMINA	IngentaConnect	JSTOR	Int Index Black Periodicals	Wilson Web	Social Sciences Full Text	Sociological Abstracts	Education Full Text	Academic Search Complete	LILACS	GREAT
Kamioka et al (2012)	X	X	X					X											X												
Leal and Chaix	X																														
Reilly & Kelly (2010)		X				X								X																	
Jackson & Hodge (2009)		X		X			X					X								X	X	X	X	X	X	X	X	X			
Van den Beuken et al (2016)	X	X	X		X									X																	
Fütter et al (2015)		X			X									X																	
Rice et al (2016)	X	X									X			X																	
Didsbury et al (2016)		X		X										X																	
Bagnardi et al (2014)		X						X						X																	
Gunn et al (2016)	X	X	X					X						X													X				
Yi et al (2015)	X													X																	
Zuccotti et al (2015)	X				X									X																	
Kim et al (2016)		X			X	X		X						X															X	X	X
Banay et al (2017)		X												X																	
Rigolon (2016)							X	X										X					X								
Gascon et al (2016)		X				X																									

Note: PubMed includes Medline, but some reviews only used Medline.

Table S2. *Search terms used in reviews on green space and health and on access to green space.*

Construct	Search Terms	Source
Green space	"Nature-based" OR "Open space*" OR Garden* OR Horticultur* OR Wilder- ness OR Countryside OR Outdoor* OR landscape* OR botanic* OR nature*	Trøstrup et al (2019)
Green space	"green space" OR "greenspace" OR "tree cover*" OR "natural environment*" OR "nearby nature"	Browning & Rigolon (2019)
Green space	"horticulture*" OR "natural environ- ment" OR "nature assisted" OR "nature-assisted" OR "nature interven- tion" OR "nature-based" OR "green" OR "wilderness" OR "garden*" OR "forest" OR "tree*" OR "plant*" OR "outdoor*" OR "park" OR "eco- therapy" OR "care farming"	Lakhani et al (2019)
Green space	"biological diversity" OR biodivers* OR "living natural resource*" OR "living resource*" OR "natur* diversity" OR "diversity in nature" OR "*species diversity" OR "int*-speci* diversity" OR "genetic diversity" OR "diversity of gene*" OR ecosystem* OR "ecological system*" OR "ecosystem service*" OR "landscape service*" OR "environmental ser- vice*" OR "ecological service*" OR "natur* capital*" OR "nature based solution*" OR "environmental capital*" OR "green infrastructure" OR greenspace* OR "green space*" OR "blue infrastructure" OR bluespace* OR "blue space*" OR flora* OR fauna* OR wildlife OR "natural habitat*" OR "ecological habitat" OR "wildlife habitat*" OR "invasive * species" OR biogeograph* OR "bio-geograph*" OR "natur* space*" OR "natur* environment*"	Lauwers et al (2019)
Atopic disease	allergic sen*" OR atopy OR atopic OR Hypersensitivity OR IgE OR "Immunoglobulin E" OR Sensiti?ation	Lambert et al (2018)
Green space	NDVI OR "Normali?ed Difference Vegetation Index" OR "Enhanced Vegetation Index" OR "residential green*" OR "green space"	Lambert et al (2018)
Green space	greenspace * OR "green space*" OR "green area*" OR "open space*" OR "urban park*" OR park* OR "neighborhood amenit*"	Rigolon et al (2018)
Green space	"views of nature," "contact with nature," "near-home nature," "nearby nature," "naturalness," and "green space")	Stevenson et al (2018)
Physical limitations	- Walking disabilities - Walking handicaps - Physical disabilities - Physical impairments - Mobility problems/impairments - Elderly - OR some specific targets: - Cerebral palsy – Rheumatism – Muscular dystrophy - Parkinson's - Spina bifida - Spinal cord injury – Sclerosis – Polio – Arthritis – Osteoporosis - Neuromuscular disorders	Zhang et al (2017)
Green space	TITLE-ABS-KEY ("green area*") OR TITLE-ABS-KEY ("blue area*") OR TITLE-ABS-KEY ("green space*") OR TITLE-ABS-KEY ("blue space*") OR TITLE-ABS-KEY ("natural environment*") OR TITLE- ABS-KEY ("urban water") OR TITLE-ABS-KEY ("urban park*") OR TITLE-ABS-KEY ("urban forest*") OR TITLE-ABS-KEY (garden*) OR TITLE-ABS-KEY ("urban tree*") OR TITLE-ABS-KEY ("green infrastructure") OR TITLE-ABS-KEY ("green roof*") OR TITLE-ABS- KEY ("green facade*") OR TITLE-ABS-KEY ("green wall*") OR TITLE-ABS-KEY ("biophilic design")	Kabisch et al (2017)
General health	(TITLE-ABS-KEY (health) OR TITLE-ABS-KEY (diseas*) OR TITLE-ABS-KEY (health) OR TITLE-ABS-KEY (disease*) OR TITLE-ABS-KEY (disorder*) OR TITLE-ABS-KEY ("well-being") OR TITLE-ABS-KEY ("quality of life") OR TITLE-ABS-KEY (illness*) OR TITLE-ABS-KEY (mortality) OR TITLE-ABS-KEY (morbidity) OR TITLE-ABS-KEY (lung function) OR TITLE-ABS-KEY (bronchit*) OR TITLE-ABS-KEY (respirat*) OR TITLE-ABS-KEY (asthma) OR TITLE-ABS-KEY ("mental health") OR TITLE-ABS-KEY ("physical health") OR TITLE-ABS-KEY (obes*) OR TITLE-ABS-KEY (overweight) OR TITLE-ABS-KEY (diabetes) OR TITLE-ABS-KEY ("sleep disturbance") OR TITLE-ABS-KEY (depression))	Kabisch et al (2017)
Atopic disease	"allergic respiratory disease*" OR Asthma OR Wheeze OR "allegic rhinitis" OR "allergic rhin*" OR Hayfever OR "Lung function" OR spirometry OR FEV1 OR "forced expiratory volume" OR "forced vital capacity"	Lambert et al (2017)
Green space	"Green space*" "Urban park*" "urban forest*"	van den Bosch & Sang (2017)

Construct	Search Terms	Source
	"urban tree*" Biodiversity (AND urban) "blue space*"<br>Water (AND urban)	
General health and mechanisms	"Physical activity*" walkability OR Stress OR Restoration OR Affect* OR "social cohesion" OR "social capital" OR "air pollution" OR "air quality" OR Cooling OR Heat OR Noise OR "water management" Flooding OR food OR Inequalit* OR Overweight OR Obesity OR immun* OR "Public health" OR "Human health" Well-being OR Health OR "quality of life" OR attention OR Development (AND child*) "birth weight" OR Morbidity OR Mortality OR Illness* OR Disease* OR Disorder* OR cardiovascular OR Cancer OR Asthma OR Depression OR anxiety	van den Bosch & Sang (2017)
Native Americans, racially/ethnically minoritized group experiencing health disparities	'Native American,'' ''American Indian,'' ''Alaska Native'' and ''health status,'' ''health disparities,''	Hutchinson & Shin (2014)
Cardiovascular disease and associated factors	''diabetes,'' ''cardiovascular disease,'' ''stroke,'' ''acute MI,'' ''heart disease,'' ''metabolic syndrome,'' ''hypertension,'' ''obesity,'' ''hyperlipidemia.''	Hutchinson & Shin (2014)
Mortality	"mortality" OR "death"	Cappucino et al (2010)
Green space	1. Trees * OR Tree OR 3. Forests OR 4. Forest OR 5. Forest Area OR 6. Area, Forested OR 7. Areas, Forested OR Forested Area OR 9. Woodland OR 10. Woodlands OR 11. Forestlands OR 12. Forestland OR 13. Wood OR 14. Woods OR 15. Shinrinyoku OR 16. Green exercise	Lee et al (2017)
Mortality	death" OR "mortality"	Xu et al (2016)
Cardiovascular disease	cardio- vascular disease," "coronary heart disease," "stroke," "mortality," "heart failure," "myocardial infarction," "ischemic heart disease," "sudden cardiac arrest," and "acute coronary syndrome."	Ding et al (2013)
Cardiovascular disease and associated factors	'Obesity', 'Overweight', 'Blood pressure', 'Diabetes Mellitus', 'Dyslipidemias' or 'Metabolic Diseases'	Leal and Chaix (2011)
Obesity and associated factors	obes$ or over- weight$ or bmi or adiposity$ or fat$ or weight$)	Reilly and Kelly (2010)
Native Americans, racially/ethnically minoritized experiencing health disparities	"Native American,'' ''American Indian,'' ''ethnic minority,'' ''minority,''	Jackson & Hodge (2009)
Cancer	''cancer,'' ''neoplasm,'' ''terminal,'' ''end-stage,'' ''advanced,'' ''hospice,'' or ''palliative''	Van den Beuken et al (2016)
Native Americans	First Nations OR Aboriginal OR Indigenous OR Inuit OR Inuvialuit	Rice et al (2016)
Diabetes and related factors	Metis OR diabetes OR hypertension OR high blood pressure OR chronic disease OR chronic condition OR obesity	Rice et al (2016)
Disadvantaged populations and health inequities	exp socioeconomic factors OR socio economic factors OR. IR socioeconomic factors OR. OR (socioeconomic adj3 conditions) OR.<br>OR (socio economic adj3 conditions) OR. OR (socio economic adj3 disadvant$) OR. 12 (socioeconomic adj3 disadvant$). OR (socio economic adj3 position) OR. 14 (socioeconomic adj3 position) OR. 15 socio economic status OR 6 socioeconomic status OR Social Class/ 19 social class OR. 20 exp social conditions/ 21 social conditions OR. 22 Poverty/ 23 poverty OR. 24 health status disparities/	Didsbury et al (2016)
Diabetes	("diabetes mellitus, type 2"[mh] or (diabet*[tiab] and ("non-insulin dependent"[tiab] or type-2[tiab] or "type II"[tiab] or "type 2"[tiab])))	Maruthur et al (2016)
Cancer	lip neoplasms OR tongue neoplasms OR salivary gland neoplasms OR gingival neoplasms OR mouth neoplasms OR pharyngeal neoplasms OR esophageal neoplasms OR intestinal neoplasms OR stomach neoplasms OR colorectal neoplasms OR liver neoplasms OR gallbladder neoplasms OR pancreatic neoplasms OR laryngeal neoplasms OR lung neoplasms OR carcinoma, basal cell	Bagnardi et al (2014)

Construct	Search Terms	Source
	OR melanoma OR carcinoma, squamous cell OR breast neoplasms OR uterine cervical neoplasms OR endometrial neoplasms OR ovarian neoplasms OR prostatic neoplasms OR kidney neoplasms OR renal cell carcinoma OR urinary bladder neoplasms OR thyroid neoplasms OR brain neoplasms OR Non-Hodgkin lymphoma OR Hodgkin disease OR neoplasms OR lip cancer OR tongue cancer OR salivary gland cancer OR gingival cancer OR mouth cancer OR pharyngeal cancer OR esophageal cancer OR intestinal cancer OR stomach cancer OR colorectal cancer OR liver cancer OR gallbladder cancer OR pancreatic cancer OR laryngeal cancer OR lung cancer OR skin cancer OR basal cell carcinoma OR squamous cell carcinoma OR melanoma OR breast cancer OR uterine cervical cancer OR endometrial cancer OR ovarian cancer OR prostatic cancer OR kidney cancer OR renal cell carcinoma OR urinary bladder cancer OR thyroid cancer OR brain cancer OR Non-Hodgkin lymphoma OR Hodgkin disease OR cancer	
Birth outcomes	"Infant, Newborn"[Mesh] 30. "low birth weight"[tw] 31. "low birthweight"[tw] 32. "small for gestational age"[tw] 33. "infant"[tw] 34. "fetal"[tw] 35. "fetus"[tw] 36. "neonate"[All Fields] 37. "Embryonic and Fetal Development"[Mesh] 38. "embryology"[Mesh] 39. "infant mortality"[Mesh] 40. "maternal mortality"[Mesh] 41. "prenatal injuries"[Mesh] 42. "Child Development"[Mesh] 43. "Pregnancy"[Mesh] 44. "Pregnancy"[tw] 45. "Female Urogenital Diseases and Pregnancy Complications"[Mesh] 46. "Breast Feeding"[Mesh] 47. "Maternal-Child Nursing"[Mesh] 48. "Postpartum Period"[Mesh])	Gunn et al (2016)
Birth outcomes	"bariatric surgery," "pregnancy," "obstetric," "maternal," "neonatal," "perinatal," and "fetal."	Yi et al (2015)
Atopic disease	atopic eczema OR atopic dermatit* OR asthma OR asthma* OR allergic rhinitis OR allergic rhinit* wheeze OR wheez* OR hay fever OR (IgE AND (sensitisation OR sensitization)) OR allergy OR allergic OR atopy OR atopic)	Zuccotti et al (2015)
Atopic disease	1. explode DERMATITIS, ATOPIC/ 2. atopic dermatitis.mp. 3. dermatitis atopic.mp. 4. explode ECZEMA/or eczema.mp 5. childhood eczema.mp. 6. infantile eczema.mp. 7. neurodermatitis.mp. or exp Neurodermatitis/ 8. Besnier's prurigo.mp	Kim et al (2016)
General health	1. Allerg * 2. Asthma 3. BMI 4. *Morbidity 5. Mortality 6. Obesity 7. Physical activity 8. Physical health 9. Pregnancy	Browning & Lee (2017)
Maternal fetal outcomes	birth outcomes", "preterm birth", "premature birth", "low birth weight (LBW)", "intrauterine growth retardation", "small for gestational age (SGA)", "pregnancy outcomes", "maternal outcomes", "reproductive outcomes", "preeclampsia", "gestational diabetes", "spontaneous abortion", "pregnancy loss", "maternal depression", "postpartum depression", or "peripartum depression"	Banay et al (2017)
Green space	"park," "green space," "open space," "playground," or "recreation,"	Rigolon (2016)
Mortality	Mortality, survival, life expectancy	Gascon et al (2016)

References

Bagnardi, V., Rota, M., Botteri, E., Tramacere, I., Islami, F., Fedirko, V., et al. (2014). Alcohol consumption and site-specific cancer risk: a comprehensive dose–response meta-analysis. *British Journal of Cancer*, *112*(3), 580–593. http://doi.org/10.1038/bjc.2014.579

Banay, R., Bezold, C., James, P., Hart, J., & Laden, F. (2017). Residential greenness: current perspectives on its impact on maternal health and pregnancy outcomes. *International Journal of Women's Health*, *Volume 9*, 133–144. http://doi.org/10.2147/IJWH.S125358

Browning, M., & Lee, K. (2017). Within what distance does "greenness" best predict physical health? A systematic review of articles with GIS buffer analyses across the lifespan. *International Journal of Environmental Research and Public Health*, *14*(7). http://doi.org/10.3390/ijerph14070675

Browning, M., & Rigolon, A. (2019). School green space and Its impact on academic performance: A systematic literature review. *International Journal of Environmental Research and Public Health*, *16*(3), 429–22. http://doi.org/10.3390/ijerph16030429

Didsbury, M. S., Kim, S., Medway, M. M., Tong, A., McTaggart, S. J., Walker, A. M., et al. (2016). Socio-economic status and quality of life in children with chronic disease: A systematic review. *Journal of Paediatrics and Child Health*, *52*(12), 1062–1069. http://doi.org/10.1111/jpc.13407

Ding, M., Bhupathiraju, S. N., Satija, A., & van Dam, R. M. (2013). Long-term coffee consumption and risk of cardiovascular disease: a systematic review and a dose-response meta-analysis of prospective cohort studies. *Circulation*. http://doi.org/10.1161/CIRCULATIONAHA.113.005925/-/DC1

Fütterer, J. J., Briganti, A., De Visschere, P., Emberton, M., Giannarini, G., Kirkham, A., et al. (2015). Can Clinically Significant Prostate Cancer Be Detected with Multiparametric Magnetic Resonance Imaging? A Systematic Review of the Literature. *European Urology*, *68*(6), 1045–1053. http://doi.org/10.1016/j.eururo.2015.01.013

Gascon, M., Triguero-Mas, M., Martínez, D., Dadvand, P., Rojas-Rueda, D., Plasència, A., & Nieuwenhuijsen, M. (2016). Residential green spaces and mortality: A systematic review. *Environment International*, *86*(C), 60–67. http://doi.org/10.1016/j.envint.2015.10.013

Gunn, J. K. L., Rosales, C. B., Center, K. E., Nuñez, A., Gibson, S. J., Christ, C., & Ehiri, J. E. (2016). Prenatal exposure to cannabis and maternal and child health outcomes: a systematic review and meta-analysis. *BMJ Open*, *6*(4), e009986–8. http://doi.org/10.1136/bmjopen-2015-009986

Hutchinson, R. N., & Shin, S. (2014). Systematic Review of Health Disparities for Cardiovascular Diseases and Associated Factors among American Indian and Alaska Native Populations. *PLoS ONE*, *9*(1), e80973–9. http://doi.org/10.1371/journal.pone.0080973

Kabisch, N., van den Bosch, M., & Lafortezza, R. (2017). The health benefits of nature-based solutions to urbanization challenges for children and the elderly – A systematic review. *Environmental Research*, *159*, 362–373. http://doi.org/10.1016/j.envres.2017.08.004

Kamioka, H., Tsutani, Mutoh, Honda, T., Shiozawa, Park, et al. (2012). A systematic review of randomized controlled trials on curative and health enhancement effects of forest therapy. *Psychology Research and Behavior Management*, 85–11. http://doi.org/10.2147/PRBM.S32402

Kim, J. P., Chao, L. X., Simpson, E. L., & Silverberg, J. I. (2016). Persistence of atopic dermatitis (AD): A systematic review and meta-analysis. *Journal of the American Academy of Dermatology*, *75*(4), 681–687.e11. http://doi.org/10.1016/j.jaad.2016.05.028

Lakhani, A., Norwood, M., Watling, D. P., Zeeman, H., & Kendall, E. (2019). Using the natural environment to address the psychosocial impact of neurological disability_ A systematic review. *Health & Place*, *55*, 188–201. http://doi.org/10.1016/j.healthplace.2018.12.002

Lambert, K. A., Bowatte, G., Tham, R., Lodge, C., Prendergast, L., Heinrich, J., et al. (2017). Residential greenness and allergic respiratory diseases in children and adolescents – A systematic review and meta-analysis. *Environmental Research*, *159*, 212–221. http://doi.org/10.1016/j.envres.2017.08.002

Lauwers, L., Bastiaens, H., Remmen, R., & Keune, H. (2019). The Integration of Interlinkages Between Nature and Human Health in Primary Health Care: Protocol for a Scoping Review. *JMIR Research Protocols*, *8*(1), e12510–9. http://doi.org/10.2196/12510

Leal, C., & Chaix, B. (2011). The influence of geographic life environments on cardiometabolic risk factors: a systematic review, a methodological assessment and a research agenda. *Obesity Reviews*, *12*(3), 217–230. http://doi.org/10.1111/j.1467-789X.2010.00726.x

Lee, I., Choi, H., Bang, K.-S., Kim, S., Song, M., & Lee, B. (2017). Effects of forest therapy on depressive symptoms among adults: A systematic review. *International Journal of Environmental Research and Public Health*, *14*(3), 321–18. http://doi.org/10.3390/ijerph14030321

Maruthur, N. M., Tseng, E., Hutfless, S., Wilson, L. M., Suarez-Cuervo, C., Berger, Z., et al. (2016). Diabetes Medications as Monotherapy or Metformin-Based Combination Therapy for Type 2 Diabetes. *Annals of Internal Medicine*, *164*(11), 740–19. http://doi.org/10.7326/M15-2650

Reilly, J. J., & Kelly, J. (2010). Long-term impact of overweight and obesity in childhood and adolescence on morbidity and premature mortality in adulthood: systematic review. *International Journal of Obesity*, *35*(7), 891–898. http://doi.org/10.1038/ijo.2010.222

Rice, K., Hiwi, Te, B., Zwarenstein, M., Lavallee, B., Barre, D. E., Harris, S. B., FORGE AHEAD program team. (2016). Best Practices for the Prevention and Management of Diabetes and Obesity-Related Chronic Disease among Indigenous Peoples in Canada: A Review. *Canadian Journal of Diabetes*, *40*(3), 216–225. http://doi.org/10.1016/j.jcjd.2015.10.007

Rigolon, A. (2016). A complex landscape of inequity in access to urban parks: A literature review. *Landscape and Urban Planning*, *153*, 160–169. http://doi.org/10.1016/j.landurbplan.2016.05.017

Rigolon, A., Browning, M., Lee, K., & Shin, S. (2018). Access to Urban Green Space in Cities of the Global South: A Systematic Literature Review. *Urban Science*, *2*(3), 67. http://doi.org/10.3390/urbansci2030067

Stevenson, M. P., Schilhab, T., & Bentsen, P. (2018). Attention Restoration Theory II: a systematic review to clarify attention processes affected by exposure to natural environments. *Journal of Toxicology and Environmental Health, Part B*, *21*(4), 227–268. http://doi.org/10.1080/10937404.2018.1505571

Trøstrup, C. H. (2019). The effect of nature exposure on the mental health of patients: a systematic review. *Quality of Life Research*, *0*(0), 0–0. http://doi.org/10.1007/s11136-019-02125-9

van den Beuken-van Everdingen, M. H. J., Hochstenbach, L. M. J., Joosten, E. A. J., Tjan-Heijnen, V. C. G., Janssen, D. J. A. (2016). Update on Prevalence of Pain in Patients with Cancer: Systematic Review and Meta-Analysis. *Journal of Pain and Symptom Management*, *51*(6), 1070–1090.e9. http://doi.org/10.1016/j.jpainsymman.2015.12.340

van den Bosch, M., & Sang, Å. O. (2017). Urban natural environments as nature-based solutions for improved public health – A systematic review of reviews. *Environmental Research*, *158*, 373–384. http://doi.org/10.1016/j.envres.2017.05.040

Vanaken, G.-J., & Danckaerts, M. (2018). Impact of Green Space Exposure on Children's and Adolescents' Mental Health: A Systematic Review. *International Journal of Environmental Research and Public Health*, *15*(12), 2668. http://doi.org/10.3390/ijerph15122668

Lambert, K., Bowatte, G., Tham, R., Lodge, C., Prendergast, L., Heinrich, J., et al. (2018). Greenspace and Atopic Sensitization in Children and Adolescents—A Systematic Review. *International Journal of Environmental Research and Public Health*, *15*(11), 2539. http://doi.org/10.3390/ijerph15112539

Xu, Z., FitzGerald, G., Guo, Y., Jalaludin, B., & international, S. T. E. (2016). Impact of heatwave on mortality under different heatwave definitions: a systematic review and meta-analysis. *Landscape and Urban Planning*, *89-90*, 193–203. http://doi.org/10.1016/j.envint.2016.02.007

Yi, X.-Y., Li, Q.-F., Zhang, J., & Wang, Z.-H. (2015). A meta-analysis of maternal and fetal outcomes of pregnancy after bariatric surgery. *International Journal of Gynecology & Obstetrics*, *130*(1), 3–9. http://doi.org/10.1016/j.ijgo.2015.01.011

Zhang, G., Poulsen, D., Lygum, V., Corazon, S., Gramkow, M., & Stigsdotter, U. (2017). Health-Promoting Nature Access for People with Mobility Impairments: A Systematic Review. *International Journal of Environmental Research and Public Health*, *14*(12), 703–19. http://doi.org/10.3390/ijerph14070703

Zuccotti, G., Meneghin, F., Aceti, A., Barone, G., Callegari, M. L., Di Mauro, A., et al. (2015). Probiotics for prevention of atopic diseases in infants: systematic review and meta-analysis. *Allergy*, *70*(11), 1356–1371. http://doi.org/10.1111/all.12700

Table S3. *Search expressions used in this review.*

Database	Health outcome	Search expression
CINAHL	Mortality	((TI ("parks" OR "natural environment*" OR "green space*" OR greenspace* OR "blue space*" OR bluespace* OR greenness OR "street tree*" OR "green exercise*" OR "urban tree*") AND ("mortalit*" OR "life expentanc*")) AND PT academic journal) OR ((AB ("parks" OR "natural environment*" OR "green space*" OR greenspace* OR "blue space*" OR bluespace* OR greenness OR "street tree*" OR "green exercise*" OR "urban tree*") AND ("mortalit*" OR "life expentanc*")) AND PT academic journal) OR ((MW ("parks" OR "natural environment*" OR "green space*" OR greenspace* OR "blue space*" OR bluespace* OR greenness OR "street tree*" OR "green exercise*" OR "urban tree*") AND ("mortalit*" OR "life expentanc*")) AND PT academic journal) OR ((SU ("parks" OR "natural environment*" OR "green space*" OR greenspace* OR "blue space*" OR bluespace* OR greenness OR "street tree*" OR "green exercise*" OR "urban tree*") AND ("mortalit*" OR "life expentanc*")) AND PT academic journal)
CINAHL	Cardiovascular health/disease and obesity	((TI ("parks" OR "natural environment*" OR "green space*" OR greenspace* OR "blue space*" OR bluespace* OR greenness OR "street tree*" OR "green exercise*" OR "urban tree*") AND ("obes*" OR "overweight*" OR "BMI*" OR "adipos*" OR "cardiovascular*" OR "acute MI" OR "myocardial infarction*" OR "ischemic heart disease*" OR "sudden cardiac arrest*" OR "heart failure*" OR "acute coronary syndrome*" OR "cardiometabolic" OR "hypertension" OR "sudden cardiac death*")) AND PT academic journal) OR ((AB ("parks" OR "natural environment*" OR "green space*" OR greenspace* OR "blue space*" OR bluespace* OR greenness OR "street tree*" OR "green exercise*" OR "urban tree*") AND ("obes*" OR "overweight*" OR "BMI*" OR "adipos*" OR "cardiovascular*" OR "acute MI" OR "myocardial infarction*" OR "ischemic heart disease*" OR "sudden cardiac arrest*" OR "heart failure*" OR "acute coronary syndrome*" OR "cardiometabolic" OR "hypertension" OR "sudden cardiac death*")) AND PT academic journal) OR ((MW ("parks" OR "natural environment*" OR "green space*" OR greenspace* OR "blue space*" OR bluespace* OR greenness OR "street tree*" OR "green exercise*" OR "urban tree*") AND ("obes*" OR "overweight*" OR "BMI*" OR "adipos*" OR "cardiovascular*" OR "acute MI" OR "myocardial infarction*" OR "ischemic heart disease*" OR "sudden cardiac arrest*" OR "heart failure*" OR "acute coronary syndrome*" OR "cardiometabolic" OR "hypertension" OR "sudden cardiac death*")) AND PT academic journal) OR ((SU ("parks" OR "natural environment*" OR "green space*" OR greenspace* OR "blue space*" OR bluespace* OR greenness OR "street tree*" OR "green exercise*" OR "urban tree*") AND ("obes*" OR "overweight*" OR "BMI*" OR "adipos*" OR "cardiovascular*" OR "acute MI" OR "myocardial infarction*" OR "ischemic heart disease*" OR "sudden cardiac arrest*" OR "heart failure*" OR "acute coronary syndrome*" OR "cardiometabolic" OR "hypertension" OR "sudden cardiac death*")) AND PT academic journal)
CINAHL	Cancer	TI (("parks" OR "natural environment*" OR "green space*" OR greenspace* OR "blue space*" OR bluespace* OR greenness OR "street tree*" OR "green exercise*" OR "urban tree*") AND ("neoplasm*" OR "carcinoma" OR "cancer*" OR "lymphoma" OR "Hodgkin")) AND PT academic journal) OR AB (("parks" OR "natural environment*" OR "green space*" OR greenspace* OR "blue space*" OR bluespace* OR greenness OR "street tree*" OR "green exercise*" OR "urban tree*") AND ("neoplasm*" OR "carcinoma" OR "cancer*" OR "lymphoma" OR "Hodgkin")) AND PT academic journal) OR MW (("parks" OR "natural environment*" OR "green space*" OR greenspace* OR "blue space*" OR bluespace* OR greenness OR "street tree*" OR "green exercise*" OR "urban tree*") AND ("neoplasm*" OR "carcinoma" OR "cancer*" OR "lymphoma" OR "Hodgkin")) AND PT academic journal) OR SU (("parks" OR "natural environment*" OR "green space*" OR greenspace* OR "blue space*" OR bluespace* OR greenness OR "street tree*" OR "green exercise*" OR "urban tree*") AND ("neoplasm*" OR "carcinoma" OR "cancer*" OR "lymphoma" OR "Hodgkin")) AND PT academic journal)
CINAHL	Diabetes	TI (("parks" OR "natural environment*" OR "green space*" OR greenspace* OR "blue space*" OR bluespace* OR greenness OR "street tree*" OR "green exercise*" OR "urban tree*") AND ("diabet*" OR "insulin") AND PT academic journal) OR AB (("parks" OR "natural environment*" OR "green space*" OR greenspace* OR "blue space*" OR bluespace* OR greenness OR "street tree*" OR "green exercise*" OR "urban tree*") AND ("diabet*" OR "insulin") AND PT academic journal) OR MW (("parks" OR "natural environment*" OR "green space*" OR greenspace* OR "blue space*" OR bluespace* OR greenness OR "street tree*" OR "green exercise*" OR "urban tree*") AND ("diabet*" OR "insulin") AND PT academic journal) OR SU (("parks" OR "natural environment*" OR "green space*" OR greenspace* OR "blue space*" OR

Database	Health outcome	Search expression
		bluespace* OR greenness OR "street tree*" OR "green exercise*" OR "urban tree*") AND ("diabet*" OR "insulin") AND PT academic journal)
CINAHL	Atopic disease	TI (("parks" OR "natural environment*" OR "green space*" OR greenspace* OR "blue space*" OR bluespace* OR greenness OR "street tree*" OR "green exercise*" OR "urban tree*") AND ("allergic respiratory disease*" OR "asthma*" OR "wheez*" OR "allergic rhin*" OR "hayfever" OR "lung function*" OR "spirometry" OR "FEV1" OR "forced expiratory volume*" OR "forced vital capacity*" OR "atopy" OR "eczema" OR "atopic dermatit*" OR (ige AND (sensitization OR sensitization)) OR "allerg*" OR "atopic dermatitis" OR "dermatitis atopic" OR "neurodermatitis" OR "Besnier's prurigo" OR "respirat*") AND PT academic journal) OR AB (("parks" OR "natural environment*" OR "green space*" OR greenspace* OR "blue space*" OR bluespace* OR greenness OR "street tree*" OR "green exercise*" OR "urban tree*") AND ("allergic respiratory disease*" OR "asthma*" OR "wheez*" OR "allergic rhin*" OR "hayfever" OR "lung function*" OR "spirometry" OR "FEV1" OR "forced expiratory volume*" OR "forced vital capacity*" OR "atopy" OR "eczema" OR "atopic dermatit*" OR (ige AND (sensitization OR sensitization)) OR "allerg*" OR "atopic dermatitis" OR "dermatitis atopic" OR "neurodermatitis" OR "Besnier's prurigo" OR "respirat*") AND PT academic journal) OR MW (("parks" OR "natural environment*" OR "green space*" OR greenspace* OR "blue space*" OR bluespace* OR greenness OR "street tree*" OR "green exercise*" OR "urban tree*") AND ("allergic respiratory disease*" OR "asthma*" OR "wheez*" OR "allergic rhin*" OR "hayfever" OR "lung function*" OR "spirometry" OR "FEV1" OR "forced expiratory volume*" OR "forced vital capacity*" OR "atopy" OR "eczema" OR "atopic dermatit*" OR (ige AND (sensitization OR sensitization)) OR "allerg*" OR "atopic dermatitis" OR "dermatitis atopic" OR "neurodermatitis" OR "Besnier's prurigo" OR "respirat*") AND PT academic journal) OR SU (("parks" OR "natural environment*" OR "green space*" OR greenspace* OR "blue space*" OR bluespace* OR greenness OR "street tree*" OR "green exercise*" OR "urban tree*") AND ("allergic respiratory disease*" OR "asthma*" OR "wheez*" OR "allergic rhin*" OR "hayfever" OR "lung function*" OR "spirometry" OR "FEV1" OR "forced expiratory volume*" OR "forced vital capacity*" OR "atopy" OR "eczema" OR "atopic dermatit*" OR (ige AND (sensitization OR sensitization)) OR "allerg*" OR "atopic dermatitis" OR "dermatitis atopic" OR "neurodermatitis" OR "Besnier's prurigo" OR "respirat*") AND PT academic journal)
CINAHL	Birth outcomes	TI (("parks" OR "natural environment*" OR "green space*" OR greenspace* OR "blue space*" OR bluespace* OR greenness OR "street tree*" OR "green exercise*" OR "urban tree*") AND ("birth outcome*" OR "preterm birth*" OR "premature birth*" OR "low birth weight*" OR "low birthweight*" OR "intrauterine growth retardation" OR "small for gestational age" OR "pregnancy outcome*" OR "maternal outcome*" OR "reproductive outcome*" OR "preeclampsia" OR "gestational diabetes" OR "spontaneous abortion" OR "pregnancy loss*" OR "neonatal" OR "perinatal" OR "fetal" OR "fetus" OR "pregnancy" OR "embryonic" OR "infant" OR "maternal mortality" OR "postpartum") AND PT academic journal) OR AB (("parks" OR "natural environment*" OR "green space*" OR greenspace* OR "blue space*" OR bluespace* OR greenness OR "street tree*" OR "green exercise*" OR "urban tree*") AND ("birth outcome*" OR "preterm birth*" OR "premature birth*" OR "low birth weight*" OR "low birthweight*" OR "intrauterine growth retardation" OR "small for gestational age" OR "pregnancy outcome*" OR "maternal outcome*" OR "reproductive outcome*" OR "preeclampsia" OR "gestational diabetes" OR "spontaneous abortion" OR "pregnancy loss*" OR "neonatal" OR "perinatal" OR "fetal" OR "fetus" OR "pregnancy" OR "embryonic" OR "infant" OR "maternal mortality" OR "postpartum") AND PT academic journal) OR MW (("parks" OR "natural environment*" OR "green space*" OR greenspace* OR "blue space*" OR bluespace* OR greenness OR "street tree*" OR "green exercise*" OR "urban tree*") AND ("birth outcome*" OR "preterm birth*" OR "premature birth*" OR "low birth weight*" OR "low birthweight*" OR "intrauterine growth retardation" OR "small for gestational age" OR "pregnancy outcome*" OR "maternal outcome*" OR "reproductive outcome*" OR "preeclampsia" OR "gestational diabetes" OR "spontaneous abortion" OR "pregnancy loss*" OR "neonatal" OR "perinatal" OR "fetal" OR "fetus" OR "pregnancy" OR "embryonic" OR "infant" OR "maternal mortality" OR "postpartum") AND PT academic journal) OR SU (("parks" OR "natural environment*" OR "green space*" OR greenspace* OR "blue space*" OR bluespace* OR greenness OR "street tree*" OR "green exercise*" OR "urban tree*") AND ("birth outcome*" OR "preterm birth*" OR "premature birth*" OR "low birth weight*" OR "low birthweight*" OR "intrauterine growth retardation" OR "small for gestational age" OR "pregnancy outcome*" OR

Database	Health outcome	Search expression
		"maternal outcome*" OR "reproductive outcome*" OR "preeclampsia" OR "gestational diabetes" OR "spontaneous abortion" OR "pregnancy loss*" OR "neonatal" OR "perinatal" OR "fetal" OR "fetus" OR "pregnancy" OR "embryonic" OR "infant" OR "maternal mortality" OR "postpartum") AND PT academic journal)
CINAHL	General health	TI (("parks" OR "natural environment*" OR "green space*" OR greenspace* OR "blue space*" OR bluespace* OR greenness OR "street tree*" OR "green exercise*" OR "urban tree*") AND ("physical health" OR "chronic condition*" OR "*morbidit*" OR "self-reported health" OR "perceived health") AND PT academic journal) OR AB (("parks" OR "natural environment*" OR "green space*" OR greenspace* OR "blue space*" OR bluespace* OR greenness OR "street tree*" OR "green exercise*" OR "urban tree*") AND ("physical health" OR "chronic condition*" OR "*morbidit*" OR "self-reported health" OR "perceived health") AND PT academic journal) OR MW (("parks" OR "natural environment*" OR "green space*" OR greenspace* OR "blue space*" OR bluespace* OR greenness OR "street tree*" OR "green exercise*" OR "urban tree*") AND ("physical health" OR "chronic condition*" OR "*morbidit*" OR "self-reported health" OR "perceived health") AND PT academic journal) OR SU (("parks" OR "natural environment*" OR "green space*" OR greenspace* OR "blue space*" OR bluespace* OR greenness OR "street tree*" OR "green exercise*" OR "urban tree*") AND ("physical health" OR "chronic condition*" OR "*morbidit*" OR "self-reported health" OR "perceived health") AND PT academic journal
PubMed	Mortality	((parks[tw] OR "natural environment*"[tw] OR "green space*"[tw] OR "greenspace*"[tw] OR "blue space*"[tw] OR "bluespace*"[tw] OR "greenness"[tw] OR "street tree*"[tw] OR "green exercise*"[tw] OR "urban tree*"[tw]) AND ("mortality"[tw] OR “mortalities”[TW] OR "life expectancy"[tw] OR “life expectancies”[tw)) AND ("clinical trial"[Publication Type] OR "journal article"[Publication Type] OR "meta analysis"[Publication Type] OR "review"[Publication Type] OR "systematic review"[Publication Type])
PubMed	Cardiovascular health/disease and obesity	((parks[tw] OR "natural environment*"[tw] OR "green space*"[tw] OR "greenspace*"[tw] OR "blue space*"[tw] OR "bluespace*"[tw] OR "greenness"[tw] OR "street tree*"[tw] OR "green exercise*"[tw] OR "urban tree*"[tw]) AND ("obes*"[tw] OR "overweight*"[tw] OR "BMI*"[tw] OR "adipos*"[tw] OR "cardiovascular*"[tw] OR "acute MI"[tw] OR "myocardial infarction*"[tw] OR "ischemic heart disease*"[tw] OR "sudden cardiac arrest*"[tw] OR "heart failure*"[tw] OR "acute coronary syndrome*"[tw] OR "cardiometabolic"[tw] OR "hypertension"[tw] OR "sudden cardiac death*"[tw])) AND ("clinical trial"[Publication Type] OR "journal article"[Publication Type] OR "meta analysis"[Publication Type] OR "review"[Publication Type] OR "systematic review"[Publication Type])
PubMed	Cancer	((parks[tw] OR "natural environment*"[tw] OR "green space*"[tw] OR "greenspace*"[tw] OR "blue space*"[tw] OR "bluespace*"[tw] OR "greenness"[tw] OR "street tree*"[tw] OR "green exercise*"[tw] OR "urban tree*"[tw]) AND ("neoplasm*"[tw] OR "carcinoma"[tw] OR "cancer*"[tw] OR "lymphoma"[tw] OR "Hodgkin"[tw])) AND ("clinical trial"[Publication Type] OR "journal article"[Publication Type] OR "meta analysis"[Publication Type] OR "review"[Publication Type] OR "systematic review"[Publication Type])
PubMed	Diabetes	((parks[tw] OR "natural environment*"[tw] OR "green space*"[tw] OR "greenspace*"[tw] OR "blue space*"[tw] OR "bluespace*"[tw] OR "greenness"[tw] OR "street tree*"[tw] OR "green exercise*"[tw] OR "urban tree*"[tw]) AND ("diabet*"[tw] OR "insulin"[tw])) AND ("clinical trial"[Publication Type] OR "journal article"[Publication Type] OR "meta analysis"[Publication Type] OR "review"[Publication Type] OR "systematic review"[Publication Type])
PubMed	Atopic disease	((parks[tw] OR "natural environment*"[tw] OR "green space*"[tw] OR "greenspace*"[tw] OR "blue space*"[tw] OR "bluespace*"[tw] OR "greenness"[tw] OR "street tree*"[tw] OR "green exercise*"[tw] OR "urban tree*"[tw]) AND ("allergic respiratory disease"[tw] OR "asthma*"[tw] OR "wheez*"[tw] OR "hayfever"[tw] OR "lung function"[tw] OR "spirometry"[tw] OR "FEV1"[tw] OR "forced expiratory volume"[tw] OR "forced vital capacity"[tw] OR "atopy"[tw] OR "eczema"[tw] OR "dermatitis atopic"[tw] OR "neurodermatitis"[tw] OR "Besnier's prurigo"[tw] OR "respirat*"[tw])) AND ("clinical trial"[Publication Type] OR "journal article"[Publication Type] OR "meta analysis"[Publication Type] OR "review"[Publication Type] OR "systematic review"[Publication Type])
PubMed	Birth outcomes	((parks[tw] OR "natural environment*"[tw] OR "green space*"[tw] OR "greenspace*"[tw] OR "blue space*"[tw] OR "bluespace*"[tw] OR "greenness"[tw] OR "street tree*"[tw] OR "green exercise*"[tw] OR "urban tree*"[tw]) AND ("birth outcome"[tw] OR "preterm birth*"[tw] OR "low birth weight*"[tw] OR "low birthweight*"[tw] OR "intrauterine growth retardation"[tw] OR "small

Database	Health outcome	Search expression
		for gestational age"[tw] OR "pregnancy outcome*"[tw] OR "maternal outcome*"[tw] OR "reproductive outcome*"[tw] OR "preeclampsia"[tw] OR "gestational diabetes"[tw] OR "spontaneous abortion"[tw] OR "pregnancy loss*"[tw] OR "neonatal"[tw] OR "perinatal"[tw] OR "fetal"[tw] OR "fetus"[tw] OR "pregnancy"[tw] OR "embryonic"[tw] OR "infant"[tw] OR "maternal mortality"[tw] OR "postpartum"[tw])) AND ("clinical trial"[Publication Type] OR "journal article"[Publication Type] OR "meta analysis"[Publication Type] OR "review"[Publication Type] OR "systematic review"[Publication Type])
PubMed	General health	((parks[tw] OR "natural environment*"[tw] OR "green space*"[tw] OR "greenspace*"[tw] OR "blue space*"[tw] OR "bluespace*"[tw] OR "greenness"[tw] OR "street tree*"[tw] OR "green exercise*"[tw] OR "urban tree*"[tw]) AND ("physical health"[tw] OR "chronic condition*"[tw] OR "morbidit*"[tw] OR "self-reported health"[tw] OR "perceived health"[tw])) AND ("clinical trial"[Publication Type] OR "journal article"[Publication Type] OR "meta analysis"[Publication Type] OR "review"[Publication Type] OR "systematic review"[Publication Type])
Cochrane	Mortality	"parks" OR "natural environment*" OR "green space*" OR greenspace* OR "blue space*" OR bluespace* OR greenness OR "street tree*" OR "green exercise*" OR "urban tree*" in Title Abstract Keyword AND "mortalit*" OR "life expentanc*" in Title Abstract Keyword
Cochrane	Cardiovascular health/disease and obesity	"parks" OR "natural environment*" OR "green space*" OR greenspace* OR "blue space*" OR bluespace* OR greenness OR "street tree*" OR "green exercise*" OR "urban tree*" in Title Abstract Keyword AND "obes*" OR "overweight*" OR "BMI*" OR "adipos*" OR "cardiovascular*" OR "acute MI" OR "myocardial infarction*" OR "ischemic heart disease*" OR "sudden cardiac arrest*" OR "heart failure*" OR "acute coronary syndrome*" OR "cardiometabolic" OR "hypertension" OR "sudden cardiac death*" in Title Abstract Keyword
Cochrane	Cancer	"parks" OR "natural environment*" OR "green space*" OR greenspace* OR "blue space*" OR bluespace* OR greenness OR "street tree*" OR "green exercise*" OR "urban tree*" in Title Abstract Keyword AND "neoplasm*" OR "carcinoma" OR "cancer*" OR "lymphoma" OR "Hodgkin" in Title Abstract Keyword
Cochrane	Diabetes	"parks" OR "natural environment*" OR "green space*" OR greenspace* OR "blue space*" OR bluespace* OR greenness OR "street tree*" OR "green exercise*" OR "urban tree*" in Title Abstract Keyword AND "diabet*" OR "insulin" in Title Abstract Keyword
Cochrane	Atopic disease	"parks" OR "natural environment*" OR "green space*" OR greenspace* OR "blue space*" OR bluespace* OR greenness OR "street tree*" OR "green exercise*" OR "urban tree*" in Title Abstract Keyword AND "allergic respiratory disease*" OR "asthma*" OR "wheez*" OR "allergic rhin*" OR "hayfever" OR "lung function*" OR "spirometry" OR "FEV1" OR "forced expiratory volume*" OR "forced vital capacity*" OR "atopy" OR "eczema" OR "atopic dermatit*" OR (ige AND (sensitization OR sensitization)) OR "allerg*" OR "atopic dermatitis" OR "dermatitis atopic" OR "neurodermatitis" OR "Besnier's prurigo" OR "respirat*" in Title Abstract Keyword
Cochrane	Birth outcomes	"parks" OR "natural environment*" OR "green space*" OR greenspace* OR "blue space*" OR bluespace* OR greenness OR "street tree*" OR "green exercise*" OR "urban tree*" in Title Abstract Keyword AND "birth outcome*" OR "preterm birth*" OR "premature birth*" OR "low birth weight*" OR "low birthweight*" OR "intrauterine growth retardation" OR "small for gestational age" OR "pregnancy outcome*" OR "maternal outcome*" OR "reproductive outcome*" OR "preeclampsia" OR "gestational diabetes" OR "spontaneous abortion" OR "pregnancy loss*" OR "neonatal" OR "perinatal" OR "fetal" OR "fetus" OR "pregnancy" OR "embryonic" OR "infant" OR "maternal mortality" OR "postpartum" in Title Abstract Keyword
Cochrane	General health	"parks" OR "natural environment*" OR "green space*" OR greenspace* OR "blue space*" OR bluespace* OR greenness OR "street tree*" OR "green exercise*" OR "urban tree*" in Title Abstract Keyword AND "physical health" OR "chronic condition*" OR "*morbidit*" OR "self-reported health" OR "perceived health" in Title Abstract Keyword
Web of Science	Mortality	(TS=((parks OR "natural environment*" OR "green space*" OR "greenspace*" OR "blue space*" OR "bluespace*" OR "greenness" OR "street tree*" OR "green exercise*" OR "urban tree*") AND ("mortalit*" OR "life expentanc*"))) *AND* LANGUAGE: (English) *AND* DOCUMENT TYPES: (Article OR Review)
Web of Science	Cardiovascular health/disease and obesity	(TS=((parks OR "natural environment*" OR "green space*" OR "greenspace*" OR "blue space*" OR "bluespace*" OR "greenness" OR "street tree*" OR "green exercise*" OR "urban tree*") AND ("obes*" OR "overweight*" OR "BMI*" OR "adipos*" OR "cardiovascular*" OR "acute MI" OR "myocardial infarction*" OR "ischemic heart disease*" OR "sudden cardiac arrest*" OR "heart

Database	Health outcome	Search expression
		failure*" OR "acute coronary syndrome*" OR "cardiometabolic" OR "hypertension" OR "sudden cardiac death*"))) *AND* LANGUAGE: (English) *AND* DOCUMENT TYPES: (Article OR Review)
Web of Science	Cancer	(TS=((parks OR "natural environment*" OR "green space*" OR "greenspace*" OR "blue space*" OR "bluespace*" OR "greenness" OR "street tree*" OR "green exercise*" OR "urban tree*") AND ("neoplasm*" OR "carcinoma" OR "cancer*" OR "lymphoma" OR "Hodgkin"))) AND LANGUAGE: (English) AND DOCUMENT TYPES: (Article OR Review)
Web of Science	Diabetes	(TS=((parks OR "natural environment*" OR "green space*" OR "greenspace*" OR "blue space*" OR "bluespace*" OR "greenness" OR "street tree*" OR "green exercise*" OR "urban tree*") AND ("diabet*" OR "insulin"))) AND LANGUAGE: (English) AND DOCUMENT TYPES: (Article OR Review)
Web of Science	Atopic disease	(TS=((parks OR "natural environment*" OR "green space*" OR "greenspace*" OR "blue space*" OR "bluespace*" OR "greenness" OR "street tree*" OR "green exercise*" OR "urban tree*") AND ("allergic respiratory disease*" OR "asthma*" OR "wheez*" OR "allergic rhin*" OR "hayfever" OR "lung function*" OR "spirometry" OR "FEV1" OR "forced expiratory volume*" OR "forced vital capacity*" OR "atopy" OR "eczema" OR "atopic dermatit*" OR (ige AND (sensitization OR sensitization)) OR "allerg*" OR "atopic dermatitis" OR "dermatitis atopic" OR "neurodermatitis" OR "Besnier's prurigo" OR "respirat*"))) AND LANGUAGE: (English) AND DOCUMENT TYPES: (Article OR Review)
Web of Science	Birth outcomes	(TS=((parks OR "natural environment*" OR "green space*" OR "greenspace*" OR "blue space*" OR "bluespace*" OR "greenness" OR "street tree*" OR "green exercise*" OR "urban tree*") AND ("birth outcome*" OR "preterm birth*" OR "premature birth*" OR "low birth weight*" OR "low birthweight*" OR "intrauterine growth retardation" OR "small for gestational age" OR "pregnancy outcome*" OR "maternal outcome*" OR "reproductive outcome*" OR "preeclampsia" OR "gestational diabetes" OR "spontaneous abortion" OR "pregnancy loss*" OR "neonatal" OR "perinatal" OR "fetal" OR "fetus" OR "pregnancy" OR "embryonic" OR "infant" OR "maternal mortality" OR "postpartum"))) AND LANGUAGE: (English) AND DOCUMENT TYPES: (Article OR Review)
Web of Science	General health	(TS=((parks OR "natural environment*" OR "green space*" OR "greenspace*" OR "blue space*" OR "bluespace*" OR "greenness" OR "street tree*" OR "green exercise*" OR "urban tree*") AND ("physical health" OR "chronic condition*" OR "*morbidit*" OR "self-reported health" OR "perceived health"))) AND LANGUAGE: (English) AND DOCUMENT TYPES: (Article OR Review)
Scopus	Mortality	TITLE-ABS-KEY ((parks OR "natural environment*" OR "green space*" OR "greenspace*" OR "blue space*" OR "bluespace*" OR "greenness" OR "street tree*" OR "green exercise*" OR "urban tree*") AND ("mortalit*" OR "life expentanc*")) AND (LIMIT-TO (DOCTYPE , "ar") OR LIMIT-TO (DOCTYPE , "re"))
Scopus	Cardiovascular health/disease and obesity	TITLE-ABS-KEY ((parks OR "natural environment*" OR "green space*" OR "greenspace*" OR "blue space*" OR "bluespace*" OR "greenness" OR "street tree*" OR "green exercise*" OR "urban tree*") AND ("obes*" OR "overweight*" OR "BMI*" OR "adipos*" OR "cardiovascular*" OR "acute MI" OR "myocardial infarction*" OR "ischemic heart disease*" OR "sudden cardiac arrest*" OR "heart failure*" OR "acute coronary syndrome*" OR "cardiometabolic" OR "hypertension" OR "sudden cardiac death*")) AND (LIMIT-TO (DOCTYPE , "ar") OR LIMIT-TO (DOCTYPE , "re"))
Scopus	Cancer	TITLE-ABS-KEY ((parks OR "natural environment*" OR "green space*" OR "greenspace*" OR "blue space*" OR "bluespace*" OR "greenness" OR "street tree*" OR "green exercise*" OR "urban tree*") AND ("neoplasm*" OR "carcinoma" OR "cancer*" OR "lymphoma" OR "Hodgkin")) AND (LIMIT-TO (DOCTYPE , "ar") OR LIMIT-TO (DOCTYPE , "re"))
Scopus	Diabetes	TITLE-ABS-KEY ((parks OR "natural environment*" OR "green space*" OR "greenspace*" OR "blue space*" OR "bluespace*" OR "greenness" OR "street tree*" OR "green exercise*" OR "urban tree*") AND ("diabet*" OR "insulin")) AND (LIMIT-TO (DOCTYPE , "ar") OR LIMIT-TO (DOCTYPE , "re"))
Scopus	Atopic disease	TITLE-ABS-KEY ((parks OR "natural environment*" OR "green space*" OR "greenspace*" OR "blue space*" OR "bluespace*" OR "greenness" OR "street

Database	Health outcome	Search expression
		tree*" OR "green exercise*" OR "urban tree*") AND ("allergic respiratory disease*" OR "asthma*" OR "wheez*" OR "allergic rhin*" OR "hayfever" OR "lung function*" OR "spirometry" OR "FEV1" OR "forced expiratory volume*" OR "forced vital capacity*" OR "atopy" OR "eczema" OR "atopic dermatit*" OR (ige AND (sensitization OR sensitization)) OR "allerg*" OR "atopic dermatitis" OR "dermatitis atopic" OR "neurodermatitis" OR "Besnier's prurigo" OR "respirat*")) AND (LIMIT-TO (DOCTYPE , "ar") OR LIMIT-TO (DOCTYPE , "re"))
Scopus	Birth outcomes	TITLE-ABS-KEY ((parks OR "natural environment*" OR "green space*" OR "greenspace*" OR "blue space*" OR "bluespace*" OR "greenness" OR "street tree*" OR "green exercise*" OR "urban tree*") AND ("birth outcome*" OR "preterm birth*" OR "premature birth*" OR "low birth weight*" OR "low birthweight*" OR "intrauterine growth retardation" OR "small for gestational age" OR "pregnancy outcome*" OR "maternal outcome*" OR "reproductive outcome*" OR "preeclampsia" OR "gestational diabetes" OR "spontaneous abortion" OR "pregnancy loss*" OR "neonatal" OR "perinatal" OR "fetal" OR "fetus" OR "pregnancy" OR "embryonic" OR "infant" OR "maternal mortality" OR "postpartum")) AND (LIMIT-TO (DOCTYPE , "ar") OR LIMIT-TO (DOCTYPE , "re"))
Scopus	General health	TITLE-ABS-KEY ((parks OR "natural environment*" OR "green space*" OR "greenspace*" OR "blue space*" OR "bluespace*" OR "greenness" OR "street tree*" OR "green exercise*" OR "urban tree*") AND ("physical health" OR "chronic condition*" OR "*morbidit*" OR "self-reported health" OR "perceived health")) AND (LIMIT-TO (DOCTYPE , "ar") OR LIMIT-TO (DOCTYPE , "re"))

Note: We initially combined cardiovascular health/diseases with obesity-related measures, but then decided to separate the two.

Table S4. *Codes included in the data extraction sheet.*

Codes	Explanation
Author(s)	Authors of the article
Date	Year when it was published
Title	Title of the article
Journal	Journal in which the article was published
New_study	This is to note whether there are multiple rows (analyses) from the same study.
Continent	Continent where the study was conducted (does not refer to the nationality of the authors)
Country	Country where the study was conducted (does not refer to the nationality of the authors)
Design_experiment	Study was experimental or quasi-experimental with control group
Design_observational	Study was observational, no random assignment OR no control group
Design_cross_section	Study was cross-sectional, involved data for the DV at a single point in time.
Design_longitudinal	Study was longitudinal, involved data for the DV at multiple points in time.
Sample_n_individuals	Number of individuals (i.e., subjects) in the study, regardless of the unit of analysis. If it's an experiment, insert the total number of participants, excluding dropouts.
Sample_agelow	Minimum age of the participants in the study (e.g., write 25 if participants are aged 25-65). If not specified, it's likely to be adults, so write 18
Sample_agemeanmed	Median or mean age (whatever is reported) of the participants in the study. If both are reported, write the median. If not reported, write NR
Sample_agehigh	Maximum age of the participants in the study (e.g., write 65 if participants are aged 25-65). If not reported, write NR
Outcome	The health outcome studied for this particular row. Chose one of the following outcomes:<br>- Obesity-related: Anything related to obesity, BMI, weight, adiposity<br>- Cardiovascular health/disease: Cardiovascular disease events (e.g., stroke, CVD-mortality) and biomarkers (e.g., blood pressure)<br>- Mortality: Includes premature mortality, all-cause mortality, premature mortality, life expectancy, etc.<br>- Gen health: General health (perceived, self-reported)<br>- Cancer (e.g., incidence of diagnosis)<br>- Diabetes (e.g., fasting glucose, type 2 diabetes mellitus incidence/prevalence)<br>- Atopic diseases (e.g., asthma, allergic rhinitis, eczema)<br>- Birth outcomes (e.g., birthweight, premature birth)
Outcome_Spec	Specific health outcome (e.g., stroke, BMI, type of birth outcome). Open ended.
Green_Descr	Description of the green space (e.g., park, greenness, trees). Open ended.
GS_Type	Type of green space. Includes the following:<br>- Green land cover: Measures of vegetation (predominantly remotely sensed measures) including NDVI greenness, tree canopy, and others<br>- Public green space: Publicly accessible green space including parks, trails, and others<br>- Nature-based program: Recreational or physical activity programs based in parks, forests, or other green spaces.<br>- Garden: Residential gardens or yards
Green_Obj_Sub	Green space measured through objective (e.g., GIS, remote sensing) or subjective means (e.g., surveys)
Green_res_1m	Resolution of green space data 1 m (for papers using remote sensing only)
Green_res_2_to_30m	Resolution of green space data 2 to 30 m (for papers using remote sensing only)
Green_res_31m_to_249m	Resolution of green space data 31 to 249 m (for papers using remote sensing only)
Green_res_250m_or_more	Resolution of green space data 250 m or greater (for papers using remote sensing only)
Green_res_not reported	Resolution of green space data not reported (for papers using remote sensing only)
Green_seasons	Number of seasons for which green space data is collected (e.g., spring, summer, fall) (for papers using remote sensing only)
Green_Container	The GIS method follows the "container" approach - i.e., green is measured within a spatial units where residents live (e.g., census tract)
Green_DistBased	The GIS method follows a "distance-based" approach - i.e., green space is measured at a specified distance from each unit of analysis (e.g., individual, census tract). For example, a study measuring associations between green space within X miles of a person's house and a health outcome

Codes	Explanation
Green_dist	Only applies to Green_DistBased: Green space measurement around individual or unit of analysis. For example, a study measuring associations between green space within X meters of a person's house and a health outcome.
Green_dist_cat	Categories for green distance<br>- Group 1: 0-500m<br>- Group 2: 501-1,000m<br>- Group 3: 1,001-2,000m<br>- Group 4: Larger than 2,001m
Unit_hood	Unit of analysis was the neighborhood. If it's a multi-level model (e.g., individual within census tracts, report the lower level of the model - i.e., individual).
Unit_city	Unit of analysis was the city. If it's a multi-level model (e.g., individual within census tracts, report the lower level of the model - i.e., individual).
Unit_block	Unit of analysis was the block. If it's a multi-level model (e.g., individual within census tracts, report the lower level of the model - i.e., individual).
Unit_individ	Unit of analysis was the individual. If it's a multi-level model (e.g., individual within census tracts, report the lower level of the model - i.e., individual).
Unit_district	Unit of analysis was the district. If it's a multi-level model (e.g., individual within census tracts, report the lower level of the model - i.e., individual).
Unit_parcel	Unit of analysis was the parcel. If it's a multi-level model (e.g., individual within census tracts, report the lower level of the model - i.e., individual).
EM_SES	Potential effect modification by SES (income, poverty, educational level, other economic measures) between the green space and health outcome reported
EM_SES_type	Open-ended. Specific SES measure used for the effect modification - e.g., income, education, deprivation index.
EM_SES_sign	Sign of the effect modification of SES on the GBS-health relationship: 1 low-SES benefit most, 0 nobody benefits most, -1 high-SES benefit most, and in-between values (see main text); NA if not studied
EM_SES_analysis	1: Interaction, 0: split sample, NA: Not applicable
EM_Race	Potential effect modification by race and/or ethnicity between the green space and health outcome reported
EM_Race_sign	Sign of the effect modification of race/ethnicity on the GBS-health relationship: 1 racially/ethnically minoritized people benefit most, 0 nobody benefits most, -1 White people benefit most, and in-between values (see main text); NA if not studied
EM_Race_sign_group	Which specific group benefits over which other group - e.g., Latinx people have more beneficial effects from green space than White people
EM_Race_analysis	1: Interaction, 0: split sample, NA: Not applicable
**Bias Evaluation**	
Bias_Design	To what extent does the effect modification analysis reliably show a robust relationship between the outcome and green space measure(s) - SELECT ONE OF THE FOLLOWING<br>Design - Unit of analysis - greenspace geography - Outcome measure time<br>- Experiment - Individual - NA - NA = 5.0<br>- Quasi-experiment - Individual - NA - NA = 4.0<br>- Observational - Individual - Residential address - Multiple years = 3.5<br>- Observational - Individual - Residential address - Single year = 3.0<br>- Observational - Individual - Tract or district - Multiple years = 2.5<br>- Observational - Individual - Tract or district - Single year = 2.0<br>- Observational - Tract or district - Tract or district - Multiple years = 1.5<br>- Observational - Tract or district - Tract or district - Single year = 1.0
Bias_Exposure	Does the green measure(s) reliably distinguish between levels of exposure in a time window considered most relevant for a causal effect with respect to the development of the outcome? COUNT ALL THAT APPLY AND SUM THE POINTS<br>*For all studies:*<br>- Reasonable alignment (± 5 years) between year(s) in which exposure and outcome variables were measured +2.0<br>*For GIS-based studies with objective measures of green space:*<br>- Exposure levels calculated with high-resolution remote sensing data (e.g. approximately 1 m) – for GIS-based studies with objective measures of green space +2.0<br>- Exposure levels calculated with moderate-resolution remote sensing data (e.g. 20 to 50 m), and

Codes	Explanation
	not higher-resolution data – for GIS-based studies with objective measures of green space +1.0<br>- Exposure levels calculated with low-resolution remote sensing data (e.g. 250 m or more), and not higher-resolution data – for GIS-based studies with objective measures of green space +0.0<br>*For survey-based studies (subjective measures of green space):*<br>- Perceived access to green space measured through a valid and reliable survey (the authors mention that the survey instrument is valid and reliable, providing evidence) – for studies based on surveys and green space perceptions +2.0<br>- Perceived access to green space measured through a field-tested survey (the authors mention that the survey instrument is field-tested, but do not mention validity or reliability) – for studies based on surveys and green space perceptions +1.0<br>- Perceived access to green space measured through a survey, but the authors do not mention whether it's field tested or valid/reliable – for studies based on surveys and green space perceptions + 0.0<br>[Total possible: 4.0 for remote sensing studies and survey-based studies, 2.0 for other designs (e.g., GIS studies for which green space polygons are based on property records (resolution not available) and nature-based programs).]
Bias_Confounding	Is confounding of the effect of the exposure likely? COUNT ALL THAT APPLY AND SUM THE POINTS<br>- Rationale for selection and inclusion of control variables based on empirical data (e.g., gained through acyclic graphing or bivariate correlations) +1.0<br>- Rationale for selection and inclusion of control variables based on theory or thoughtful review of published literature +1.0<br>- Does not include variables in the models that have been shown to be influential colliders or intermediates on the causal pathway in at least one model (e.g., physical activity, air pollution) +1.0<br>- For observational studies, adequate control for confounding variables, specifically socioeconomic status (SES) [Does not apply to experimental or quasi-experimental designs] +2.0<br>[Total possible: 3 for experimental studies, 5 for observational studies]
Bias_Analysis	Does the analysis strategy and presentation convey the necessary familiarity with the data and assumptions? COUNT ALL THAT APPLY AND SUM THE POINTS<br>- Appropriate analysis for the study design and research question, such as a detailed description of the statistical technique used, explanation why this technique was chosen, and discussion of caveats regarding the conclusions drawn from analyses using this technique +2.0<br>- The authors report that model assumptions were tested and met +1.0<br>- Includes additional analyses addressing potential biases or limitations (i.e., sensitivity analyses) +1.0<br>- For geospatial analyses, controls for spatial autocorrelation +1.0<br>[Total possible: 5 for geospatial designs, 4 for other study designs]
Bias_Total	We will sum up the values in the bias columns
Bias_Total_Possible_Points	Total possible points for this analysis given the study design
Bias_Total_Score	Total as calculated above divided by the total number of possible points (varies between 0 and 1). The total number of possible points varies by study characteristics (see above rows)

Table S5. *Points assigned to each bias category for the articles included in the review*

Authors and date	Design	Exposure	Confounding	Analysis	Percent Possible Points	Quality
(Abelt & McLafferty, 2017)	2	3	3	2	53%	Fair
(Agay-Shay et al., 2014)	3	4	3	4	74%	Good
(Agay-Shay et al., 2019)	3	3	4	2	63%	Good
(Agyemang et al., 2007)	2	2	3	2	47%	Fair
(Alexander, Huber, Piper, & Tanner, 2013)	3	4	4	3	78%	Good
(Astell-Burt, Feng, & Kolt, 2014)	3	2	4	3	71%	Good
(Björk et al., 2008)	3	2	4	2	65%	Good
(Brindley, Jorgensen, & Maheswaran, 2018)	1	2	3	3	53%	Fair
(Brown et al., 2016)	2	3	3	2	53%	Fair
(Browning & Rigolon, 2018)	1	3	5	5	74%	Good
(Casey, James, Rudolph, Wu, & Schwartz, 2016)	3	2	4	5	74%	Good
(Coppel & Wüstemann, 2017)	3	2	3	2	59%	Fair
(Crouse et al., 2017)	3	2	2	2	47%	Fair
(Cummins & Fagg, 2012)	2	2	3	2	53%	Fair
(Cusack, Larkin, Carozza, & Hystad, 2017a)	3	3	3	4	68%	Good
(Cusack, Larkin, Carozza, & Hystad, 2017b)	3	2	2	4	58%	Fair
(Cusack et al., 2018)	3	3	3	3	63%	Good
(da Silveira & Junger, 2018)	1	3	4	3	58%	Fair
(Dadvand, de Nazelle, et al., 2012)	3	3.5	2	2	56%	Fair
(Dadvand, Sunyer, et al., 2012)	3	3	3	2	58%	Fair
(Dadvand, Villanueva, et al., 2014)	3	3	3	3	63%	Good
(Dadvand, Wright, et al., 2014)	3	3	4	4	74%	Good
(Dadvand et al., 2018)	3	4	3	2	63%	Good
(D’Agostino, Patel, Ahmed, et al., 2018)	3.5	2	4	3	74%	Good
(D’Agostino, Patel, Hansen, et al., 2018)	3.5	2	3	3	72%	Good
(Dalton et al., 2016)	3.5	3	3	3	66%	Good
(de Keijzer et al., 2017)	3	3	4	4	74%	Good
(de Vries, Verheij, Groenewegen, & Spreeuwenberg, 2003)	2.5	2.5	3	2	56%	Fair
(Demoury et al., 2017)	3	3	2	2	53%	Fair
(Donovan et al., 2013)	4	3	3	3	76%	Good

Authors and date	Design	Exposure	Confounding	Analysis	Percent Possible Points	Quality
(Donovan, Gatziolis, Longley, & Douwes, 2018)	3.5	3	4	3	71%	Good
(Dzhambov, Markevych, & Lercher, 2018)	3	3	5	4	79%	Good
(Ebisu, Holford, & Bell, 2016)	3	3	4	3	68%	Good
(Egorov et al., 2017)	3	4	4	4	79%	Good
(Eldeirawi et al., 2019)	3	3	4	3	68%	Good
(Fan & Jin, 2014)	3	4	4	3	78%	Good
(Fong et al., 2018)	3	3	2	3	58%	Fair
(Foster & Weinstein, 2019)	3	4	3	2	67%	Good
(Gidlow et al., 2016)	1	2	4	4	65%	Good
(Glazer et al., 2018)	3	3	4	3	68%	Good
(Groenewegen et al., 2018)	2	3	4	2	58%	Fair
(Hobbs et al., 2017)	2	3	2	4	58%	Fair
(Hobbs et al., 2018)	2	2	3	2	53%	Fair
(Hughey et al., 2017)	2	2	3	3	59%	Fair
(Hystad et al., 2014)	2	4	4	5	79%	Good
(James, Hart, Banay, & Laden, 2016)	3	2	2	3	53%	Fair
(Ji et al., 2019)	3	2	2	3	53%	Fair
(Jilcott Pitts et al., 2013)	1	2	3	3	53%	Fair
(Kihal-Talantikite et al., 2013)	2	0	3	2	41%	Fair
(Kling et al., 2018)	3.5	2	4	2	72%	Good
(Lachowycz & Jones, 2014)	1	2	4	3	59%	Fair
(Lovasi et al., 2012)	3	2	3	2	59%	Fair
(Maas, Verheij, Groenewegen, De Vries, & Spreeuwenberg, 2006)	2	3	4	3	63%	Good
(Maas et al., 2009)	2	3	3	2	53%	Fair
(Markevych et al., 2014)	3	3	3	1	53%	Fair
(Mena, Fuentes, Ormazábal, Palomo-Vélez, & Palomo, 2015)	3	2	0	0	29%	Poor
(Mitchell & Popham, 2008)	2	3	3	3	58%	Fair
(Mueller et al., 2018)	2	2	3	3	59%	Fair
(Nichani et al., 2017)	2	2	3	3	59%	Fair
(Nieuwenhuijsen et al., 2018)	2	3	3	3	58%	Fair
(Orioli et al., 2019)	3	3	4	4	74%	Good
(Persson et al., 2018)	3.5	3	4	4	76%	Good
(Petraviciene, Grazuleviciene, Andrusaityte, Dedele, & Nieuwenhuijsen, 2018)	3	3	4	2	63%	Good

Authors and date	Design	Exposure	Confounding	Analysis	Percent Possible Points	Quality
(Pun, Manjourides, & Suh, 2018)	3.5	2	3	3	61%	Good
(Reid, Clougherty, Shmool, & Kubzansky, 2017)	3	4	3	4	74%	Good
(Richardson, Shortt, Mitchell, & Pearce, 2018)	2	4	3	4	68%	Good
(Rossi et al., 2018)	3	2	3	3	61%	Good
(Rossi, De Fragas, Corrêa, Das Neves, & De Assis Guedes De Vasconcelos, 2019)	3	2	3	3	58%	Fair
(Ruijsbroek et al., 2017)	2	2	3	2	47%	Fair
(Sarkar, 2017)	3	4	2	3	63%	Good
(Schalkwijk, Van Der Zwaard, Nijpels, Elders, & Platt, 2018)	2.5	2	3	2	56%	Fair
(Schuler & O'Reilly, 2017)	3	4	4	3	78%	Good
(Seo, Choi, Kim, Kim, & Park, 2019)	2.5	2	3	2	56%	Fair
(Singh, Siahpush, & Kogan, 2010)	3	4	3	2	67%	Good
(Sullivan, Brashear, Broyles, & Rung, 2014)	3	4	3	3	72%	Good
(Thiering et al., 2016)	3	3	3	4	68%	Good
(Triguero-Mas et al., 2015)	3	2.5	4	3	70%	Good
(Van Der Zwaard, Schalkwijk, Elders, Platt, & Nijpels, 2018)	2.5	2	4	4	74%	Good
(Vienneau et al., 2017)	3	3	3	4	68%	Good
(Villeneuve et al., 2012)	2	3	4	2	58%	Fair
(Villeneuve, Jerrett, Su, Weichenthal, & Sandler, 2018)	3	3	4	3	68%	Good
(Q. Wang & Lan, 2019)	1	2	4	4	65%	Good
(M. Wen & Kowaleski-Jones, 2012)	2	2	3	3	59%	Fair
(Wilker et al., 2014)	3	2	3	3	58%	Fair
(Wu et al., 2018)	1	2.5	5	5	75%	Good
(Xu, Ren, Yuan, Nichol, & Goggins, 2017)	1	3	3	2	47%	Fair
(Yang, Markevych, Bloom, et al., 2019)	3	3	5	4	79%	Good
(Yang, Markevych, Heinrich, et al., 2019)	3	3	4	4	74%	Good
(Yeager et al., 2018)	3	2	4	4	68%	Good
(Yitshak-Sade et al., 2019)	3	2	2	3	53%	Fair

Table S6. *Dunn's post-hoc pairwise tests for the Effect Modification (EM) values.*

EM	Comparison	Z	*p* value
**EM by SES: Green space type**	Gardens - Green land cover	1.088	0.276
	Gardens - Nature-based programs	0.000	1.000
	Green land cover - Nature-based programs	-1.088	0.276
	Gardens - Public green space	0.098	0.921
	Green land cover - Public green space	-2.511	0.012
	Nature-based programs - Public green space	0.098	0.921
**EM by SES: Green space distance**	0-500m - 501-1,000m	-3.001	0.002
	0-500m - 1,001-2,000m	-2.422	0.015
	501-1,000m - 1,001-2,000m	-0.622	0.533
	0-500m - 2000m +	-3.246	0.001
	501-1,000m - 2000m +	-1.377	0.168
	1,001-2,000m - 2000m +	-0.605	0.545
**EM by SES: Health outcome type**	Cancer - CVD	-0.586	0.557
	Cancer – Diabetes	-0.994	0.320
	CVD – Diabetes	-0.907	0.364
	Cancer - Gen Health	-1.352	0.176
	CVD - Gen Health	-1.845	0.065
	Diabetes - Gen Health	-0.603	0.545
	Cancer - Birth outcome	-0.250	0.802
	CVD - Birth outcome	0.840	0.400
	Diabetes - Birth outcome	1.534	0.124
	Gen Health - Birth outcome	2.557	0.010
	Cancer - Mortality	-0.553	0.579
	CVD - Mortality	0.013	0.989
	Diabetes - Mortality	0.788	0.430
	Gen Health - Mortality	0.130	0.913
	Birth outcome - Mortality	-0.651	0.514
	Cancer - Obesity-related	-0.856	0.391
	CVD - Obesity-related	-0.698	0.484
	Diabetes - Obesity-related	0.371	0.710
	Gen Health - Obesity-related	1.200	0.229
	Birth outcome - Obesity-related	-1.508	0.131
	Mortality - Obesity-related	-0.561	0.574
	Cancer - Atopic disease	-0.747	0.455
	CVD - Atopic disease	-0.396	0.691
	Diabetes - Atopic disease	0.448	0.653
	Gen Health - Atopic disease	1.128	0.259
	Birth outcome - Atopic disease	-1.054	0.291
	Mortality - Atopic disease	-0.348	0.727
	Obesity-related - Atopic disease	0.154	0.877
**EM by SES: Continent**	Asia – Europe	-1.639	0.101
	Asia - North America	0.601	0.547

EM	Comparison	Z	*p* value
	Europe - North America	3.893	<0.001
	Asia - Oceania	-0.644	0.519
	Europe - Oceania	0.229	0.818
	North America - Oceania	-1.063	0.287
	Asia - South America	-0.540	0.589
	Europe - South America	0.464	0.641
	North America - South America	-1.015	0.310
	Oceania - South America	0.136	0.891
**EM by Race/Ethnicity: Continent**	Europe - North America	1.909	0.056
	Europe - Oceania	1.786	0.074
	North America - Oceania	0.700	0.483

Notes: EM: Effect modification. CVD: cardiovascular health/disease. Gen health: General health. Rows highlighted in light blue have $p < 0.10$. All other Dunn's post-hoc tests for EM race are not significant (by green space type, by green space distance, and by health outcome type).

Table S7. *First Sensitivity analysis: Summary of statistical tests conducted. EM values were recoded as integers (-1, 0, or 1).*

**Research Question 1**	
EM SES	Mean = 0.303
EM Race/ethnicity	Mean = 0.133
**Research Question 2**	
EM SES by green space type	Fisher's exact test, $p = 0.131$
EM Race/ethnicity by green space type	Fisher's exact test, $p = 0.331$
EM SES by green space distance	Fisher's exact test, $p = 0.008$
EM Race/ethnicity by green space distance	Fisher's exact test, $p = 0.264$
**Research Question 3**	
EM SES by health outcome type	Fisher's exact test, $p = 0.352$
EM Race/ethnicity by health outcome type	Fisher's exact test, $p = 0.366$
**Research Question 4**	
EM SES by continent	Fisher's exact test, $p = 0.006$
EM Race/ethnicity by continent	Fisher's exact test, $p = 0.455$

Table S8. *First Sensitivity analysis: Post-hoc pairwise comparisons for Fisher's exact tests for the Effect Modification (EM) values. EM values were recoded as integers (-1, 0, or 1).*

EM	Comparison	*p* Fisher value
**EM by SES: Green space type**	Gardens - Green land cover	0.683
	Gardens - Nature-based programs	1.000
	Green land cover - Nature-based programs	0.683
	Gardens - Public green space	1.000
	Green land cover - Public green space	0.031
	Nature-based programs - Public green space	1.000
**EM by SES: Green space distance**	0-500m - 501-1,000m	0.011
	0-500m - 1,001-2,000m	0.310
	501-1,000m - 1,001-2,000m	1.000
	0-500m - 2000m +	0.031
	501-1,000m - 2000m +	0.278
	1,001-2,000m - 2000m +	1.000
**EM by SES: Health outcome type**	Cancer - CVD	0.171
	Cancer - Diabetes	0.318
	CVD - Diabetes	0.793
	Cancer - Gen Health	0.228
	CVD - Gen Health	0.278
	Diabetes - Gen Health	1.000
	Cancer - Birth outcome	0.152
	CVD - Birth outcome	0.813
	Diabetes - Birth outcome	0.602
	Gen Health - Birth outcome	0.168
	Cancer - Mortality	0.154
	CVD - Mortality	0.599
	Diabetes - Mortality	0.513
	Gen Health - Mortality	0.207
	Birth outcome - Mortality	1.000
	Cancer - Obesity-related	0.560
	CVD - Obesity-related	0.355
	Diabetes - Obesity-related	0.749
	Gen Health - Obesity-related	0.552
	Birth outcome - Obesity-related	0.097
	Mortality - Obesity-related	0.089
	Cancer - Atopic disease	0.551
	CVD - Atopic disease	0.514
	Diabetes - Atopic disease	0.817
	Gen Health - Atopic disease	0.604
	Birth outcome - Atopic disease	0.227
	Mortality - Atopic disease	0.312
	Obesity-related - Atopic disease	1.000
**EM by SES: Continent**	Asia – Europe	0.115

EM	Comparison	*p* Fisher value
	Asia - North America	0.623
	Europe - North America	<0.001
	Asia - Oceania	0.748
	Europe - Oceania	1.000
	North America - Oceania	0.459
	Asia - South America	1.000
	Europe - South America	0.382
	North America - South America	0.809
	Oceania - South America	1.000
**EM by Race/Ethnicity: Continent**	Europe - North America	0.498
	Europe - Oceania	0.200
	North America - Oceania	0.729

Notes: EM: Effect modification. CVD: cardiovascular health/disease. Gen health: General health. Rows highlighted in light blue have $p < 0.10$. All other Dunn's post-hoc tests for EM race/ethnicity are not significant (by green space type, by green space distance, and by health outcome type).

Table S9. *Second sensitivity analysis (exclude nature-based programs): Means for EM values classified by green space type, green space distance, health outcome type, and continent*

	EM SES		EM Race/ethnicity	
	Mean (SD)	n	Mean (SD)	n
All rows	0.255 (0.603)	118	0.033 (0.617)	28
Green space type				
Gardens	0.500 (0.577)	4		
Green land cover	0.154 (0.594)	78	-0.007 (0.644)	20
Public green space	0.447 (0.585)	36	0.135 (0.570)	8
Green space distance				
0 - 500 m	-0.009 (0.529)	52	-0.060 (0.584)	15
501 - 1 000m	0.518 (0.464)	14	1.000 (0.000)	1
1001 - 2000 m	0.750 (0.500)	4	1.000 (0.000)	2
> 2000 m	1.000 (0.000)	4		
Health outcome type				
Atopic disease	0.273 (0.606)	11	-0.250 (0.000)	1
Birth outcomes	0.072 (0.466)	22	-0.036 (0.506)	11
Cancer	0.000 (0.000)	2		
Cardiovascular health/disease	0.193 (0.598)	23	0.166 (0.816)	6
Diabetes	0.425 (0.472)	10		
General health	0.559 (0.519)	17		
Mortality	0.091 (0.943)	11	0.500 (0.707)	2
Obesity-related	0.288 (0.619)	22	-0.052 (0.671)	8
Continent				
Asia	0.158 (0.609)	10		
Europe	0.448 (0.564)	61	0.625 (0.478)	4
North America	-0.026 (0.542)	40	-0.060 (0.614)	22
Oceania	0.416 (0.382)	3	-0.125 (0.176)	2
South America	0.250 (0.957)	4		

Note: SD represents standard deviation. n represents the number of rows in which the effect modification was tested.

Table S10. *Second sensitivity analysis (exclude nature-based programs): Kruskal-Wallis tests for the Effect Modification (EM) values.*

EM	χ^2	df	*p* value
EM by SES: Green space type	6.889	2	0.032
EM by SES: Green space distance	21.051	3	<0.001
EM by SES: Health outcome type	8.409	7	0.298
EM by SES: Continent	18.047	4	0.001
EM by Race/Ethnicity: Green space type *	62		0.352
EM by Race/Ethnicity: Green space distance	4.250	2	0.119
EM by Race/Ethnicity: Health outcome type	2.246	4	0.691
EM by Race/Ethnicity: Continent	4.703	2	0.119

Notes: EM: Effect modification. CVD: cardiovascular health/disease. Gen health: General health. * Wilcoxon rank sum test instead of Kruskal-Wallis because there were only two green space types

Table S11. *Second sensitivity analysis (exclude nature-based programs): Dunn's post-hoc pairwise tests for the Effect Modification (EM) values.*

EM	Comparison	Z	*p* value
**EM by SES: Green space type**	Green land cover - Public green space	-2.506	0.036
**EM by SES: Green space distance**	0-500m - 501-1,000m	-3.001	0.002
	0-500m - 1,001-2,000m	-2.422	0.015
	0-500m - 2000m +	-3.246	0.001
**EM by SES: Health outcome type**	CVD - Gen Health	-1.944	0.052
	Gen Health - Birth outcome	2.557	0.010
**EM by SES: Continent**	Europe - North America	4.172	<0.001
**EM by Race/Ethnicity: Continent**	Europe - North America	2.018	0.043
	Europe - Oceania	1.744	0.081

Notes: EM: Effect modification. CVD: cardiovascular health/disease. Gen health: General health. Only pairwise comparisons for which $p < 0.10$ are reported. All other non-reported pairwise comparisons had $p > 0.10$.

Table S12. *Articles in which (a) the green space-health relationship was null or negative and (b) reported effect modification tests for such relationship.*

Authors and date	Green space-health findings	Health outcome type	EM test focus	EM findings
Björk et al. (2018)	Null	General health	SES	No EM reported
Gidlow et al. (2016)	Null	CVD	SES	No EM reported
Jendrossek et al. (2017)	Null	CVD	SES	No EM reported
Maas et al. (2009)	Null	Cancer	SES	No EM reported
Ness et al. (2012)	Null	Obesity-related	Race/ethnicity	No EM reported
Ogneva-Himmelberger et al. (2015)	Null	Birth outcomes	Race/ethnicity	No EM reported
Potestio et al. (2009)	Null	Obesity-related	SES	No EM reported

Notes: Notes: EM = effect modification, SES = socioeconomic status, CVD = cardiovascular health/disease.

References

Björk, J., Albin, M., Grahn, P., Jacobsson, H., Ardö, J., Wadbro, J., Ostergren, P.O., 2008. Recreational values of the natural environment in relation to neighbourhood satisfaction, physical activity, obesity and wellbeing. Journal of Epidemiology and Community Health 62, 1–7. https://doi.org/10.1136/jech.2007.062414

Gidlow, C.J., Smith, G., Martinez, D., Wilson, R., Trinder, P., Gražulevičiene, R., Nieuwenhuijsen, M.J., 2016. Research note: Natural environments and prescribing in England. Landscape and Urban Planning 151, 103–108. https://doi.org/10.1016/j.landurbplan.2016.02.002

Jendrossek, M., Standl, M., Koletzko, S., Lehmann, I., Bauer, C.P., Schikowski, T., von Berg, A., Berdel, D., Heinrich, J., Markevych, I., 2017. Residential air pollution, road traffic, greenness and maternal hypertension: Results from GINIplus and LISAplus. International Journal of Occupational and Environmental Medicine 8, 131–142. https://doi.org/10.15171/ijoem.2017.1073

Maas, J., Verheij, R.A., De Vries, S., Spreeuwenberg, P., Schellevis, F.G., Groenewegen, P.P., 2009. Morbidity is related to a green living environment. Journal of Epidemiology and Community Health 63, 967–973. https://doi.org/10.1136/jech.2008.079038

Ness, M., Barradas, D.T., Irving, J., Manning, S.E., 2015. Correlates of overweight and obesity among American Indian/Alaska Native and Non-Hispanic White children and adolescents: National survey of children's health, 2007. Maternal and Child Health Journal 16, 268–277. https://doi.org/10.1007/s10995-012-1191-8

Ogneva-Himmelberger, Y., Dahlberg, T., Kelly, K., Moore Simas, T.A., 2015. Using geographic information science to explore associations between air pollution, environmental amenities, and preterm births. AIMS Public Health 2, 469–486. https://doi.org/10.3934/publichealth.2015.3.469

Potestio, M.L., Patel, A.B., Powell, C.D., McNeil, D.A., Jacobson, R.D., McLaren, L., 2009. Is there an association between spatial access to parks/green space and childhood overweight/obesity in Calgary, Canada? International Journal of Behavioral Nutrition and Physical Activity 6, 77. https://doi.org/10.1186/1479-5868-6-77

Table S13. *PRISMA Checklist*

**Section/topic**	**#**	**Checklist item**	**Reported on page #**
**TITLE**			
Title	1	Identify the report as a systematic review, meta-analysis, or both.	1
**ABSTRACT**			
Structured summary	2	Provide a structured summary including, as applicable: background; objectives; data sources; study eligibility criteria, participants, and interventions; study appraisal and synthesis methods; results; limitations; conclusions and implications of key findings; systematic review registration number.	1
**INTRODUCTION**			
Rationale	3	Describe the rationale for the review in the context of what is already known.	1-2
Objectives	4	Provide an explicit statement of questions being addressed with reference to participants, interventions, comparisons, outcomes, and study design (PICOS).	2
**METHODS**			
Protocol and registration	5	Indicate if a review protocol exists, if and where it can be accessed (e.g., Web address), and, if available, provide registration information including registration number.	Suppl. Materials
Eligibility criteria	6	Specify study characteristics (e.g., PICOS, length of follow-up) and report characteristics (e.g., years considered, language, publication status) used as criteria for eligibility, giving rationale.	6
Information sources	7	Describe all information sources (e.g., databases with dates of coverage, contact with study authors to identify additional studies) in the search and date last searched.	5-6
Search	8	Present full electronic search strategy for at least one database, including any limits used, such that it could be repeated.	Suppl. Materials
Study selection	9	State the process for selecting studies (i.e., screening, eligibility, included in systematic review, and, if applicable, included in the meta-analysis).	6
Data collection process	10	Describe method of data extraction from reports (e.g., piloted forms, independently, in duplicate) and any processes for obtaining and confirming data from investigators.	7-9
Data items	11	List and define all variables for which data were sought (e.g., PICOS, funding sources) and any assumptions and simplifications made.	N/A
Risk of bias in individual studies	12	Describe methods used for assessing risk of bias of individual studies (including specification of whether this was done at the study or outcome level), and how this information is to be used in any data synthesis.	9
Summary measures	13	State the principal summary measures (e.g., risk ratio, difference in means).	8-9

Section/topic	#	Checklist item	Reported on page #
Synthesis of results	14	Describe the methods of handling data and combining results of studies, if done, including measures of consistency (e.g., I^2) for each meta-analysis.	9-10
Risk of bias across studies	15	Specify any assessment of risk of bias that may affect the cumulative evidence (e.g., publication bias, selective reporting within studies).	9
Additional analyses	16	Describe methods of additional analyses (e.g., sensitivity or subgroup analyses, meta-regression), if done, indicating which were pre-specified.	10
**RESULTS**			
Study selection	17	Give numbers of studies screened, assessed for eligibility, and included in the review, with reasons for exclusions at each stage, ideally with a flow diagram.	7
Study characteristics	18	For each study, present characteristics for which data were extracted (e.g., study size, PICOS, follow-up period) and provide the citations.	12-15, Suppl. Materials
Risk of bias within studies	19	Present data on risk of bias of each study and, if available, any outcome level assessment (see item 12).	15
Results of individual studies	20	For all outcomes considered (benefits or harms), present, for each study: (a) simple summary data for each intervention group (b) effect estimates and confidence intervals, ideally with a forest plot.	Suppl. Materials
Synthesis of results	21	Present results of each meta-analysis done, including confidence intervals and measures of consistency.	15-18
Risk of bias across studies	22	Present results of any assessment of risk of bias across studies (see Item 15).	15
Additional analysis	23	Give results of additional analyses, if done (e.g., sensitivity or subgroup analyses, meta-regression [see Item 16]).	15-18
**DISCUSSION**			
Summary of evidence	24	Summarize the main findings including the strength of evidence for each main outcome; consider their relevance to key groups (e.g., healthcare providers, users, and policy makers).	18-20
Limitations	25	Discuss limitations at study and outcome level (e.g., risk of bias), and at review-level (e.g., incomplete retrieval of identified research, reporting bias).	20-21
Conclusions	26	Provide a general interpretation of the results in the context of other evidence, and implications for future research.	21
**FUNDING**			
Funding	27	Describe sources of funding for the systematic review and other support (e.g., supply of data); role of funders for the systematic review.	N/A

Notes: Table columns and description from: Moher D, Liberati A, Tetzlaff J, Altman DG, The PRISMA Group (2009). Preferred Reporting Items for Systematic Reviews and Meta-Analyses: The PRISMA Statement. PLoS Med 6(7): e1000097. doi:10.1371/journal.pmed1000097

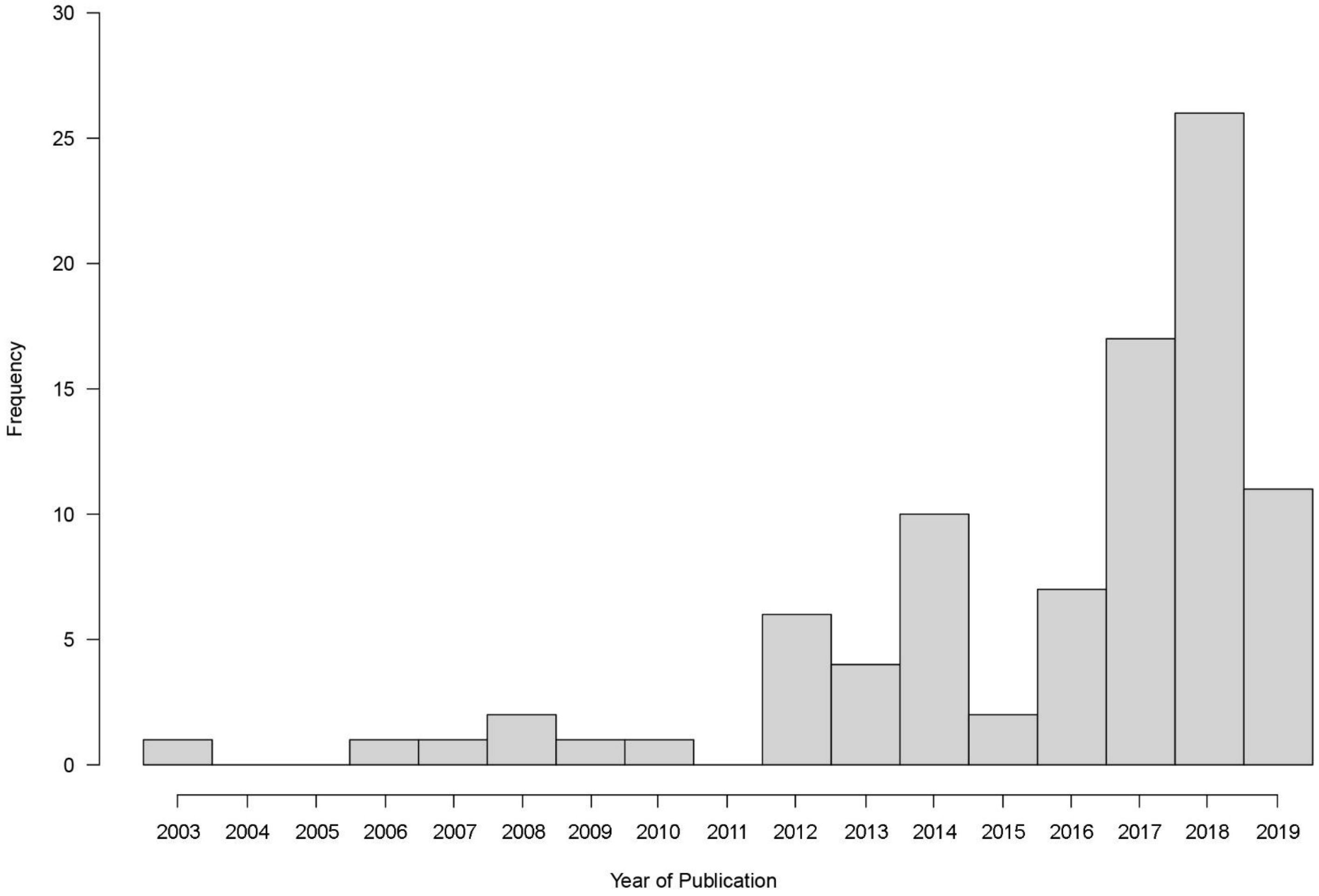

Figure S1. Year of publication of the 90 articles included in this review.

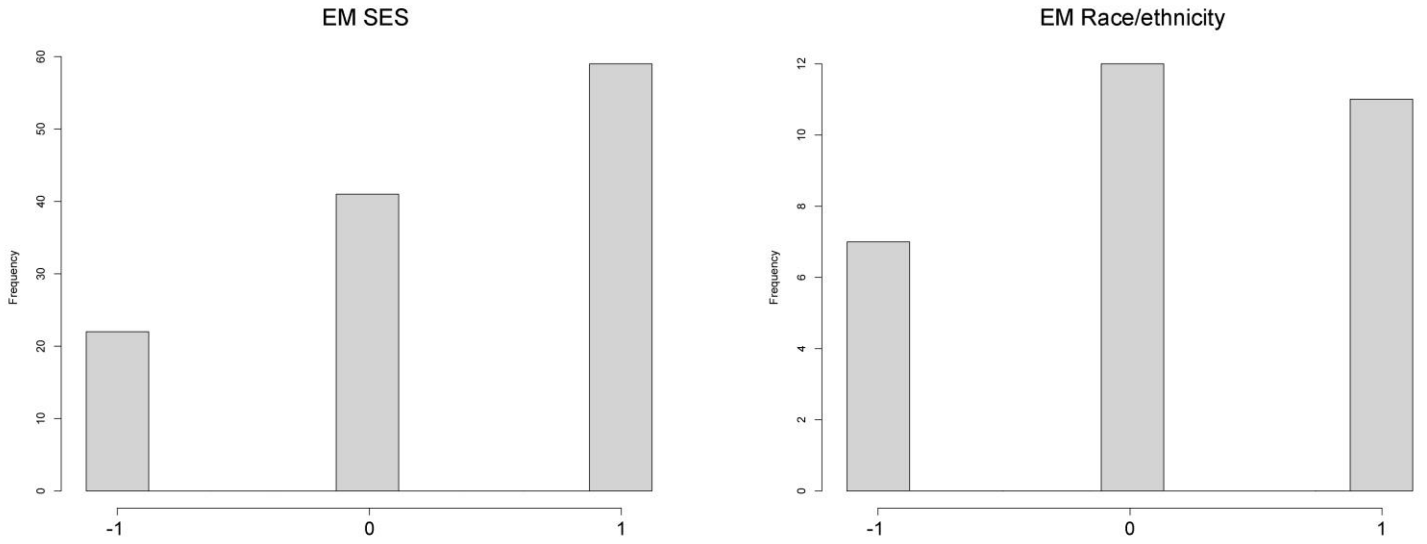

Figure S2. First sensitivity analysis: Effect modification (EM) for SES and Race/ethnicity. EM was recoded as -1, 0, or 1.

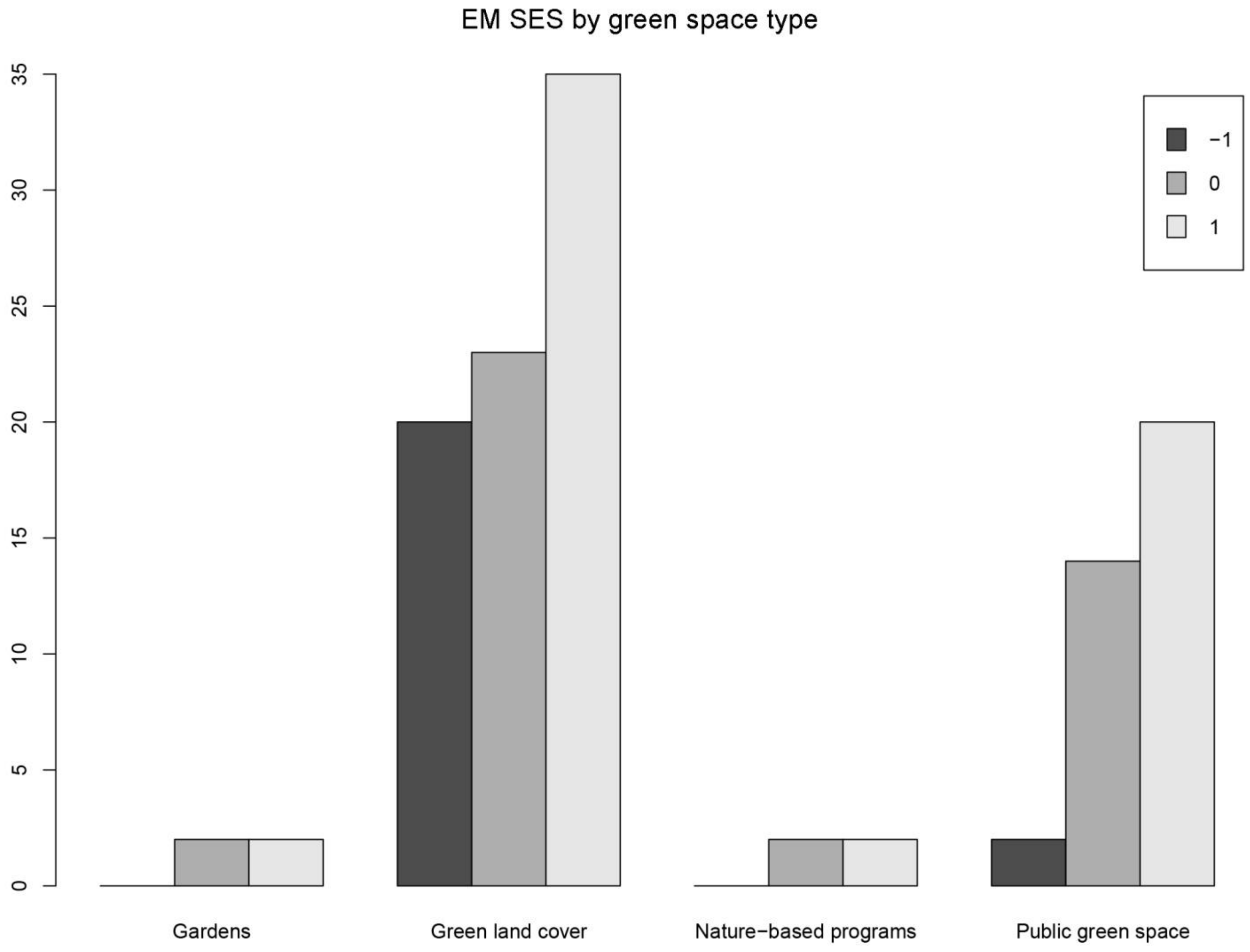

Figure S3. First sensitivity analysis: Effect modification (EM) for SES classified by green space type. EM was recoded as -1 (high-SES people benefit more), 0 (no differences), or 1 (low-SES people benefit more).

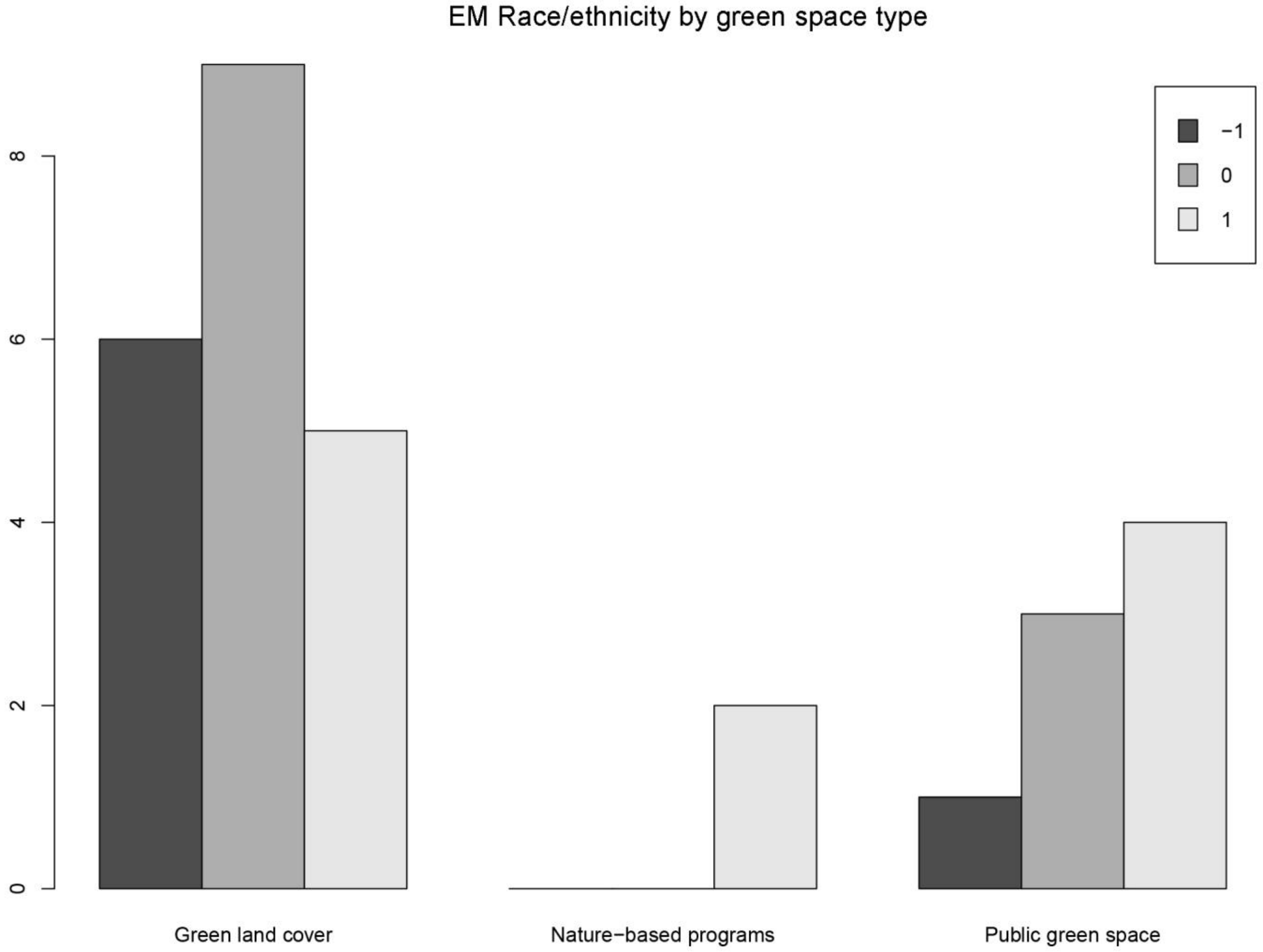

Figure S4. First sensitivity analysis: Effect modification (EM) for race/ethnicity classified by green space type. EM was recoded as -1 (White people benefit more), 0 (no differences), or 1 (non-White people benefit more).

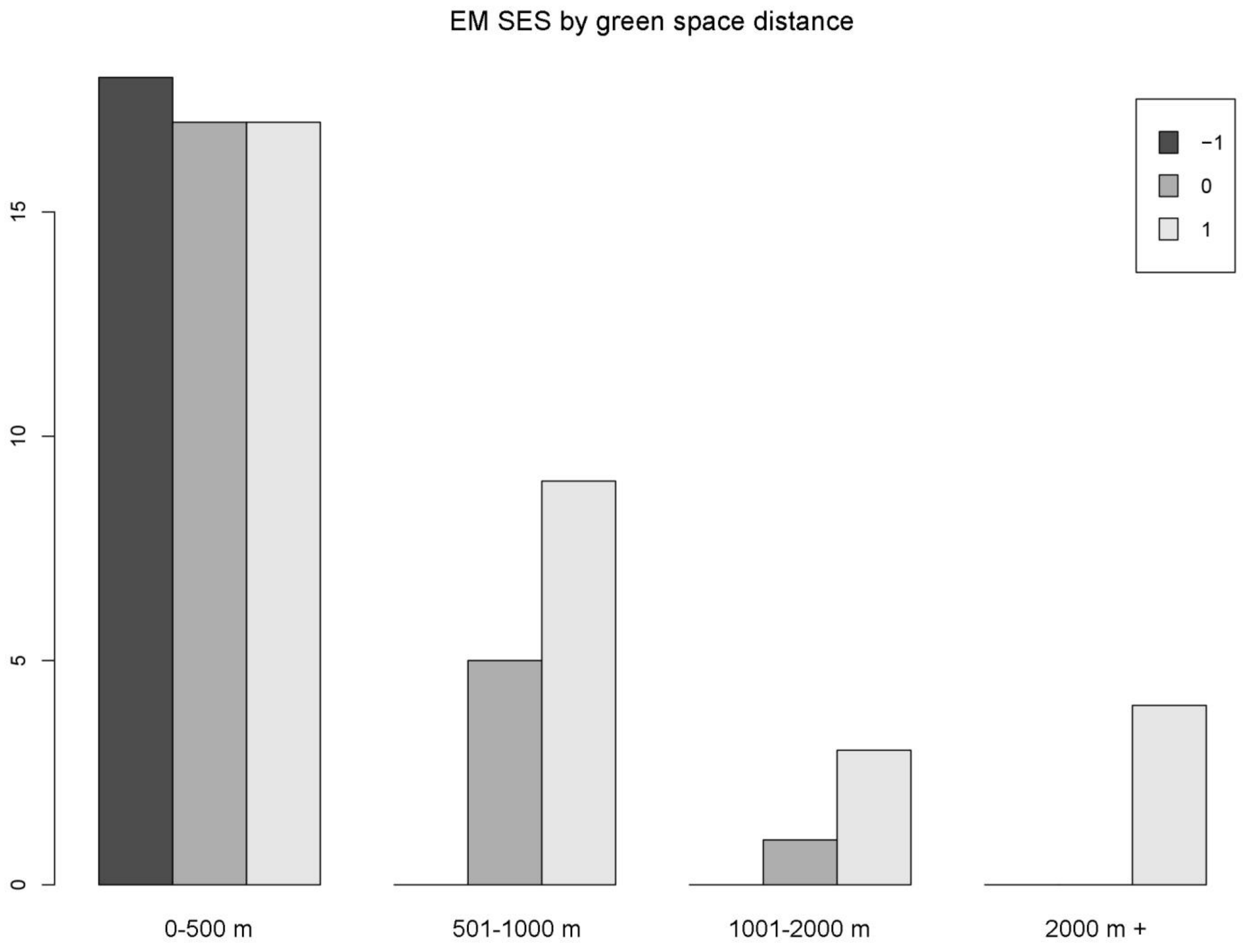

Figure S5. First sensitivity analysis: Effect modification (EM) for SES classified by green space distance. EM was recoded as -1 (high-SES people benefit more), 0 (no differences), or 1 (low-SES people benefit more).

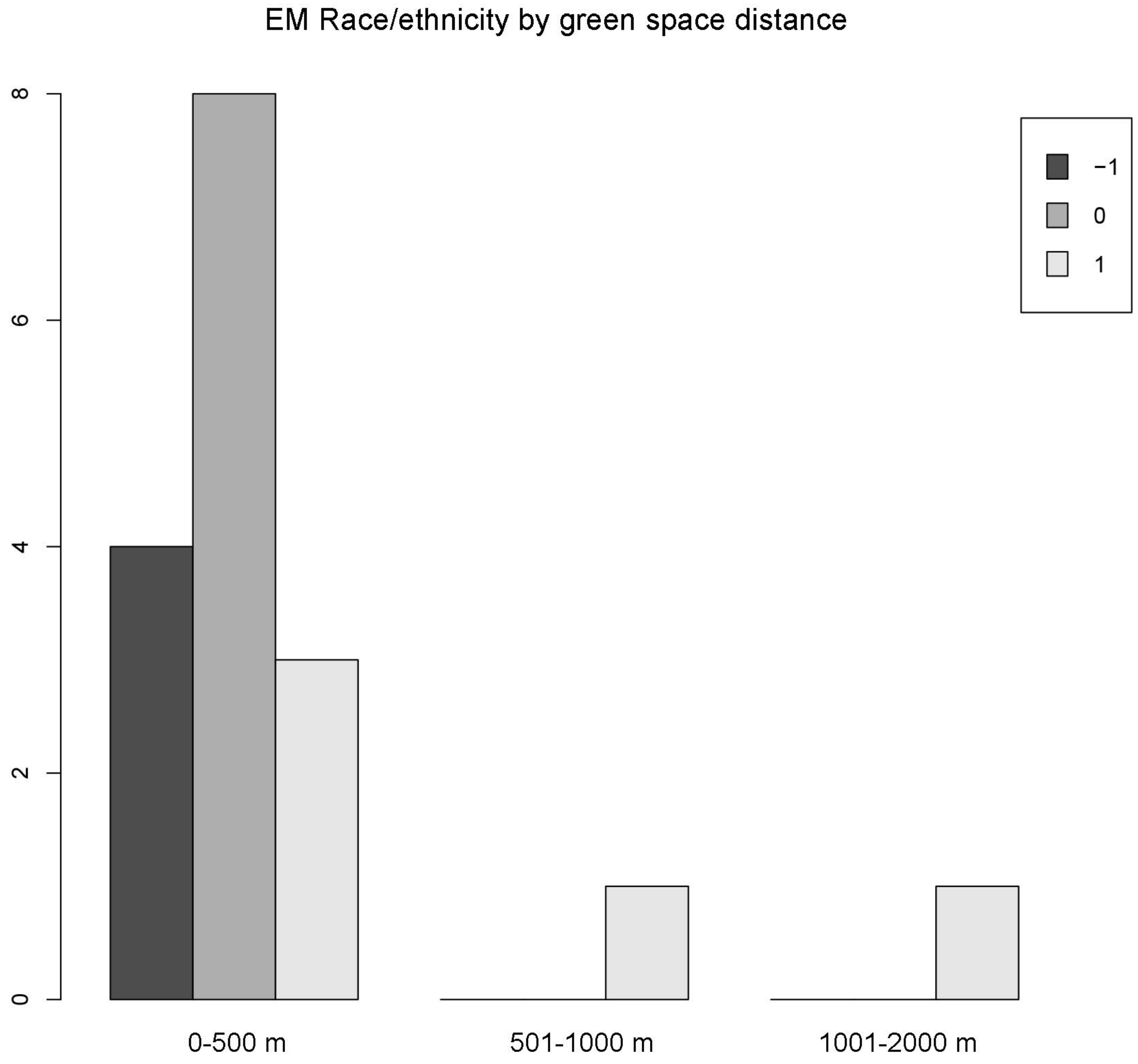

Figure S6. First sensitivity analysis: Effect modification (EM) for race/ethnicity classified by green space distance. EM was recoded as -1 (White people benefit more), 0 (no differences), or 1 (non-White people benefit more).

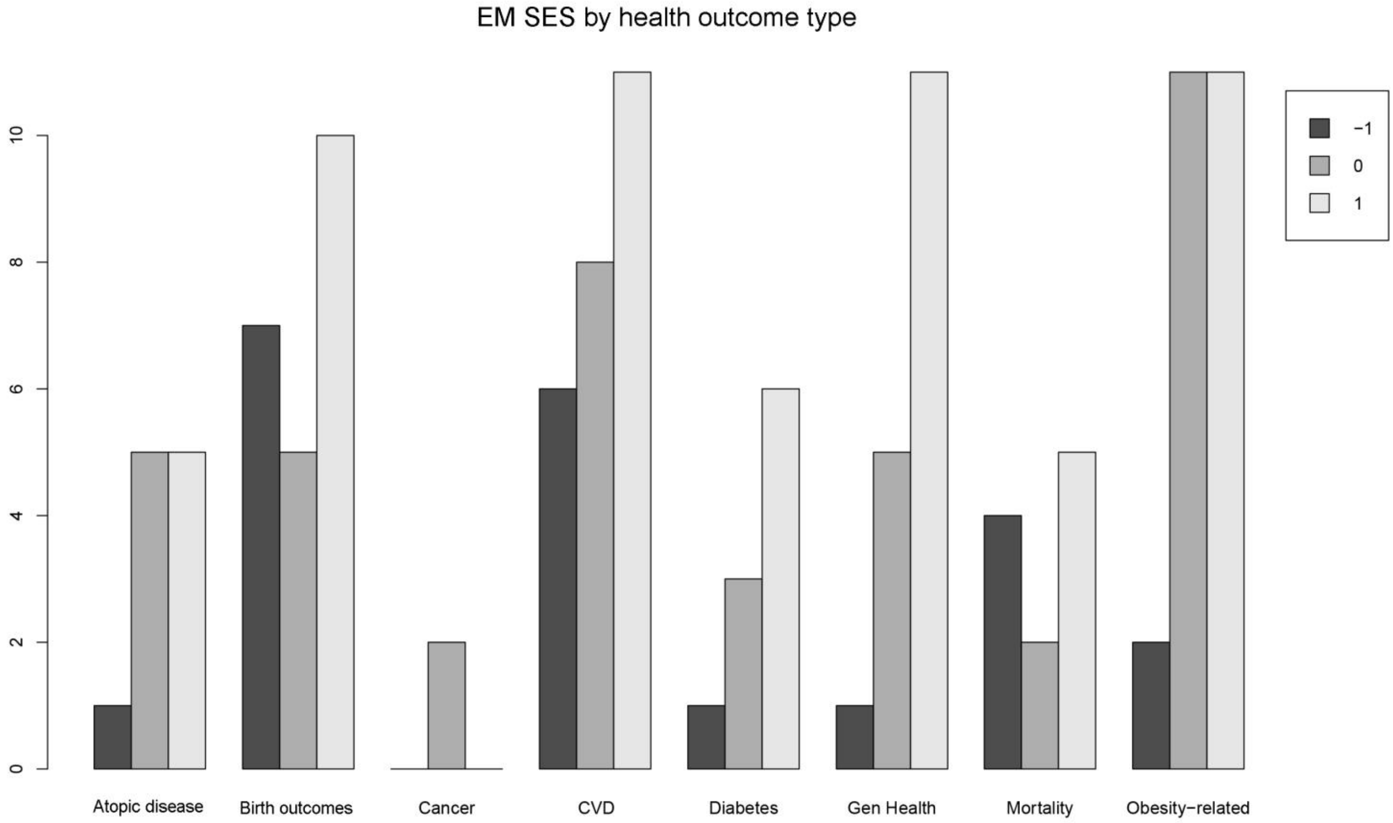

Figure S7. First sensitivity analysis: Effect modification (EM) for SES classified by health outcome type. EM was recoded as -1 (high-SES people benefit more), 0 (no differences), or 1 (low-SES people benefit more). CVD: Cardiovascular health/disease. Gen health: General health.

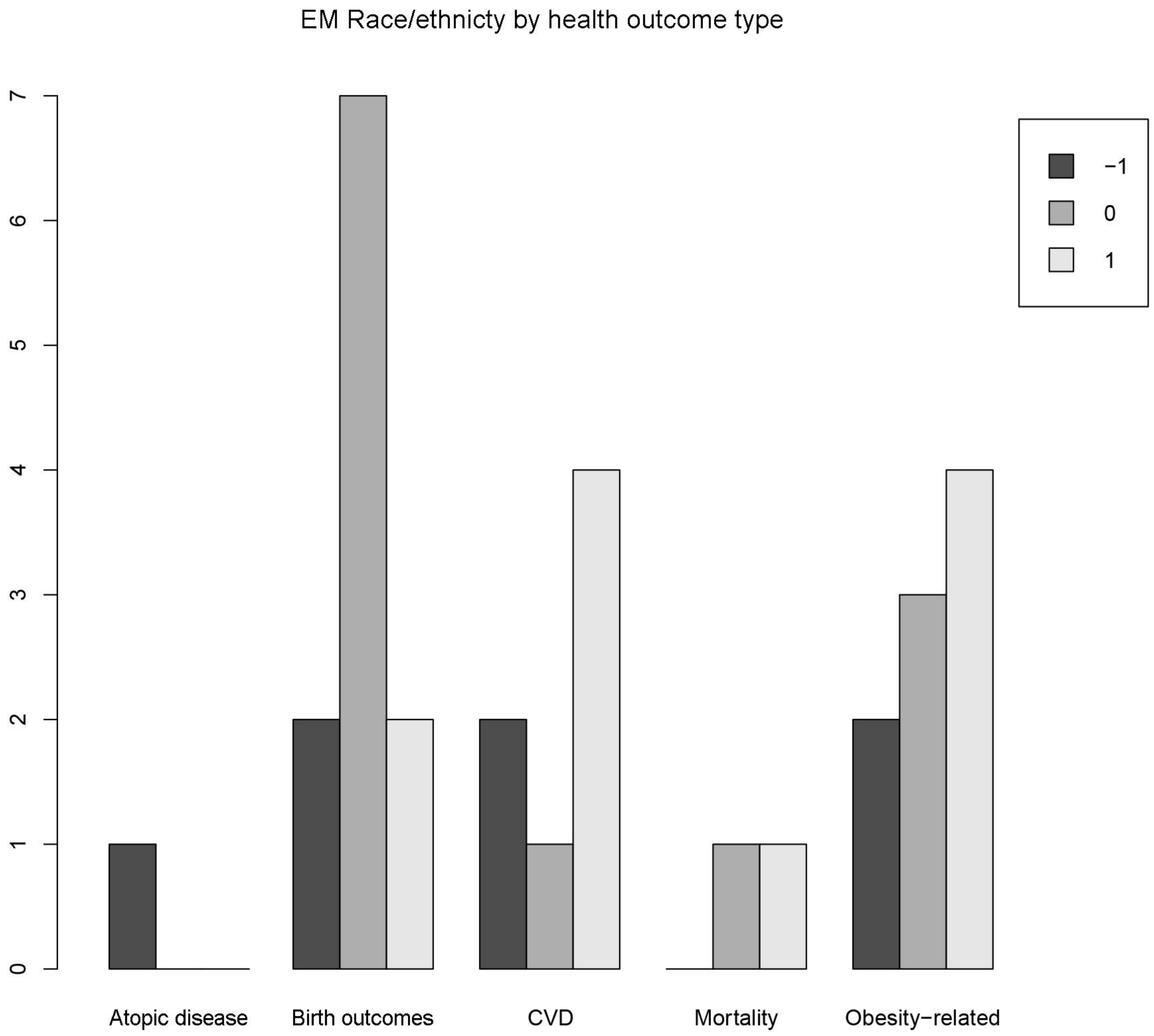

Figure S8. First sensitivity analysis: Effect modification (EM) for race/ethnicity classified by health outcome type. EM was recoded as -1 (White people benefit more), 0 (no differences), or 1 (non-White people benefit more).

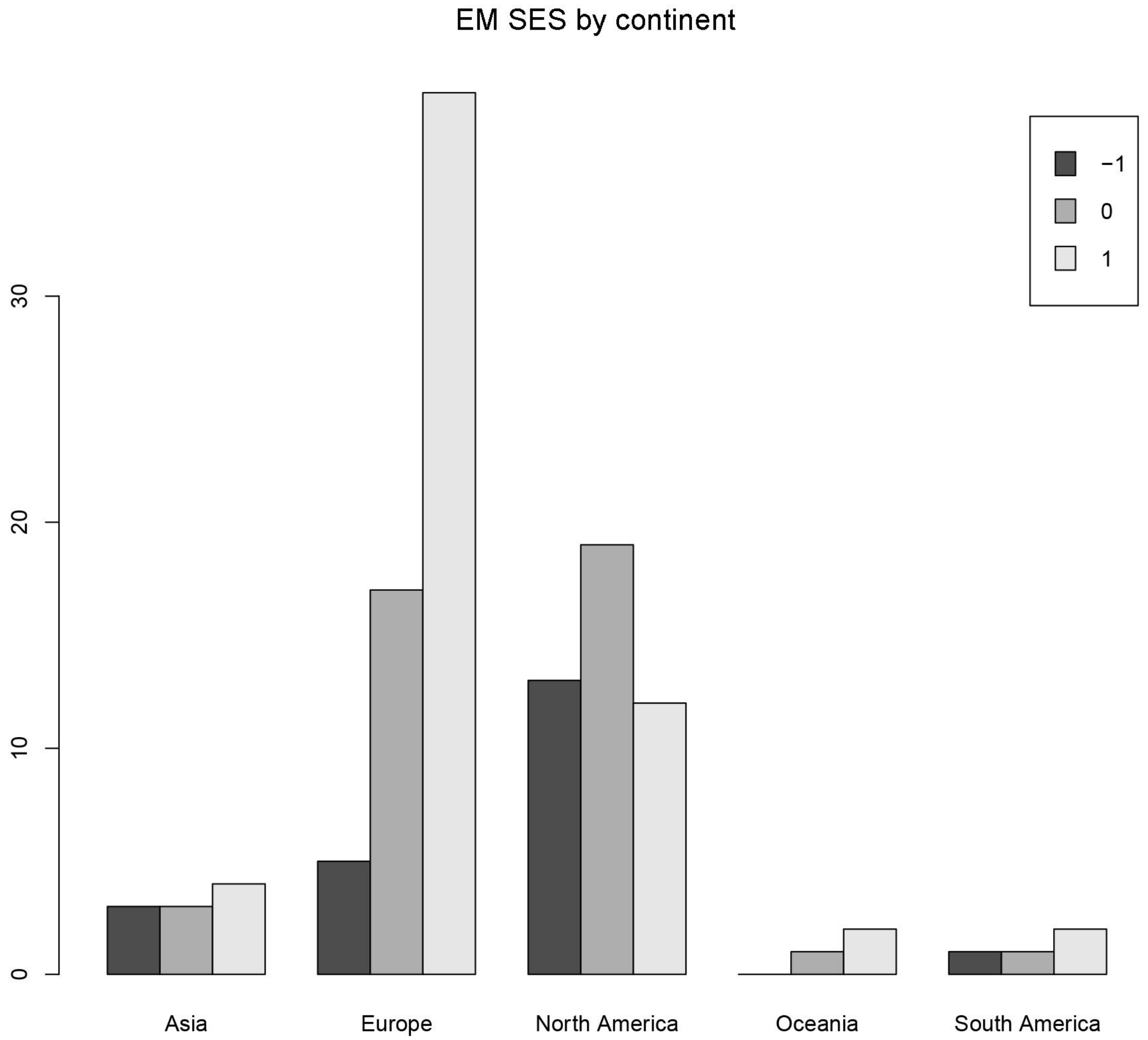

Figure S9. First sensitivity analysis: Effect modification (EM) for SES classified by continent. EM was recoded as -1 (high-SES people benefit more), 0 (no differences), or 1 (low-SES people benefit more).

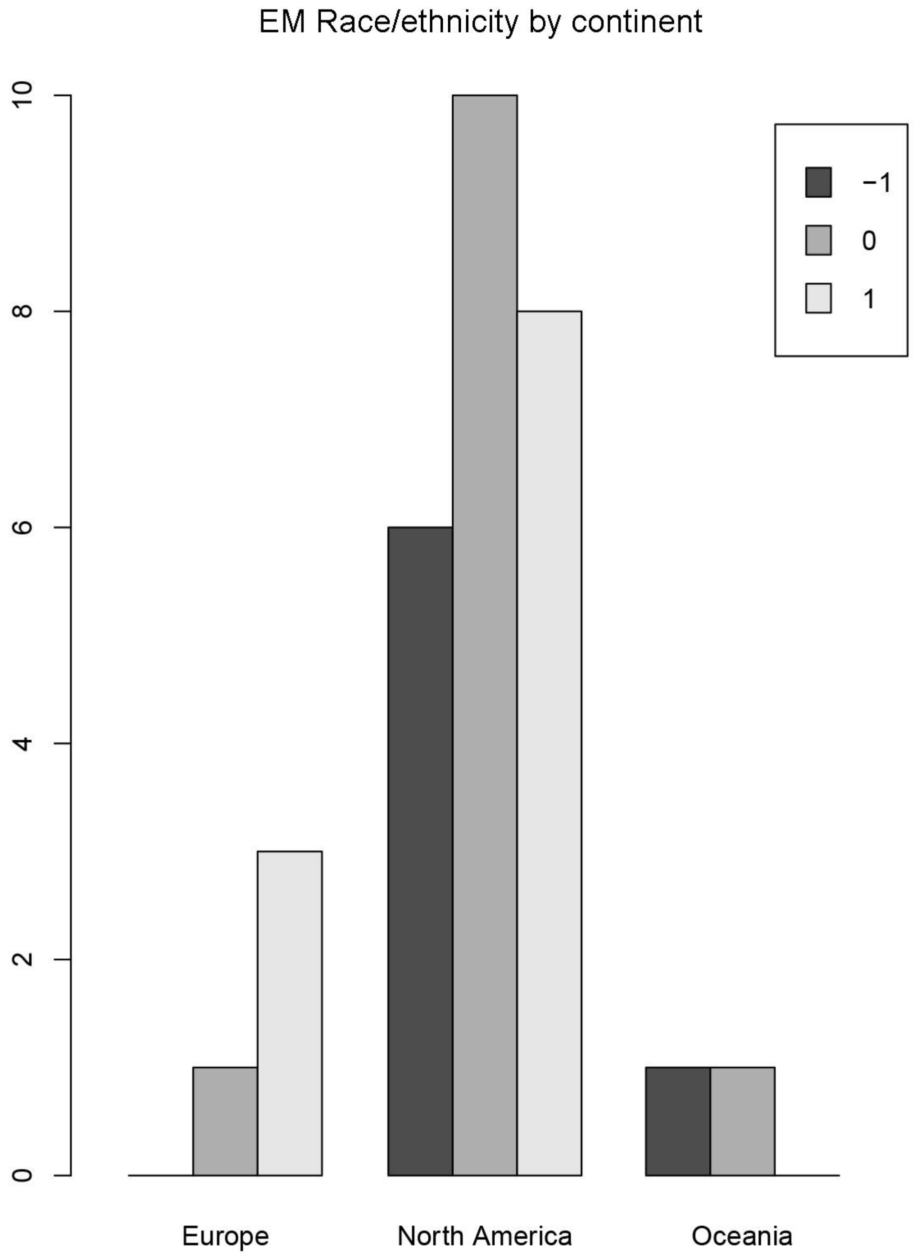

Figure S10. First sensitivity analysis: Effect modification (EM) for race/ethnicity classified by continent. EM was recoded as -1 (White people benefit more), 0 (no differences), or 1 (non-White people benefit more).
